# State of the Art and Future Perspectives in Advanced CMOS Technology

**DOI:** 10.3390/nano10081555

**Published:** 2020-08-07

**Authors:** Henry H. Radamson, Huilong Zhu, Zhenhua Wu, Xiaobin He, Hongxiao Lin, Jinbiao Liu, Jinjuan Xiang, Zhenzhen Kong, Wenjuan Xiong, Junjie Li, Hushan Cui, Jianfeng Gao, Hong Yang, Yong Du, Buqing Xu, Ben Li, Xuewei Zhao, Jiahan Yu, Yan Dong, Guilei Wang

**Affiliations:** 1 Key Laboratory of Microelectronics Devices & Integrated Technology, Institute of Microelectronics, Chinese Academy of Sciences, Beijing 100029, China; zhuhuilong@ime.ac.cn (H.Z.); wuzhenhua@ime.ac.cn (Z.W.); hexiaobin@ime.ac.cn (X.H.); linhongxiao@ime.ac.cn (H.L.); liujinbiao@ime.ac.cn (J.L.); xiangjinjuan@ime.ac.cn (J.X.); kongzhenzhen@ime.ac.cn (Z.K.); xiongwenjuan@ime.ac.cn (W.X.); lijunjie@ime.ac.cn (J.L.); gaojianfeng@ime.ac.cn (J.G.); yanghong@ime.ac.cn (H.Y.); duyong@ime.ac.cn (Y.D.); xubuqing@ime.ac.cn (B.X.); zhaoxuewei@ime.ac.cn (X.Z.); yujiahan@ime.ac.cn (J.Y.); dongyan2019@ime.ac.cn (Y.D.); 2 Research and Development Center of Optoelectronic Hybrid IC, Guangdong Greater Bay Area Institute of Integrated Circuit and System, Guangzhou 510535, China; liben@giics.com.cn; 3 Institute of Microelectronics, University of Chinese Academy of Sciences, Beijing 100049, China; 4 Department of Electronics Design, Mid Sweden University, Holmgatan 10, 85170 Sundsvall, Sweden; 5 Jiangsu Leuven Instruments, Xuzhou 221300, China; hushan.cui@gmail.com; 6 School of Cyberscience, University of Science and Technology of China, Hefei 230026, China

**Keywords:** CMOS, process integration, nano-scale transistors, epitaxy

## Abstract

The international technology roadmap of semiconductors (ITRS) is approaching the historical end point and we observe that the semiconductor industry is driving complementary metal oxide semiconductor (CMOS) further towards unknown zones. Today’s transistors with 3D structure and integrated advanced strain engineering differ radically from the original planar 2D ones due to the scaling down of the gate and source/drain regions according to Moore’s law. This article presents a review of new architectures, simulation methods, and process technology for nano-scale transistors on the approach to the end of ITRS technology. The discussions cover innovative methods, challenges and difficulties in device processing, as well as new metrology techniques that may appear in the near future.

## 1. Introduction

The down-scaling of complementary metal oxide semiconductor (CMOS) has followed Moore’s law for decades, where different parts of the transistor’s structure were shrank down by a constant factor in order to obtain lower power consumption [1]. However, the miniaturization trend of CMOS goes very differently nowadays as guided by an international roadmap for devices and systems (IRDS) [2]. By entering the data-centric era [3], the CMOS scaling is focusing more and more on low voltages, cost-effective processes, and high performance to meet the requirements of high-end mobile applications. Thus, several “new techniques” were introduced into the modern devices e.g., channel strain, stress boosters, high-κ metal gate, various silicides, etc. Beyond those inventions, the shape of CMOS has already been changed from planar to 3D by overcoming a lot of integration issues [4]. By entering the 10 nm technology node, pure silicon-based channel is being gradually replaced with silicon-germanium (SiGe) or germanium (Ge), and III-V materials, because they have better mobility of- 40,000 cm^2^V^−1^s^−1^ for InGaAs (for electrons) and 1900 cm^2^V^−1^s^−1^for Ge (for holes) compared to 1400 cm^2^V^−1^s^−1^ for electrons and 450 cm^2^V^−1^s^−1^ for holes of silicon [5,6]. Not only are the channel materials changing, but so is device shape, from simple fin-like to fully depleted on insulator or nanowire ones [7].

As a result, the most promising transistor architectures may be fin-on-insulator (FOI) Fin Field-Effect Transistor (FinFET) [8,9,10,11], scalloped fin FinFET [12], and NW field effect transistors (FETs) [13,14,15]. These new transistor designs have shown better control of short channel effects (SCEs), low leakage junctions, and high carrier mobility. One interesting property of FOI FinFET is the advantage that bulk FinFET and SOI technologies are merged in these transistors for a better platform in the technology roadmap. Moreover, a recent report from IMECAS has demonstrated a simple and cost effective fully metallic source and drain (MSD) process for FOI FinFETs with a gate length of 20 nm where I_on_ reaches up to 547 μA/μm and 486 μA/μm for NMOS and PMOS, respectively [9]. These results give an excellent potential solution for future nano-scale transistors. The scalloped fin FinFET including all previous HKMG technology also has a large control area and provides a great improvement of SCEs.

One of the most discussed approach for nano-scale transistors is vertical or horizontal gate-all-around (vGAA or hGAA) FETs. Many reports indicate these GAA-designs as excellent candidate for 3 nm node (and beyond) due to their device characteristics e.g., steep sub-threshold slop (SS), quasi-ballistic transport, and one-dimensional channel structure [13,14].

This article presents the essential issues for design and processing of the advanced nano-scale transistors following to the end of technology roadmap and beyond Moore. The content is divided into four parts:

Part one contains the design of hGAA-FET and vGAA-FET and device simulation including advanced TCAD for nanoscale transistor development. 

Part two covers process technology for nanoscale transistors. The discussions include advanced lithography for nano-scale patterning, epitaxy of SiGe as stressor material in S/D of nano-scale transistors or SiGe/Si multilayers for vGAA-FET, dopant implantation and adavanced doping technology, HKMG and metal gate using atomic layer deposition (ALD) technique, strain engineering using Si nitride films, etching of transistor structures and channel release, wet cleaning, BEOL interconnect materials, and process dependent reliability.

Part three presents the possible channel materials for beyond the Moore era. This part covers the growth and application of III-V materials and 2D crystal for high carrier mobility FinFETs, photodetectors, and sensors.

Part four contains metrology technologies, which can be used for in-line monitoring and nano-analysis including advanced electron microscopy, 3D atom force microscope techniques, 3D Atom probe tomography, X-ray techniques, optical critical dimension (CD), and hybrid metrology along with artificial intelligence.

The novelty of this review article stems from the well-organized description of scientific and technical issues of CMOS technology at the present time and in the future. The tactical approach provides unique knowledge for the readers about the challenges and difficulties to design, process, and finally to measure the nanoscale transistors.

**Part One: Advanced Designs of Nano-Scale Transistors**

## 2. This Part Covers the Transistor Designs to the End of Technology Roadmap and Beyond Moore

### 2.1. New Device Structure-Vertical Gate-All-Around FETs

Although FinFET has made enormous contributions to the IC industry since it was proposed in 1999 [16] and introduced into massive production in 2012 at the 22 nm technology node [17], it is difficult to meet the requirements of the performance and power consumption for further scaling down of device area. Gate-all-around (GAA) FET is a strong candidate for 3 nm technology, owing to its high performance and superiority in the control of SCEs [3,18]. In general, there are two kinds of GAAFETs, namely, horizontal GAAFET (hGAAFET) and vertical GAAFET (vGAAFET), depending on channel orientations. When the channel of a GAAFET is in parallel with the wafer/substrate surface, the transistor is called hGAAFET [19,20,21], but when the multiple horizontal channels are vertically stacked then the architecture is considered as multiple bridge channel FET (MBCFET) [22]. If the channel of a GAAFET is along the wafer/substrate surface, the GAAFET is called vGAAFET [23].

Intensive studies for hGAAFETs have been done by several research groups [13,15,19,20,21,22,24,25]. The first hGAAFET was presented by Colinge et al. [19]. In this field, the nanowire size-effects on the electrical properties were studied in reference [26] while how to enhance single device performance for the hGAAFETs with vertically stacked nanowire/nanosheet were presented in references [13,20,21,27]. In order to reduce parasitic capacitance and improve reliability, inner spacers were introduced [28] for hGAAFETs. High mobility materials, such as strained Si, Ge, InAs, GaAs, and InGaAs, were used to enhance the performance of hGAAFETs [29,30,31,32]. Compared with hGAAFETs, vGAAFETs have fewer constraints with respect to gate length and source/drain contact area [33,34] and have great potential for increasing the integration density [19,35]. In this section, we shall mainly focus on reviewing the progress of the vGAAFETs developed to explore novel device architectures and integration schemes more/beyond Moore applications.

**Figure 1:**
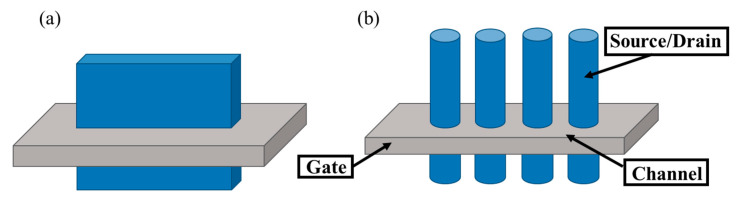
Schematics of vertical gate-all-around field effect transistors (GAAFETs): (**a**) Vertical nanosheet GAAFET and (**b**) vertical nanowire GAAFET. Vertical nanowires—Lg and spacer dimension are decoupled from the gate pitch. Density is determined by distance between the wires. Many process challenges. [37].

The typical structures of vertical nanosheet and nanowire GAAFETs are shown in Figure 1. The main advantage of vGAAFETs over hGAAFETs is that they have more integration freedom in the vertical direction than hGAAFETs do. The limitations of gate length, spacer width, and source/drain contact area for hGAAFETs [36] can be relaxed for vGAAFETs and then integration density is increased [37]. It is also good for vGAAFETs to use high mobility materials for their channels, which usually have small carrier effective masses and large tunneling leakage current, since relatively long gate length could be applied.

**Figure 2:**
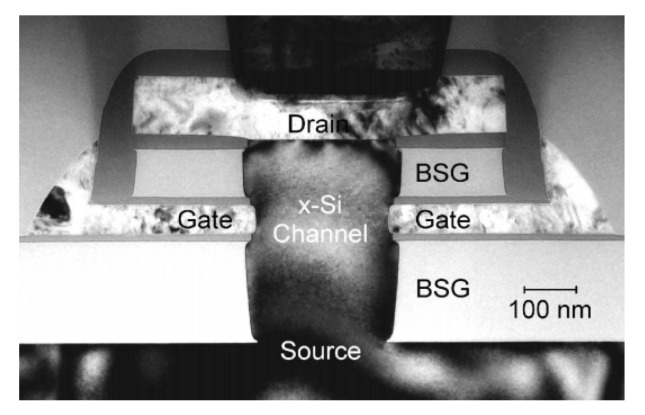
TEM cross-section of a Si channel vertical GAAFET (vGAAFET) with channel last or growth from bottom to top after the formation of bottom bottom borosilicate glass (BSG), gate layer, and top BSG layers [38].

Several fabrication methods for vGAAFETs were proposed, including bottom-up [38,39,40,41,42] and top-down [43,44,45,46] based on the ways to form vertical device channels. TEM cross-section for a Si channel vGAAFET with channel-last bottom-up method is shown in Figure 2 [38]. The method concerns forming bottom borosilicate glass (BSG), gate layer, and top BSG layers at first, and then etching channel holes through these layers stopping on the bottom Si. A Si nanowire was formed to refill the hole by selective epitaxial growth from the bottom Si. Source/drain was then formed by an annealing treatment to drive the dopants from the BSG layers into the epitaxial Si and self-align to the gate. The gate length was well controlled by the film deposition. This process is also compatible with the scheme of replacement metal gate. However, when the diameter of the channel hole becomes smaller and the aspect ratio becomes larger, especially for vertically stacked multi-device-layer structures, this makes it difficult to form high-quality single crystal channel, which is particularly important for high-performance logic circuits.

**Figure 3:**
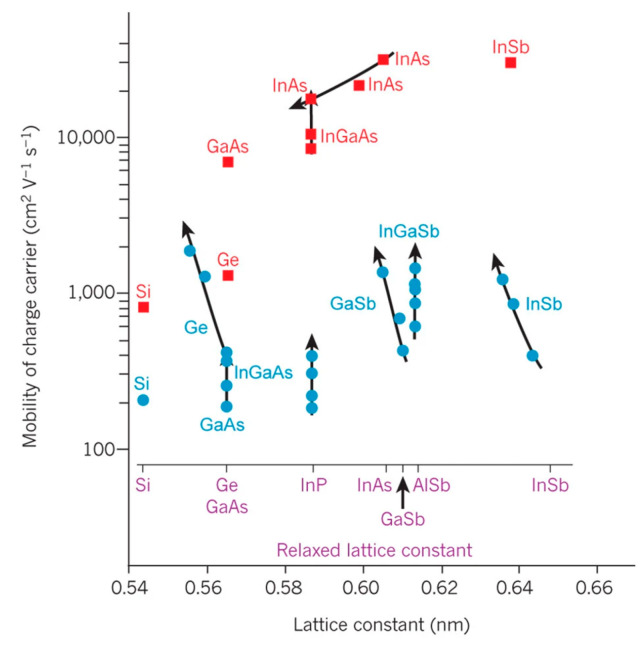
Carrier mobility in inversion layers and quantum wells in Si, Ge, and III-V compounds. Red symbols represent electron mobility and blue ones are marked for hole mobility. The electron mobility in the compounds shown in the plots are much higher than that in Si and Ge [50].

Another bottom-up method forms the channel at first, as several groups have demonstrated in III-V vGAAFET researches [41,47,48]. The vertical channel is highly relevant for III-V materials since they suffer from large off-current due to narrow band gap. The high electron mobility of III-V compounds and relaxation of gate length scaling may offer descent on-off ratio for III-V vGAAFETs. Therefore, III-V compound semiconductors have great potential to replace Si [49] as channel materials for nMOS [50] as shown in Figure 3.

**Figure 4:**
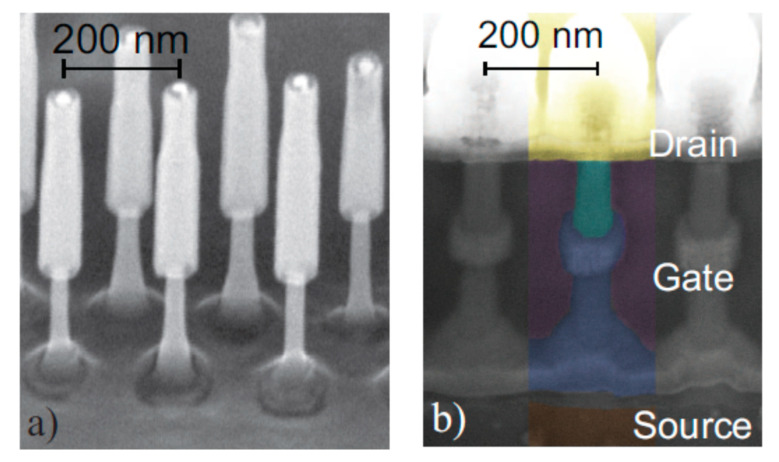
InAs vGAAFET formed by vapor-liquid-solid (VLS) bottom-up method: (**a**) Nanowires after thinning of channel regions and (**b**) final device structures with high-k metal gates [41].

**Figure 5:**
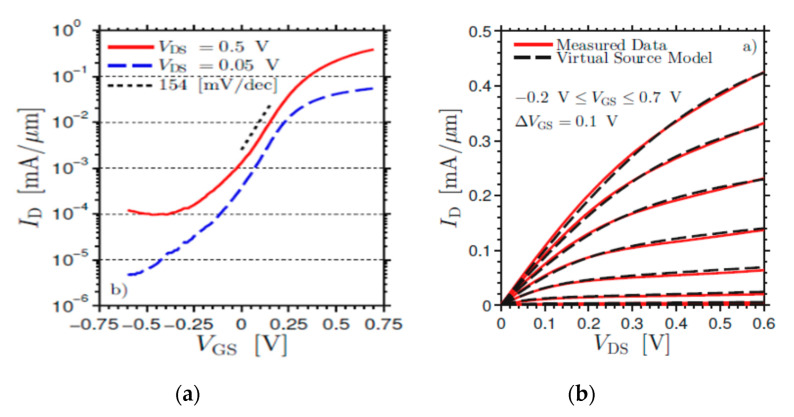
For an InAs vGAAFET consisting of 280 nanowires in parallel with a diameter of 28 nm and gate length of 190 nm: (**a**) Transfer characteristics and (**b**) output characteristics [41].

Figure 4 shows tilt views of the SEMs for InAs vGAAFETs fabricated successfully in reference [41]. The InAs vGAAFETs were fabricated on Si (111) wafer. The nanowires were grown using metal organic vapor-phase epitaxy (MOVPE) and using the vapor-liquid-solid (VLS) method from electron-beam the positions and size of the Au particles as the catalyst were defined. The method of VLS has been widely used to grow nanowires with different materials, such as Si, SiGe, InAs, and InP [51,52,53,54,55]. The transfer and output characteristics of an InAs vGAAFET consisting of 280 nanowires in parallel with a diameter of 28 nm and a gate length of 190 nm are shown in Figure 5, indicating excellent on- and off-device-performance.

**Figure 6:**
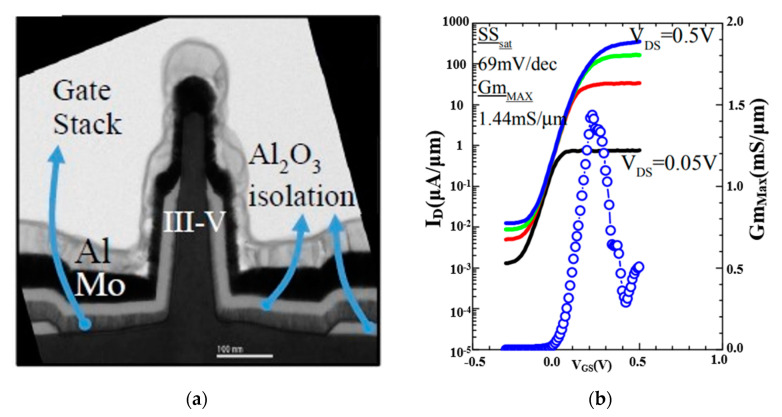
In_0.53_Ga_0.47_As nanosheet FET made by a top-down method: (**a**) TEM cross-section and (**b**) the transfer characteristics [45].

Top-down methods are employed to make vGAAFETs with the better reproducibility and less defectivity than bottom-up. For instances, the size of nanowires can be well defined by lithography while the channel can be formed on entire wafers [45] or high-quality films [31] instead of growth with small catalyst caps [41,55] or through small channel holes [38]. Figure 6a shows an InGaAs nanosheet FET made by top-down method [45]. The transfer characteristics of an In_0.53_Ga_0.47_As vGAAFET with channel length of 100 nm, diameter 35 nm, and CET 1.62 nm, are shown in Figure 6b. For these transistors, excellent *Q*-value (Gm/SS = 21) and I_on_ (= 379 uA/um @I_off_ = 100 nA) were achieved.

**Figure 7:**
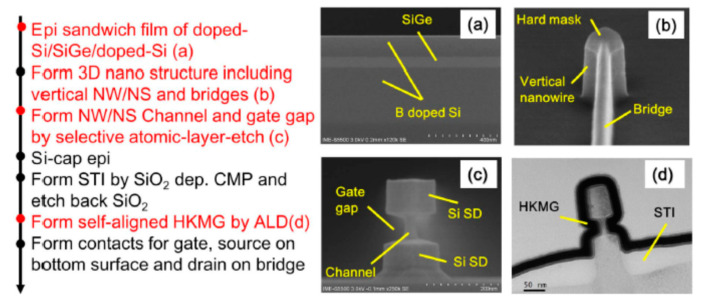
Process flow for vertical sandwich GAAFET (VSAFETs): (**a**) SEM after Si/SiGe/Si, (**b**) SEM tilt image of the 3D nanostructure after Reactive ion etching (**c**) SEM after quasi-atomic layer etching (QALE), and (**d**) TEM after high-k and metal gates (HKMG) deposition [46].

Compared with hGAAFETs, vGAAFETs have many new process issues since their fabrication process is quite different from FinFETs. A key challenge for the fabrication of vGAAFETs is to improve the precise control in vertical direction [56]. Gate length variation of vGAAFETs is a serious problem since it is usually controlled by the time of the etch process after gate metal deposition [39,45]. Although aniostropic deposition of gate metal was used to form 15 nm gate length for relatively large nanowire-pitch (NP) [57] or the deposition of HDP (high-density plasma) oxide film was utilized to determine gate length, it is still a big challenge when the NP is scaled down and the aspect ratio between nanowire height and NP is large. Another method to define gate length is that an electron-beam resist was spun on to cover nanowires and only the top part of the resist was exposed with electron-beam, where the exposed part is removed after development and the thickness of the left part of the resist and dielectric spacer is formed later on to determine the gate length [41]. Since the thickness of the resist is much thicker than gate length, the relatively large process variation, within-wafer or wafer-to-wafer, will be transferred to gate length variation. In addition, nonsymmetrical source/drain and misalignment of gate to channel may pose challenges to circuit design and cause the degradation of device performance. To address those problems, we recently reported a new integration flow to make vGAAFETs with self-aligned high-k metal gates and small effective-gate-length variation [46]. The vGAAFET is called vertical sandwich GAAFET or VSAFET. The integration flow with top-down method and the corresponding structures after key steps are shown in Figure 7.

**Figure 8:**
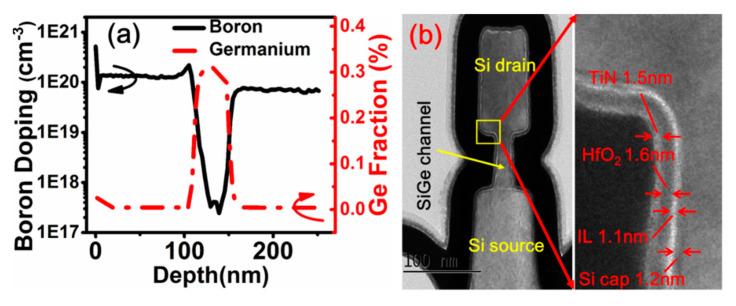
(**a**) Secondary ion mass spectrometry (SIMS) profiles of B and Ge in a p-Si/i-SiGe/p-Si sandwich film [31]. An abrupt B profile was achieved by in-situ doped epitaxial growth. (**b**) TEM cross-sections of an NS pVSAFET with an Si cap and an HKMG stack with interface layer (IL) = 1.1 nm [46].

The process starts with deposition of p-Si/i-SiGe/p-Si multi-layer on blank wafers. Later, nano-pillars and arms that connect the nano-pillars are formed by electron-beam lithography and anisotropic etch. A quasi-atomic-layer-etch [58] is developed and applied to selectively etch/recess the i-SiGe layer to form vertical nanowire/nanosheet channels with precise size control but at the same time to achieve gate-position self-alignment. Gate length is well-defined by the thickness of epitaxial i-SiGe layer. SIMS profiles in the p-Si/i-SiGe/p-Si film and TEM cross-sections of an NS pVSAFET are shown in Figure 8. Excellent electrical properties of an NS device with channel thickness of 20 nm and gate length of 60 nm were obtained: Drain-induced barrier lowering (DIBL) = 40 mV, SS_sat_ = 86 mV/dec, I_on_ = 37.6 uA/um, I_on_/I_off_ = 1.8 × 10^5^, and V_d_ = 0.65 V.

**Figure 9:**
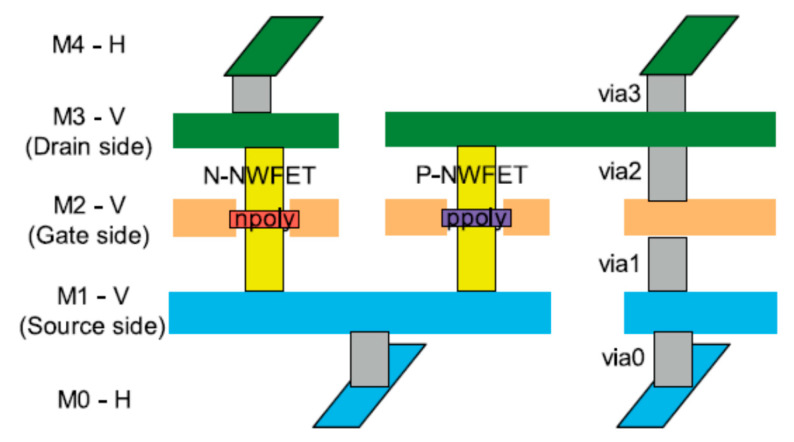
A design of buried interconnects for vGAAFETs [59].

To increase integration density, it is necessary to re-design local and global 3D interconnect [56]. Interconnection delay becomes more and more of a concern for IC performance. Comparing vGAAFETs with hGAAFETs and FinFETs, it was reported that vGAAFETs achieves significant reduction of parasitic capacitance and wirelength [59]. A design of buried interconnects for vGAAFETs was proposed as shown in Figure 9 [60]. In the design, the lateral interconnects, M1-V and M3-V, for both Source and Drain sides were used. It turned out that it is necessary to use M0-H as routing as well as to use layers above M3. The proposed structure is based on the importance of the Source side (S: M1-V) as well as the Drain side (D: M3-V) in the routing perspective. Compared with hGAAFETs, averaged over 12 standard cells, the use of vGAAFETs achieves 22.5%, 14.4%, and 28.4% reductions of area, wirelength, and capacitance, respectively. 

Another advantage of vGAAFETs is that it can realize multi-layer vertical stacking, thereby further reducing the area of standard cells. In the simulation of Satish Maheshwaram et al. [60], two-layer stacked vGAAFETs can reduce the area by 20% compared to single-layer CMOS.

### 2.2. Tunneling Field-Effect Transistor (TFET) Approach

To reduce power consumption of metal oxide field effect transistors (MOSFETs) without degradation of device performance, it is required that MOSFETs’ operating voltage (V_dd_) and threshold voltage (V_th_) are simultaneously scaled down. The leakage current or power consumption is increased due to lowering V_th_ if the sub-thresheld swing (SS) of MOSFETs can not be reduced. Therefore, it is desirable to reduce the value or to break thermal dynamic limit, 60 mV/dec, of the SS. The tunneling field-effect transistor (TFET), which is based on band-to-band quantum tunneling, is one of the most promising steep slope devices for low power applications [61].

**Figure 10:**
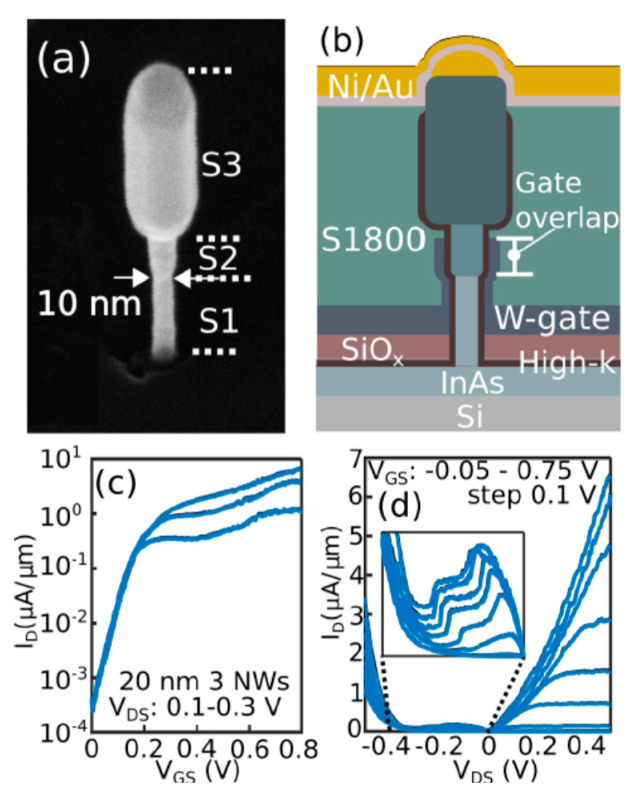
(**a**–**d**) Tunneling field-effect transistor (TFET) characterization: (**a**) SEM of a nanowire (NW) with a diameter of 10 nm when the layers are S1 = InAs, S2 = InGaAsSb, and S3 = GaSb, and (**b**) schematic of TFET structure in (**a**,**c**) transfer characteristics of a TFET with diameter of 20 nm, and (**d**) output characteristics of the same transistor in (**c**) [63].

To achieve good tunneling performance, a vertical Si TFET was fabricated and tested in 2000 [62]. Different semiconductor materials (Si or III-V) have been used for TFETs [62,63]. Vertical InAs/InGaAsSb/GaSb nanowire GAA TFETs, whose channel diameters are 20, 15, and 10 nm, were made and operated with SS well below 60 mV/decade [63]. The fabricated devices and I–V characteristics are shown in Figure 10.

## 3. Advanced TCAD for Nanoscale Transistor Development

Technology computer-aided design (TCAD) takes advantages of physical models and modern high-performance computing powers to provide predictive perspective in semiconductor technology research and manufacturing. TCAD effectively bridges the underlying physics and the realistic device merits, presenting guidelines for process and device development. Advanced TCAD technology with enough accuracy and affordable simulation burden is crucial both in academy and industry so as to address the device physics, shorten the technology development cycle, and reduce the fabrication cost. Furthermore, the industry highly demands the application of TCAD beyond the integration phase into manufacturing, as well as to yield optimization as well. A standard TCAD sequence involves process simulation, device simulation, and circuit simulation. Since this section cannot cover the complete TCAD technologies and their application spaces, it will only focus on typical device models in a hierarchy based on different levels of precision and complexity.

TCAD Device simulation calculates the behavior of electronic devices, e.g., the current–voltage (I_d_–V_g_, I_d_–V_d_) characteristics and frequency response (C_gg_–V_g_) of a device in general. The devices are defined and characterized mathematically in terms of geometries, materials, connected electrodes, doping, stress profiles, and other relevant physical parameters, all of which may be obtained from measurement, process simulation, or simply manual definition.

**Figure 11:**
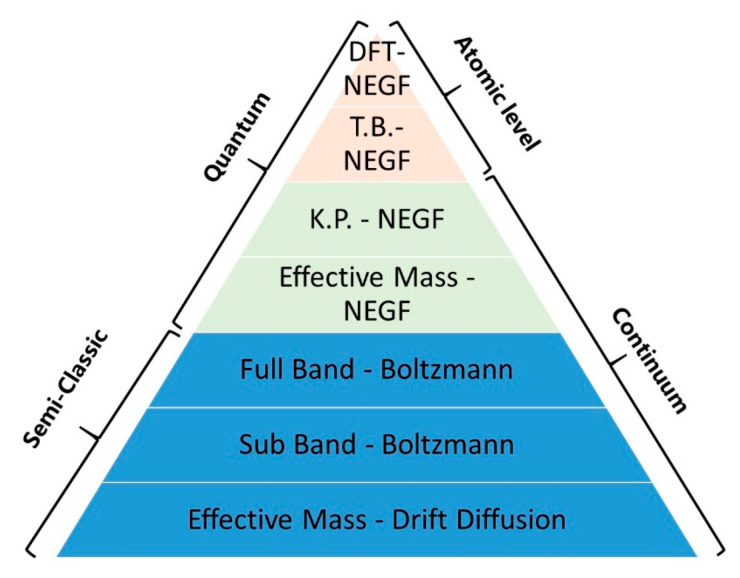
Illustration of the hierarchy of technology computer-aided design (TCAD) device models [64].

There are two main kernels in TCAD solvers for all levels of the model hierarchy, i.e., (1) the transport equations solver coupled with electrostatic solver, and (2) the Maxwell’s equations (Poisson equations) solver for fields (the quasi-static electric fields). The two kernels are coupled strongly and should be solved iteratively and simultaneously. The second kernel is fixed and transferable in versatile applications. Hence, in the following, we focus on the first kernel, which adopts different levels of approximations to leverage the model precision and complexity. V. Moroz from Synopsys Inc. summarized the typical electrostatic and transport models concisely into a pyramid [64], covering industry TCAD to state-of-the-art academical TCAD as shown in the Figure 11. We refer interested readers to the literature for more detailed hierarchy of TCAD device models [65]. Specifically, as the modern transistor dimensions approach atomic scale, the need to calculate quantum effect has impacted electrostatic and transport and evaluating the atomic-scale defects has motivated TCAD from engineering at the bottom in Figure 11 towards research at the top. Rigorous solutions to Schrodinger’s equations based on nonequilibrium Green’s function (NEGF) and density functional theory (DFT), semi-classical solutions of the BTE and continuum models such as drift-diffusion (DD) are elaborated to a multi-scale TCAD Framework [66].

**Figure 12:**
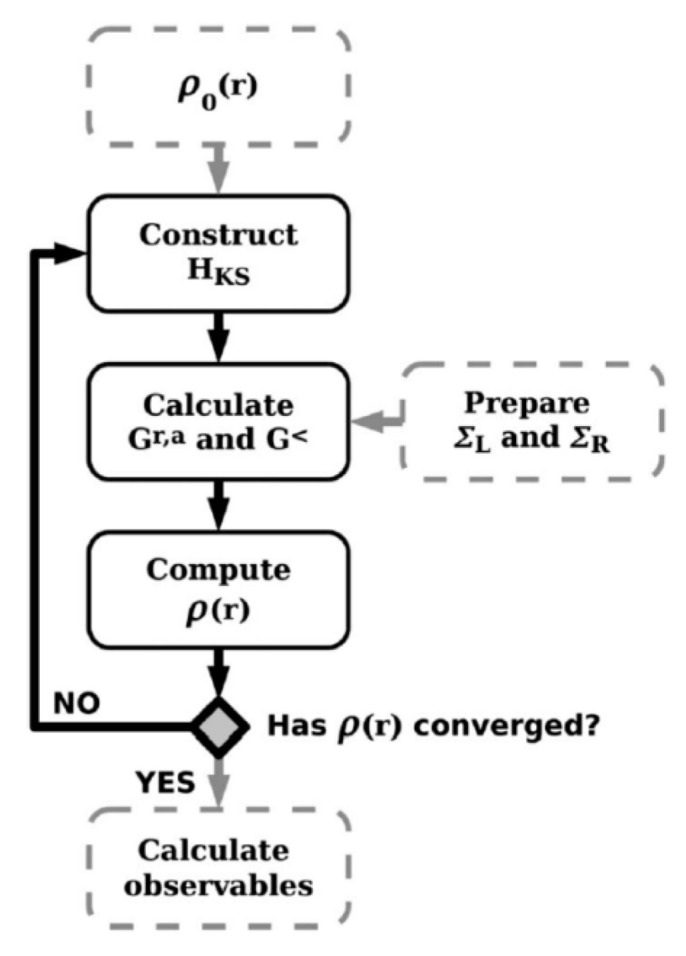
Illustration of the density functional theory (DFT)-nonequilibrium Green’s function (NEGF) self-consistent simulation flow [67].

The most accurate but complex TCAD solution is the DFT within the NEGF formalism (DFT-NEGF) method. In addition, the incorporation of DFT enables parameter free simulations, which is suitable for path-finding research of emerging technologies. The basic idea of the DFT-NEGF method is firstly proposed by H. Guo’s group [67], i.e., using the DFT to calculate the Hamiltonian and electrostatic properties of the device; using NEGF to determine non-equilibrium statistics for constructing density matrix; and using real Space numerical methods to calculate transport properties and the boundary conditions for open device structures (see Figure 12). It has been used as the standard approach to model nonequilibrium quantum transport in atomic nanostructures. High precision can be achieved by using DFT-NEGF, but at the expense of computational issues in speed and memory limits.

**Figure 13:**
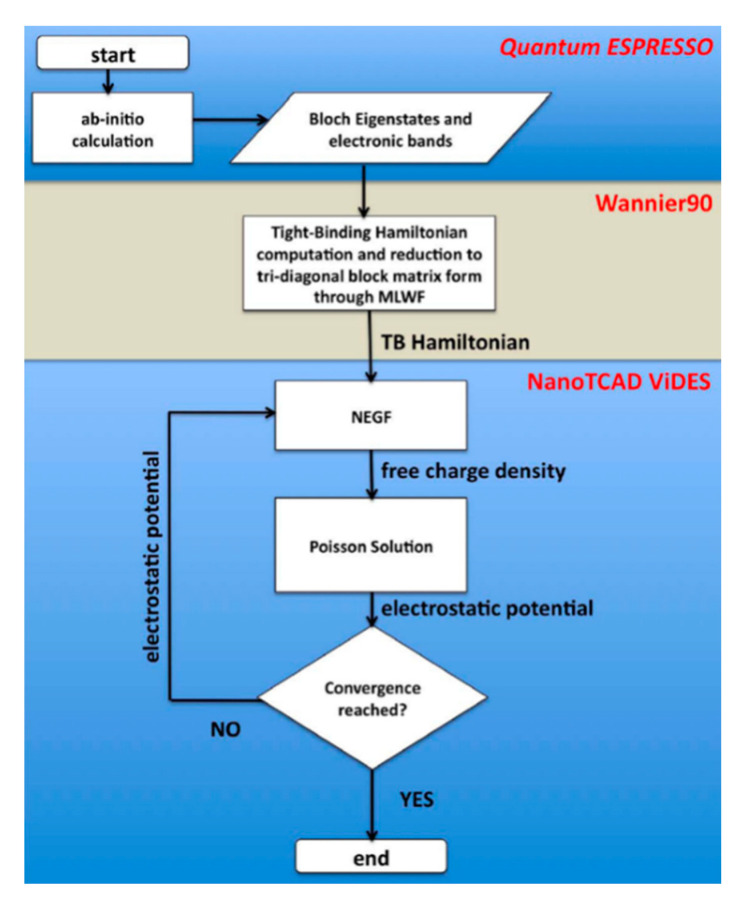
Illustration of the tight-binding (TB)-NEGF self-consistent simulation flow [70].

As a compromise, one may apply semi-empirical k.p (KP) or tight-binding (TB) models in combination with NEGF. Excellent transport results can be obtained if the KP/ TB parameters are properly chosen. Many KP/TB-NEGF codes with various capabilities and accuracies have been developed [68,69]. A standard open source TB-NEGF framework is proposed as shown in Figure 13 [70]. Several open commercial tools are also available [71,72]. Such schemes are able to solve quantum transport problems involving extended number atoms rather than DFT-NEGF method. Such KP/TB/DFT-NEGF models have been widely implemented in simulating the quantum transport phenomena in ultral scaled MOSFET as well as in studying the tunneling field effect transistors.

**Figure 14:**
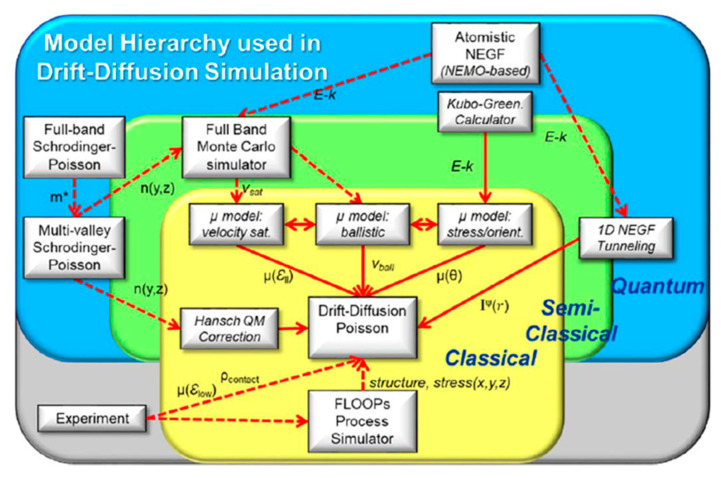
Ilustration of the advanced drift-diffusion (DD) calibration and applications [73].

Despite high accuracy in rigorous atomistic techniques such as the aforementioned DFT-NEGF and KP/TB-NEGF, they remain too computationally expensive for massive TCAD support in applications of industry development. Drift-diffusion (DD) is still preferred if the empirical parameters are obtained from advanced calibration scheme. In practice, for a given node, the optimized geometry is almost reached and only small variation in device dimension is needed. Thus, the design window is relatively narrow, in which calibrated DD model can give enough predictive results even for sub 10 nm node transistors. Moreover, the DD solution in well established in industry TCAD. It can handle realistic device geometries and is smoothly connected with the process and topographical simulations data. Due to the above reasons, the DD model still plays the most import role in engineering and continues to be infused with more rigorous models by advanced calibrations, affording the fast and massive data for industry technology development. For example, Figure 14 shows the DD TCAD solution proposed by Intel [73].

In brief, with shrinking device dimensions, the demand for accurate solvers of the coupled NEGF transport equations and atomistic-based electrostatic calculations has grown significantly. TCAD device models of different complexity, precision, and accuracy are developed in many academic groups or offered in various commercial tools. Depending on specific device scales, a quantum-mechanical model or a semi-classical model has to be adopted properly. Once the computational issues of speed and memory limits are relieved, the application domain of quantum transport modes can be further extended.

**Part Two: Process Technology for Nano-Scale Transistors**

## 4. Advanced Lithography Process

The most important goals for advanced lithography technology are to create more clear patterns and high reproducibility at the same time. For 20-nm and 14-nm node technologies, 193 nm ArF immersion with multiple patterns has been mainly used in manufacturing of FinFETs [74]. Further, 193 nm immersion with self-aligned double pattern (SADP) and self-aligned quadruple pattern (SAQP) technology aim for 7 nm technology nodes and below. For the advanced technology node, quadruple patterning needs to be further developed as it will increase the cost and process variation.

Extreme ultraviolet (EUV) lithography has been recognized as a promising candidate for the manufacturing of semiconductor devices as line space (LS) and contact hole (CH) patterns for 7 nm node and beyond. EUV lithography enables the use of single mask exposure instead of double or quadruple exposure. It will replace some key process steps in the flow for multiple patterning.

EUV lithography is ready for introduction and running in volume for technology development, but it still faces several challenges. EUV is used for metal layers at 7 nm node, but it still requires double patterning with traditional immersion steppers for some metal layers. Single-patterning process production cost is greatly lower than that of “multi-patterning” of repeated pattern circuits.

### 4.1. EUV Source

EUV light source power is critical for high-volume manufacture (HVM) and it can limit the large-scale use of EUV technique. To increase the throughput in HVM, the resist sensitivity to the 13.54-nm wavelength radiation of EUV needs to be improved while the line-width roughness (LWR) specification has to be kept to a few nanometers. In principle, an EUV stepper using a 250 W source and 25 mJ/cm^2^ resist, sensitivity should be able to process at least 100 wafer-per-hour (wph), which makes it an affordable price to match with the other lithography techniques. In fact, it is the light source that enables economical production capacity of the exposure tools. The latest ASML NXE3400B system can generate 250 W EUV light source [75]. This enables wafer throughput up to 140 wph where it is already acceptable for HVM [76]. In any way, the source power subsystems toward 500 W will be a huge challenge, but its wph will be about twice the current value [77,78].

### 4.2. EUV Resist

**Figure 15:**
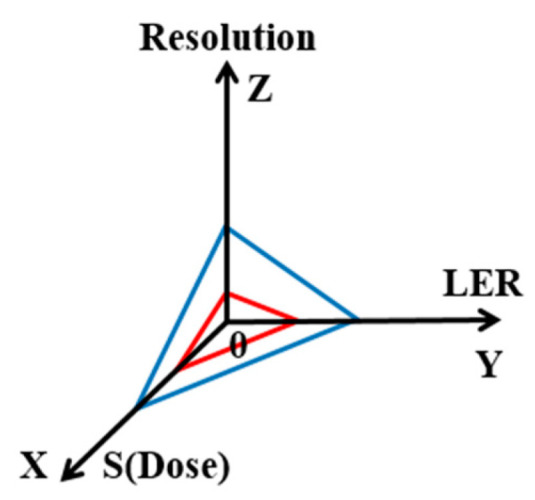
Resolution, dose sensitivity, and their relationship to low line edge roughness (LER).

For mass production of integrated circuit transistors, EUV resist requires significantly increased sensitivity and improved line edge roughness (LWR) and local critical CD uniformity (LCDU). The main challenge of EUV resist is to simultaneously require ultra-high resolution (R), low line edge roughness (LER), and high dose sensitivity (S) [79]. EUV photoresist materials need to be improved with better resolution and simpler chemistry to reduce photon shot noise and chemical fluctuations. The placement of individual molecules of chemical amplification material starts to be important at 10 nm node. Resolution, line edge roughness, and sensitivity are the critical technical issues of EUV lithography, and they are correlated with each other as shown in Figure 15. For example, when the LER increases, high dose is needed and, consequently, the resolution becomes low. The higher absorption requires the use of thinner resist, which mitigates the issue of line collapse. 

Another critical challenge is to develop suitable EUV photoresists at high resolution with high sensitivity and low line-width roughness (LWR) for the reduced film thickness needed for high-NA by its reduced depth of focus (~1/NA^2^) [80].

### 4.3. EUV Mask

The EUV mask infrastructure requires the manufacture of defect-free photomasks, where the ability to inspect photochemical masks is a key success factor [81,82]. Since protective films cannot currently be used in EUV lithography, other measures need to be taken to deal with the contamination that may be introduced during mask transportation and when they are being used. The defects caused by this contamination will significantly reduce the yield in the manufacturing of semiconductor devices [83].

In EUV system, the masks operate in reflective mode. In order to obtain maximum reflection, we need to improve material for the EUV absorber on the mask (which is mirror-like).

The multilayer Mo/Si are used as high-reflectance mirrors. The key properties include the roughness and inter-diffusion depth at the Mo-Si interfaces [84]. TaBN is the current standard absorber, but for low*-k1* imaging, the three-dimensional effect of the mask results in degradation of image contrast and edge placement errors. Mitigation of 3D-mask effects is a requirement for high-NA (0.55) EUV lithography [85]. Both the absorber and the reflective multilayer parts of the EUV mask contribute to the 3D-mask effects. 3D-mask effects are more predominant for wafer image quality. EUV pellicle is a thin membrane and it has been enabled to prevent reticle from contamination and particles, both inside and outside the scanner environment. However, it will cause reduction in throughput because of the absorption of EUV photons in the pellicle [86]. Therefore, throughput will be reduced when EUV mask pellicle is used.

### 4.4. Change to High-NA

**Figure 16:**
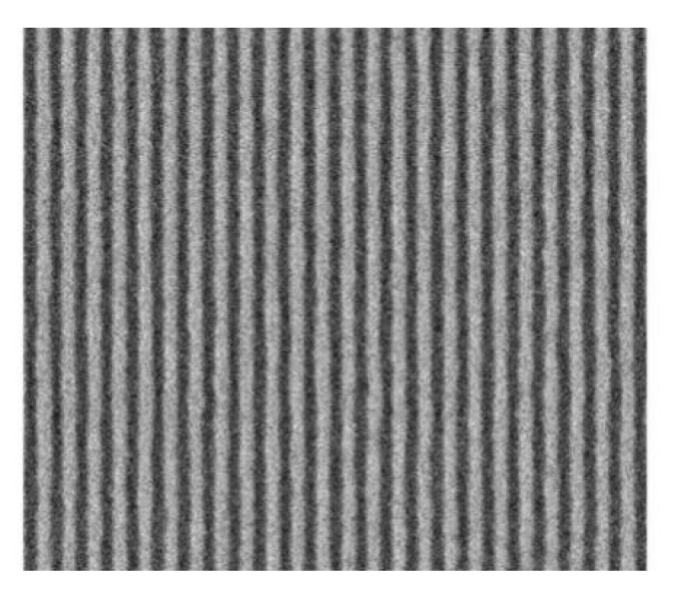
An image of 24 nm pitch lines which are successfully implemented through extreme ultraviolet (EUV) single-patterning process [88].

A 3nm EUV single-patterning process was available through a next-generation “high-NA” lithographssssssy system, which increases the numerical aperture (NA) of the EUV projection optics, from 0.33 to 0.55 [87]. As shown in Figure 16, ASML and IMEC were able to pattern 24 nm pitch lines using 3 nm process and the current EUV lithography system by adjusting an incident angle of EUV light source [88]. However, the high-NA will reduce the depth of focus, which leads to improve the focus control possibility.

## 5. Epitaxy of Nano-Scaled Transistors

### 5.1. SiGe Selective Epitaxy Growth (SEG) for Source and Drain (S/D)

**Figure 17:**
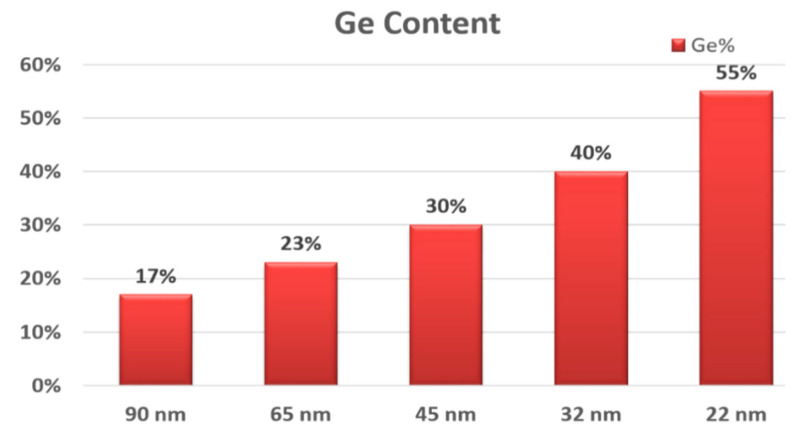
Ge contents in source and drain (S/D) regions in different technology nodes [92].

In the 90 nm technology node, selective epitaxy growth (SEG) of SiGe was integrated in the recessed S/D areas as stressor material to induce uniaxial compressive strain to enhance the carrier mobility in the channel region [89]. The Ge content in the SiGe in S/D areas has been increased from 19% to 45% while the critical dimension (CD) of the transistors were scaled down from 90-nm (2D: planar transistors) to 22-nm (3D: FinFET) nodes [89,90,91]. It has been reported that the Ge content in the SiGe S/D will be further increased to more than 50% in the 10 nm Fin structure [92]. A summary of the Ge contents in S/D areas for different technology nodes is shown in Figure 17.

In order to induce more strain in the channel area, the original round shape of recess (or trenches) in S/D regions was changed to sigma (“∑”) shape in the 45-nm node. It is because that the SiGe layers could be placed closer to the channel area [93,94].

The SEG process of SiGe on Si Fins and Si nanowires suffers from different challenges and problems: Defects due to strain relaxation [95], facet formation [96,97], non-uniform strain distribution [98,99], and the pattern dependency effects [100,101,102,103]. 

**Figure 18:**
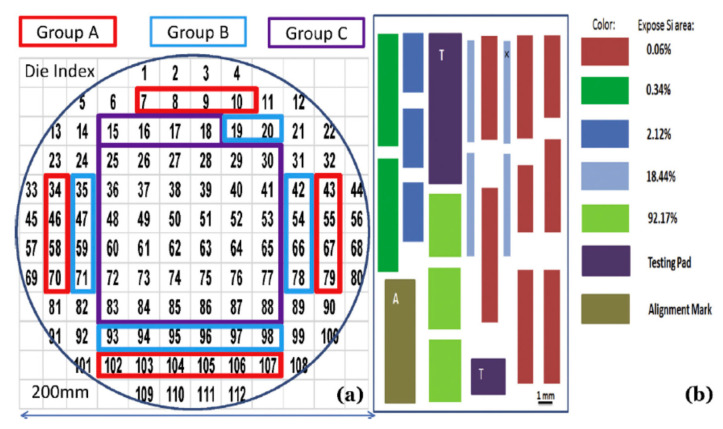
(**a**) The image of the processed chips on an 200 mm wafer where three groups were distinguished according to the transistor performance and (**b**) illustrates the pattern of one 112 manufactured chips where the different exposed Si areas are shown in different colors [94].

Figure 18a,b shows three groups of chips (A, B, and C) in terms of transistor performances (poor, good, and excellent for A, B, and C group, respectively) over the wafer. The X-ray results show the Ge content, layer thickness, and strain relaxation for transistors in these groups, which could be a result of the pattern dependency of selective growth [94].

The reason for the pattern dependency over a wafer (global effect) or inside a chip (local effect) is rooted from non-uniform consumption of reactant gases when the layout is changed. In a more specific way, pattern dependency of SEG occurs when the exposed Si area or opening size of the S/D regions are altered in a chip or when the neighboring chips have different layouts. A remedy to this problem is modeling the growth for a specific layout to find out the layer profile in advance and then change the chip layer out to create a uniform gas consumption over the chip [100,101,102,103].

The quality of the epitaxial layer and the strain induced by SiGe S/D to the channel is very sensitive to the quality of initial Si surface. This means that surface cleaning and treatment of Si-fins prior to the epitaxy are very important issues that determine the defect density of the SiGe layers. A standard cleaning method using chemicals—sulfuric acid and hydrogen peroxide mix (SPM) followed by Ammonium hydroxide and hydrogen peroxide mix (APM) with diluted hydrofluoric acid (DHF)—are widely used in semiconductor labs. Then, the in-situ treatment is another key step to remove any native oxide or residual impurities on the Si fin surface. To choose an appropriate temperature that does not damage the Si fins’ shape is an important point.

**Figure 19:**
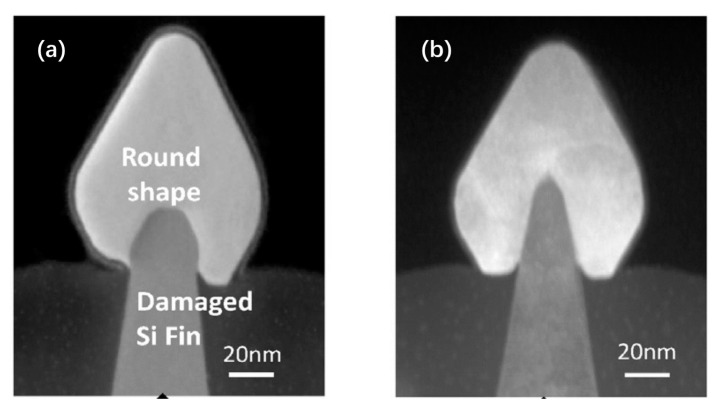
HRTEM cross-section micrographs of the Si-fins with different prebaking temperatures as follows: (**a**) at 825 °C and (**b**) 800 °C [104].

As an example, Figure 19a,b shows how the symmetric shape of Si fin can be damaged after pre-baking at 825 °C (Figure 19a) and 800 °C (Figure 19b). It shows that serious damage occurred to Si fins and as a result, the height of the fin is shrunk. However, the samples with pre-baking at 800 °C in Figure 19b demonstrate high quality Si fin surface after SiGe epitaxy. The symmetrical morphology of SiGe shows more strain induced [104].

### 5.2. SiGe, Ge Epitaxy Growth for Channel Region

Strain engineering in nano-scale transistors and devices is an effective method to deal with SCEs, involving high-mobility materials e.g., strained Si [105], SiGe [105,106], Ge [107], and GeSn [108] in the channel areas. The integration of high-mobility materials mainly has two trends:

(1) To grow the high-mobility materials as the channel first. In order to avoid strain relaxation in the channel, low thermal budget is demanded in device fabrication.
(2) To have Si channel for example in FinFET but later replace it with high-mobility materials (SiGe, Ge, and GeSn) with a high aspect ratio (AR). These channel materials can be deposited selectively in the transistor structures. In this way, the defects are formed and trap close to the fins’ sidewall (oxide) during epitaxial growth process. Finally, high-quality material is filled in the vertical direction inside the trench.

**Figure 20:**
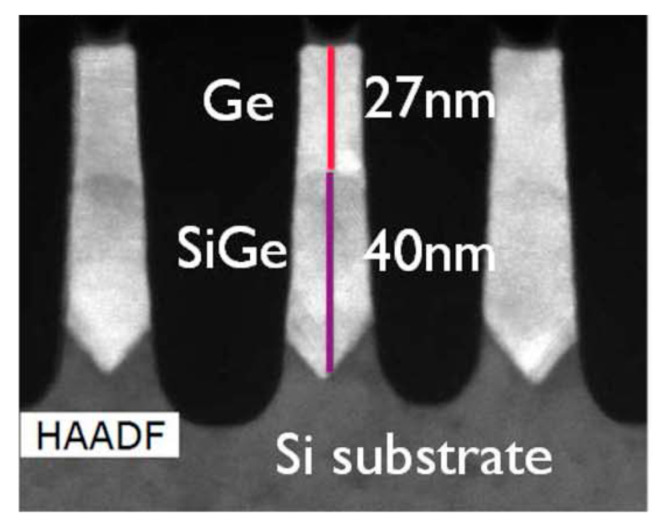
Cross-section TEM image of strained Ge-cap/ SRB Si_0.3_Ge_0.7_SEG grown inside oxide trenches of FinFET [110].

For the removing of Si Fin in the trenches, wet etch using diluted Tetramethylammonium hydroxide (TMAH) and the vapor etch using HCl inside the chemical vapor deposition (CVD) chamber are applied [109]. After filling the empty trenches, chemical mechanical polish (CMP) technique is performed to polish and planarize any existing overgrown film. Figure 20 shows the image of SiGe or Ge SEG in the channel area in a FinFET structure. The process starts to form a “V-shaped” Si recess surface and to grow the Si_0.3_Ge_0.7_ as strain relaxed buffer (SRB) layer [110].

### 5.3. SiGe/Si Mutil-Layers Epitaxy Growth for Gate-All-Around (GAA) Structure

For better control over a SCEs, a more aggressive design e.g., GAAFETs is proposed to be integrated beyond 5 nm node. For such transistors, SiGe or Ge is proposed to be grown as muti-layers and later selectively etched to form the channel region.

**Figure 21:**
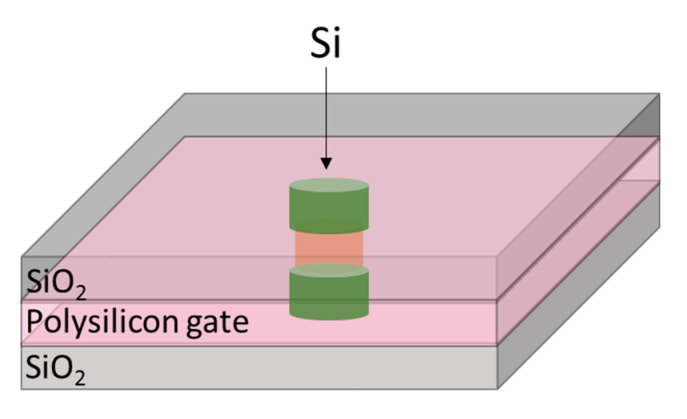
A vertical gate-all-around nanowire structure [111].

As shown in Figure 21, it is a vertical GAA structure that uses silicon nanowire as a channel to increase the channel width of the device and reduce leakage current [111], and effectively suppress SCEs.

**Figure 22:**
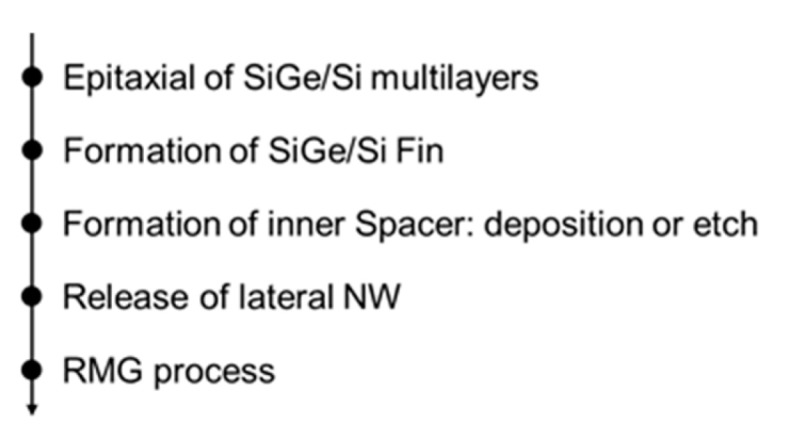
A manufacturing process of horizontal GAA-FETs (hGAA-FETs).

In GAAFET, the width of the channel is increased by using multiple Si nanowires. A fabrication process of hGAAFETs is shown in Figure 22. At first, a multilayer of SiGe/Si is grown on the substrate. After the multilayer growth, the fins are patterned by using spacer technology. Later, dummy gates and internal spacers, such as silicon-nitride, are defined. At this stage, selective etching of SiGe is performed with respect to the Si layer, and then a nitride layer is deposited and etched to define internal spacers. Finally, HfO_2_/TiN/W deposition can be used as metal gate for lateral GAA NWs [112].

However, the preparation process of nanowires faces some challenges, e.g., the control of nanowire sizes [113], and its ideal circular shape in cross section [114]. When hGAAFETs are processed, SiGe is used as a sacrificial layer and it has to be completely removed while retaining Si. With regard to vGAAFETs, precisely controlled etching of SiGe is critical for the device feature size.

SiGe/Si nanowires can be divided into vertical and horizontal nanowires. The vertical nanowire GAA requires more disruptive technological changes, while the horizontal one has less deviation from FinFET process, and the manufacturing is less difficult [112,115].

**Figure 23:**
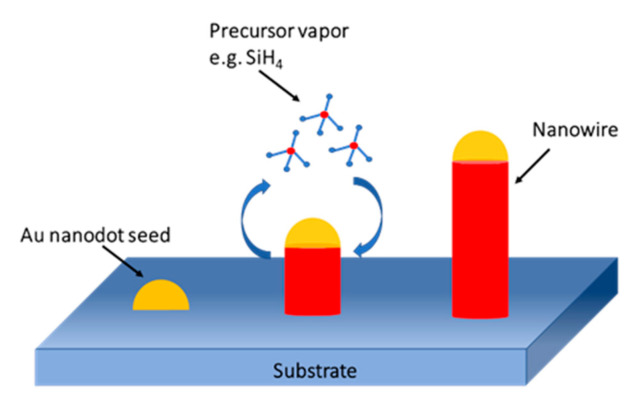
Schematic of VLS growing nanowires [117].

There are two methods to form NWs: Top-down and bottom-up ones. VLS is the most common growth method for bottom-up nanowire. The principle of this method has been described in Figure 23. The VLS metod applies a catalyst metal (typically Au), and chemical vapor deposition (CVD) technique is usually utilized for the NW growth [116]. The diameter of the NWs is determined by the size of the catalyst (catalytic seed) and related to the growth direction, and the length is determined by the flow rate and the time of the precursor vapor [117].

**Figure 24:**
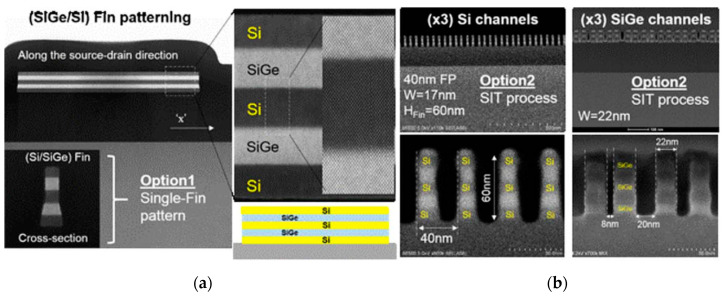
TEM images of post-etching of Si/SiGe multilayer fins in two directions (longitudinal and transverse cross-section). Two fin-patterning options have been designed: (**a**) Isolated and (**b**) dense FETs, which were carried out by sidewall pattern transfer technology [112].

The top-down preparation of SiGe nanowires mainly relies on SiGe’s selective isotropic etching process. The shape of the nanowire is determined by photolithography and etching. The top-down approach has many advantages for horizontal nanowire formation e.g., yield and availability of mature technology [112]. Wet etching is used to prepare SiGe\Si nanowires. The chemical liquid composition used to selectively etch SiGe is HNO_3_: BOE: H_2_O or H_2_O_2_: BOE: H_2_O. HNO_3_ and H_2_O_2_ react with Ge preferentially as strong oxidants, and the oxide layer reacts with BOE to remove SiGe. It is worth mentioning that the high concentration of HF is corrosive to Si and SiO_2_. Dry plasma etching can be used to prepare SiGe/Si nanowires as well. CF_4_/O_2_/He reaction system is used as the etching gas for SiGe. The high-energy particles of traditional plasma will cause damage to the lattice. The use of additional RF power can reduce ion-induced damage [116]. Compared with the traditional continuous etching, atomic layer etching (ALE) can achieve precise control of the etching size. The oxidant and the etching agent alternately cycle when ALE technique is used [113]. ALE can achieve atomic-scale reactions, and is a very promising method for etching small-scale patterns. ALE can be used in both wet etch and dry etch [113,118]. Figure 24 shows the etching of (SiGe/Si) multilayer fins.

## 6. Implantation and Advanced Doping Methods

Ion implantation has played an important role as a doping method in the fabrication of semiconductor devices for many years. Especially in the manufacture of CMOS devices, implantation offers a precise way to introduce expected species (B/P/As, etc.) into the wafers compared to traditional thermal diffusion. The quantity of both dopants and its distribution could be well controlled by this method. However, when the critical dimensions of MOSFETs evolved into nanoscale, 3D structures e.g., FinFETs or nano-wires, implanataion becomes more complicated. It happens when the architecture brings more requirements for the doping process to achieve highly activated, low damaged, and diffusion-controlled junctions.

### 6.1. Conformal Doping on 3D Structure Devices

One of the greatest challenges on 3D devices manufacturing is how to make a high aspect ratio doping within 3D structure. A large tilt is applied for implantation on FinFET initially, but later with the continuous requirement for increasing the packing density, the incident angles is restricted by the tight fin pitch due to the shadowing effect during implantation [119].

A novel doping technology called Plasma doping has been considered as an effective solution to achieve conformal doping distribution on 3D structures [119,120,121,122,123]. The technique is designed differently with traditional ion implanter where no species separate components in this system. The dopant gas is ionized in the chamber and the ions containing the dopant species are then extracted from the created plasma and can be accelerated to the substrate at varied angles. In this way, a more uniform doping profile could be obtained as demonstrated by K.Han et al. [124]. The amorphous layer created by plasma doping on a fin structure was more uniform than that done by beamline implant system.

Another alternative method for nano-scale doping is solid (or liquid) phased diffusion (SPD) technology. Dopant containing compounds are coated on the surface uniformly and then driven into the substrate via rapid thermal diffusion instead of implantation [125,126]. SPD could be carried out in several ways. For three-dimension structures, mono-layer-doping (MLD) is believed to be one of the most promising candidates [127,128,129]. The process starts from a self-assembled monolayer formation which is mainly composed of organic dopant. After the dopants are attached uniformly to the surface and catalyzed by a low temperature, the hydrosilylation reaction will be enabled to form covalent bonds between the molecules and silicon atoms of the substrates. After that, a rapid thermal treatment will break the molecules and drive the dopant atoms into the substrates. Until then, a capping layer is always needed to prevent the dopant from diffusing out into the atmosphere outside. The whole procedure is defect free, and the dose could be tuned by modulating the content of dopant impurities in the molecules. Moreover, the dose is affected by other factors due to the self-limiting surface reacting properties.

### 6.2. Defect Reduction

At present, implantation is still the leading candidate for doping, however it is well known that implantation for S/D regions and S/D extension will always bring damage to the substrate. This is especially observed when the energy or fluxion is high enough, a large number of defects will be created due to the cascade collisions between injecting ions and atoms of the target. In principle, the damage and most of these defects could be recovered later by a high temperature treatment like spike or millisecond annealing. However, there are always some deactivated clusters composed of interstitial or dopant atoms residue remained at the end of range area, which might degrade the resistance and lead to junction leakage.

Substrate temperature has to be chosen in order to effectively affect the damage generation [130,131]. It is known that implanted amorphous layer is essential for S/D engineering in CMOS to achieve highly activated shallow junction, but the disadvantage is the lattice damage and the created point defects. Fareen et al. has explained the physical mechanism of cryo-implantation. When the substrate temperature is decreased to −100 °C, implantation will be favorable to improve the amorphization by forming continuous thicker amorphous layer. In this way, the amount of Si interstitials is reduced and the formation of interstitial-boron (I-B) clusters is inhibited, which is the main mechanism of dopant deactivation and junction leakage [132,133].

Despite the fact that amorphization is greatly helpful in improving activation, it is not always expected in 3D devices. For 3D devices e.g., FinFET or NWs, the channel or extension implanatation should be in nanoscale and easily amorphized. Especially for the extension area, it is easily fully amorphized, which might cause permanent fin damage even after thermal annealing. This will result in poor parasitic resistance and higher junction leakage. To avoid this problem, Wood et al. tried to prevent amorphization generation by raising the substrate temperature in n-type S/D extension implantation. It has proved that hot implantation is effective to suppress the amorphization of the fin structure, and the junction could be improved by > 10 X, which means the damage is decreased greatly in this way [134].

### 6.3. Material Modification

Apart from doping process, material modification is another potential application of implantation in advanced devices. As reported, Ge, Si, or C are employed to inhibit the channeling or transient enhanced diffusion (TED) effect in ultra shallow junction (USJ) module [135,136]. Moreover, Ge or As is also proved to be favorable to decrease the contact resistance [137,138,139]. Since the structure of CMOS has evolved into three dimensions, titanium silicide has re-acted as standard contact schemes due to its better thermal stability and lower contact resistivity. 

Recently, pre-amorphous implantation (PAI) is reported to be effective to improve the performance of Ti silicide. Mao et al. [140] and Yu et al. [141] have demonstrated that when a low-energy pre-amorphous Ge implantation is applied on contact area, it will lead to great conductivity improvement, and an extremely low ρ of about 1.5 × 10^−9^ Ωcm could be achieved. The mechanism could be explained by the interfacial morphology promotion induced by PAI. The PAI process is helpful to enhance both the diffusion of Si atoms and bonding of Ti-Si [142].

## 7. ALD, HKMG and NC Materials

**Figure 25:**
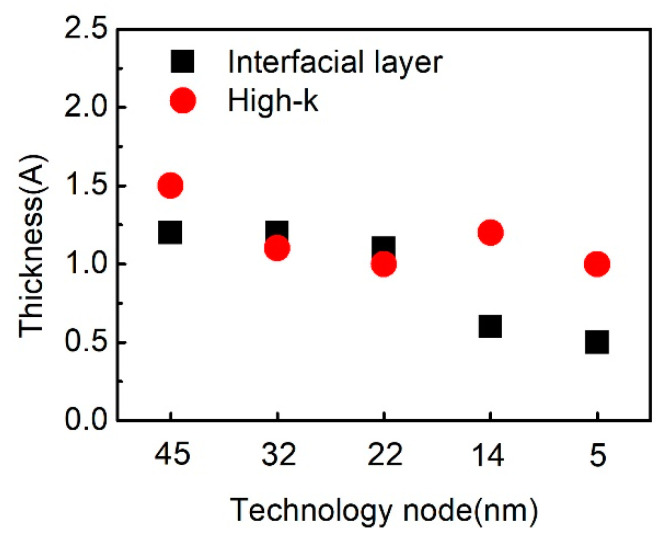
Interfacial layer and high-k dielectric thickness from 45 nm to 5 nm node.

High-k and metal gate have been introduced into CMOS technology to replace the traditional SiO_2_/poly-silicon gate electrode since 45 nm technology node. Along with the shrinking of the feature size, the type and thickness selection of the high-k and metal gate material are modified correspondingly. SiO_2_ and HfO_2_ are chose to be interfacial layer and high k dielectric from 45nm to 5nm technology node. Figure 25 shows the related thicknesses of these oxide layers. It can be seen that the interfacial layer decreases slightly from 1.2 nm to 1.1 nm when the technology node decreases from 45 nm to 22 nm, while the thickness of HfO_2_ reduces from 1.5 nm to 1.0 nm. Tri/Fin-FET structure is introduced instead of the planar structure. The thickness of SiO_2_ continuously decreases to 0.6nm while HfO_2_ thickness increases to 1.2 nm. SiO_2_ is the best passivation of Si surface, which can be used to make a device with high reliability. However, 0.6 nm is rather thin, which is only several layers of atoms and cannot be further decreased dramatically. So, SiO_2_ is estimated to be about 0.5nm and HfO_2_ is about 1.0 nm at 5 nm node.

**Figure 26:**
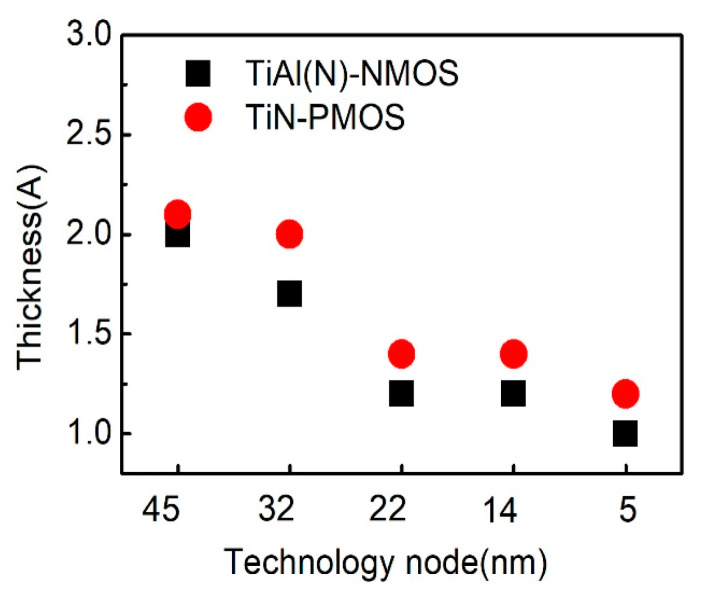
Work function metal thicknesses from 45 nm to 5 nm node.

For metal gate, the thickness of NMOS and PMOS work function metals range from the technology node is shown in Figure 26. TiAl(N) is used as NMOS work function metal and TiN as PMOS work function metal in both 45nm node and 32nm node device [143,144]. The thickness of TiAl(N) and TiN are ~2.0 nm and ~2.1 nm, respectively, in 45nm node, while they are ~1.7 nm and 2 nm in 32 nm node. The introduction of Tri-gate FinFET structure and gate-all-around (nanowire) structure improves the electrostatic gate control and the requirement of metal gate work function decreases. Thus, due to the miniturization of device and limited filling space, the metal gate thickness still needs to be decreased. The thickness of TiAl(N) and TiN are ~1.2 nm and ~1.4 nm, respectively, in both 22 nm node and 14 nm node. They are estimated to be about 1.0nm and 1.2nm for TiAl(N) and TiN, respectively, at 5 nm node.

The traditional way to deposit metal gate is physical vapor deposition (PVD), which can easily deposit a variety of metal gate materials and give much more metal gate selections. For the CMOS device of 45nm node and 32nm node, the aspect ratio of the gate stack is not so large that PVD can satisfy the requirements. However, when it comes to FinFET or nanowire structure, the aspect ratio is rather high, which can be more than 2:1 when the CD size is less than 20nm. Therefore, PVD cannot satisfactorily perform the filling of metal gate structure anymore. Atomic layer deposition (ALD) is the best solution, which has capability of excellent conformal step coverage [145]. HfO_2_ and P type metal can be easily deposited by ALD, but it is relatively difficult for N type workfunction metal deposition due to the limitation of precursor. Table 1 shows the typical related study results of different workfunction metals. Thermal ALD of TiN using TiCl_4_ and NH_3_ as precursors at 350 °C was demonstrated by Lujan et al. [146]. The effective workfunction was extracted to be 5.3eV, which is suitable for PMOS application. Jeon et al. fabricated stacked metal Ti_1−x_Al_x_N using TDMAT, TMA, and NH_3_/H_2_ plasma [147]. The effective work function was 5.04 eV. The same group could also grow TiC-TiN layers by using TDMAT and NH_3_/H_2_ plasma. The effective workfunction was decreased from 5.0 eV to 4.6 eV by increasing the growth temperature [148]. G. Cho et al. could grow TiC_x_N_y_ film using TDMAT and H_2_, NH_3_, and CH_4_/H_2_ plasma separately. It was found that the Ti-C and Ti-N bound have an important effect on the effective workfunction value. The lowest effective workfunction was estimated to 4.66 eV [149]. Most of the ALD N type metal is deposited by plasma enhanced (PE) ALD. PEALD of TaC_y_ using organic precursor and a carbon containing gas has an effective workfunction value of 4.77–4.54 eV [150]. Most of the other N type metals, TiC, TaCN, and WC_0.4_, etc., are also deposited by PEALD [151,152,153]. Compared with PEALD, thermal ALD has no plasma damage on the underlayer dielectric and could improve the device performance.

Chao et al. systematically studied thermal ALD TiAlC and TaAlC. The effective workfunction could be tuned and the lowest effective workfunction of 4.24 eV was obtained, which is suitable for NMOS application [154,155,156,157].

Currently, a major challenge for integrated circuits is the control of low power consumption. With the shrinking of the feature size of traditional transistor devices, the power consumption increases sharply. Low-power devices are the current research hotspots of integrated circuits. The sub-threshold swing of negative capacitance transistors is less than 60 mV/dec, which breaks through the sub-threshold swing limit of traditional transistor devices [159]. Therefore, V_dd_ and I_off_ can be effectively reduced and applied to low-power electronic devices. In the current reported data, the minimum value of the sub-threshold swing has reached 42 mV/dec [160] and negative capacitance transistors show excellent application prospects. The discovery of ferroelectricity of doped HfO_2_ materials provides tremendous potential for the negative capacitance transistors [161], due to the excellent compatibility with the nowadays COMS technology. However, the fatigue characteristics are still poor. In the near future, the research on negative capacitance transistors will continue to heat up. Finding suitable ferroelectric materials and realizing greater negative capacitance values are the focus and challenge of technological research and development.

## 8. ALD W for Nano-Transistors

Since Intel introduced high-k and metal gates (HKMG) to MOSFET, ALD technology has been applied to grow different related materials for nanoscale transistors. As an excellent electrode filler material, tungsten was first used in integrated circuits for contact hole filler tungsten bolt (W plug) in metal interconnects. After the appearance of HKMG, tungsten was also used as filler electrode material for metal grids. Recent studies about memory structures have shown that β- phase W can produce giant magnetoresistance (GMR) effects due to their large spin hall angles. This discovery has a high application prospect in third-generation magnetic memory [162]. Here, a brief of the current status and progress of ALD W films and their applications in semiconductor devices has been provided.

When depositing a mono-metallic in the atomic layer, the purpose of using a deoxidizer is to replace and remove the coordination group connected to the metal atom. The ALD W reaction belongs to the replacement reaction of fluoride (WF_6_) and deoxidizer (such as silane, ethylamine, etc.). At present, WF_6_ is commonly used as the precursor of W, and the B_2_H_6_ and SiH_4_ are used as replacement reductant precursors.

ALD growth process is very sensitive to the chemical properties of the surface. As a surface self-limiting chemisorption reaction, the higher the active sites on the surface exit, the greater the chemisorption probability is, and the better the surface nucleation. Hwanyeol Park et al. [163] simulated the process of ALD W deposited on TiN surface using B_2_H_6_ and WF_6_ as precursors by first-principles density functional theory (DFT). The precursor adsorption processes of ALD W on different TiN surfaces were investigated in detail. Three orientations and three positions of the B_2_H_6_ on three different Ti surfaces are considered, which are the TiN(111) of the Ti ending, TiN(001), and the TiN(111) of the N ending. The results show that the overall reaction energy between B_2_H_6_ and WF_6_ is lowest at N-terminated TiN (111). The study found that controlling the texture of TiN films is essential to improve subsequent W nucleation.

### 8.1. ALD W as Gate Filling Metal

**Figure 27:**
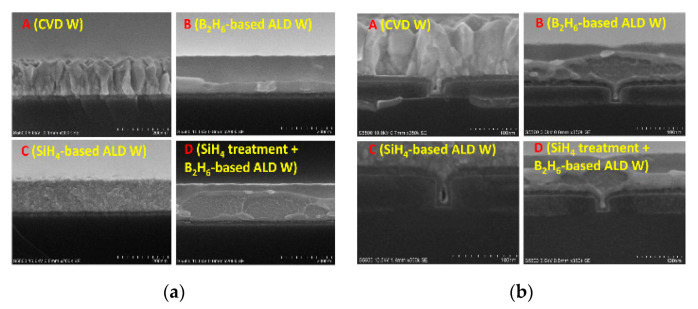
Cross-sectional SEM pictures of W films grown in different conditions: (**a**) On blank wafers and (**b**) trenches filling capacity [166].

ALD W can be used as gate filling metal for 22 nm and beyond nodes for CMOS technology [164,165]. Wang et al. has shown that the amorphous ALD W using B_2_H_6_ and WF_6_ shows lower growth rate, lower resistivity, and better gap-filling capability in gate trenches with high aspect ratio, in contrast to the polycrystalline ALD W using SiH_4_ and WF_6_ as displayed in Figure 27. Furthermore, it has been shown that the doping of boron (B) in ALD W does not affect the C–V and I–V characteristics of as-prepared capacitors. This indicates that the ALD W using B_2_H_6_ and WF_6_ is a good metal for gate filling, which can be widely used in nanoscale transistors [166]. It was found that, among filling metals, transistors filled by ALD W using SiH_4_ show higher drive capability and better control of SCEs.

**Figure 28:**
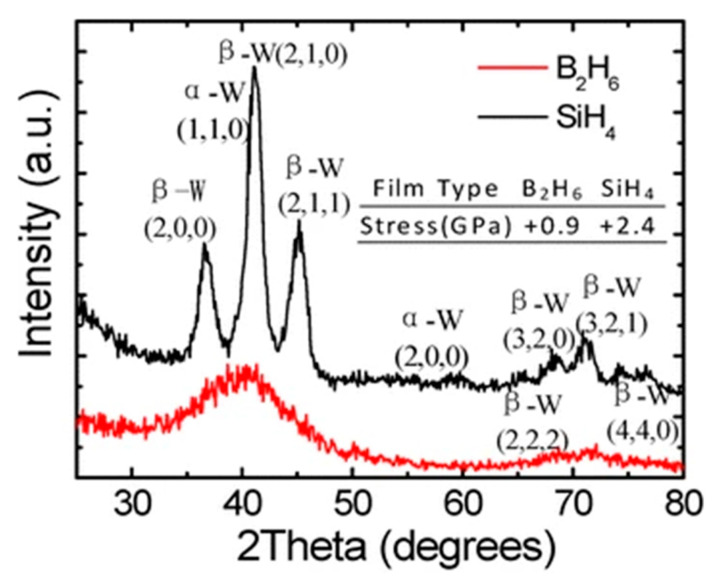
XRD patterns of ALD W grown by SiH_4_ and B_2_H_6_ precursors and the estimated stress [166].

Further investigations were performed by XRD to study the internal morphology of ALD W films. The results show that crystalline phases were detected in the ALD W films grown by SiH_4_ as precursor; meanwhile, the films deposited with B_2_H_6_ were amorphous as shown in Figure 28 [166].

### 8.2. Area-Selective ALD W

Area-selective ALD W is a bottom-up process and it solves the edge placement errors problems encountered in the process of top-down etching and other processes. By using the substrate-dependent nucleation of ALD processes, we can use the hydrogen to expand the substrate selectivity window during W atomic layer deposition [167]. Tungsten ALD is more energetically favorable on Si than on SiO_2_, but after several ALD cycles, the selectivity will be lost. Kalanyan et al. found that, during the WF_6_ dose step, adding H_2_ can help passivate SiO_2_ against W nucleation without affecting W growth on silicon. Surface characterization confirms that H_2_ can promote fluorine passivation of SiO_2_ through surface reactions with HF.

## 9. SiN_x_ Film and Strain Engineering

SiN_x_ is a kind of material that is widely used in CMOS-lines as gate dielectric, spacer, hardmask, and passivation layer. When time goes into the post-Moore age, with increase of transistors integration in a chip, more information will be transferred between chips optically, which enhances the demands of the interconnections. Different from electric current in metal wiring and electron transport, signal transmission in optical interconnection fundamentally comes from photons. It means that electron transmission by means of copper connection will not be the main action in the chip, but the signal is substituted with photons transmit within optical fiber or waveguide with low power consumption [168]. As the third-generation waveguide platform, SiN_x_ film shows much more superior performances than previous waveguide platforms including fibers and silicon waveguides. SiN_x_ film and its strain engineering play an important role in monolithic integration photonics and eletronics.

### 9.1. Different Properties of SiN_x_ Film

The properties of SiN_x_ film mainly depend on its chemical composition. Si/N ratio and chemical bonding variation will lead to the changes of mechanical and optical characteristics such as refractive index, stress, optical band gap, and Young’s modulus. In CMOS lines, low pressure chemical vapor deposition (LPCVD), plasma-enhanced chemical vapor deposition (PECVD), and ALD technologies are the normal processes. LPCVD technology could produce silicon nitride that is either highly strained and stoichiometric (refractive index is ~2.0@633 nm) or low stress and silicon rich (higher refractive index). PECVD-based silicon nitride has a composition that strongly depends on the deposition conditions, and can be silicon-rich, stoichiometric, and nitrogen-rich (lower refractive index). The best step coverage is the advantage of ALD-based silicon nitride. Classifying the SiN_x_ film by stress, it can be identified as tensile, compressive, and non-stress films. With these different properties, it can be applied in many technical areas.

#### 9.1.1. High Tensile Stress Contact Etch Stop Layer (CESL)

**Figure 29:**
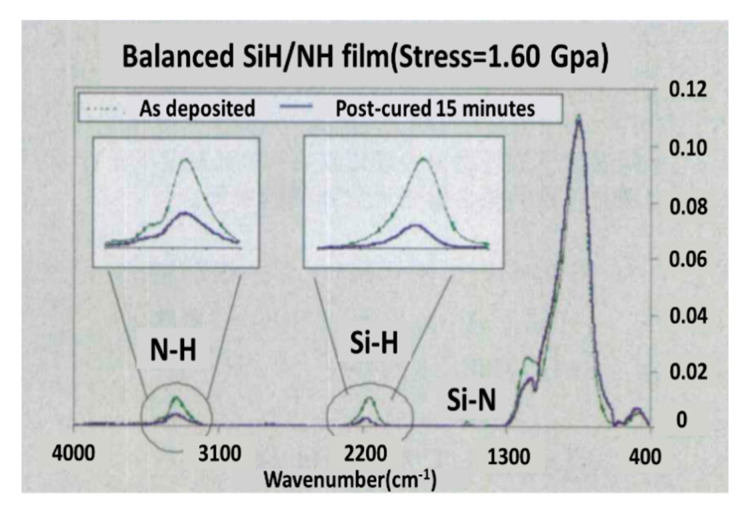
A spectrum shows the improvement of tensile stress using ultraviolet thermal process (UVTP) method [172].

In fabrication, tensile stress SiN_x_ film is usually manufactured by LPCVD or PECVD process. It is well known that LPCVD technique is usually used to create high-tensile-stress Si_3_N_4_ films up to 2Gpa. When we did not need to care about its high thermal budget, this deposition technique could be really a good choice. However, sometimes when the process flow reaches the backend of the line, high temperature would be incompatible, and the devices are practically destroyed [169]. PECVD technology is a highly efficient, mature, and low thermal budget process, which is the best choice in the backend of the line [170]. However, meanwhile, its low thermal budget brings the issue that the hydrogen in the silicon nitride film could not have been pushed out. This reduces the film tensile stress down to about 1Gpa [171]. In order to improve the tensile stress, plasma treatment and ultraviolet thermal process (UVTP) have been applied as shown in Figure 29. It is important to mention here that the UVTP treatment after films deposition could increase tensile stress in the films to 1.7 Gpa. During this method, Si-H, N-H bonding were broken to push out H gas through UV and thermal process. This ability made PECVD technology a good choice to meet the demands of both high tensile stress and low thermal budget.

#### 9.1.2. High Compressive Stress CESL

High compressive stress SiN_x_ film is also very important during the post-Moore age. Achieving high strain is hard for LPCVD technology while it is easier for PECVD technology. In order to produce high compressive stress film, there were high and low RF power sources coupled with diluted gas by PECVD. It has been found that the compressive stress has a high relationship with diluted gas species. The compressive value is about −1.2 Gpa when nitrogen was used as diluted gas [173]. The compressive value would be increased to about −2.3 Gpa when a mixture of argon and nitrogen was used as diluted gas. Furthermore, the compressive value could be increased to about −3.1Gpa with the changing of diluted gas to hydrogen and argon mixture. Here, the hydrogen is utilized to reduce energy loss during plasma bombardment. If further compressive value is desired, the elasticity modulus of the film must be improved by inducing carbon element. It is found that the compressive value could attain to −3.5Gpa when SiH_4_ is replaced with Trimethylsilane (TMS). The mechanism was that TMS contained carbon element, which impelled hydrogen to implement higher compressive value [174].

### 9.2. The Application of SiN_x_ Film in Post-Moore Age

#### 9.2.1. As Waveguide Transmitting Light in Photonics

Photonic integrated circuits have been based on several kinds of material platforms [175,176,177], and each platform has its own advantages and challenges. Today, mainstream silicon photonics products are built on silicon-on-insulator (SOI) wafers through traditional CMOS pilot line. Silicon nitride (Si_3_N_4_) photonics, a third integration platform, has characteristics and performance superior to the silicon-on-insulator (SOI) photonics and group III-V photonics platforms and is compatible with foundry-scale processes.

**Figure 30:**
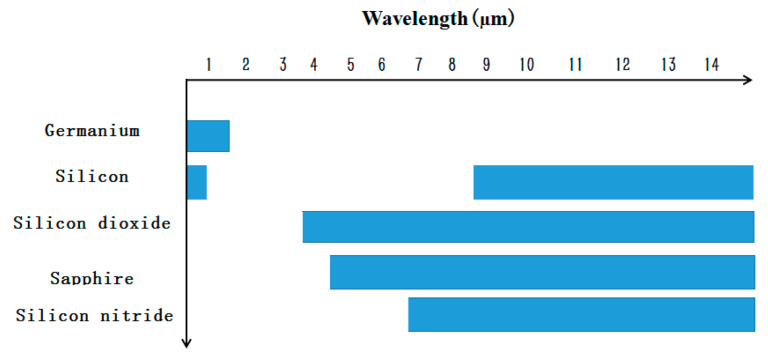
Light transparent window of the core materials for different waveguide and the white areas represent optical transparency meanwhile the blue areas signify high energy loss [178].

Figure 30 shows the light transparent windows of the different waveguide core materials. Compared with silicon and III-V platforms, silicon nitride core has a broad transparency range (0.4–2.35 µm), which takes it into the bio-science field where silicon core cannot. In addition to broad transmission wavelength, lower index contrast with silica compared to silicon, no suffering from two-photon absorption (TPA), and free carrier absorption are all silicon nitride’s superiority. 

#### 9.2.2. Strain Engineering in Modify Germanium Band Gap

It is well known that germanium is pseudo-direct bandgap material. A transition from indirect to direct band gap has been predicted for tensile-strained germanium since the energy position of the conduction Γ valley versus strain decreases more rapidly than the band-edge L valley [179,180]. Many methods for increasing the tensile stress in Ge have been explored, including strained Ge growth, thermally induced strains, external stresses, stressor layers, and strain redistribution. As a stressor layer, SiN_x_ film is a very attractive possibility to change germanium band structure because of its compatibility with a CMOS processing and the flexibility to control the stress transfer and achieve high tensile or compressive strain [181,182,183,184]. In 2013, Centre national de la recherche scientifique (CNRS) demonstrated that when a strong tensile strain silicon nitride stressor covered germanium microdisks growing on GaAs substrate, up to 1% biaxial strain resulted in direct band gap emission red-shifting from 1550 nm up to 2000 nm under the room temperature [183]. In 2015, their publication said that when all-around tensile stress of silicon nitride was brought up to 1.45%, it would result in red-shift toward 2100 nm wavelength.

SiN_x_ film is also a kind of flexible material whose properties and characteristics are altered as process conditions changed. The unique ability makes it widely used in the post-Moore era especially in the field of silicon-based photonic integrated circuit. 

#### 9.2.3. The Application of SiN_x_ Stress Engineering in IV MOSFET

There are various advanced stress technologies to enhance the carrier mobility in the channel including shallow trench isolation (STI), nitride CESL, embedded SiGe, or SiC in source/drain [185]. Among all these feasible stress inducing approaches, the intrinsic stress of thin SiN_x_ film by a CESL has become an important technique that receives much attention because it is easy to execute in nanoscale semiconductor devices.

**Figure 31:**
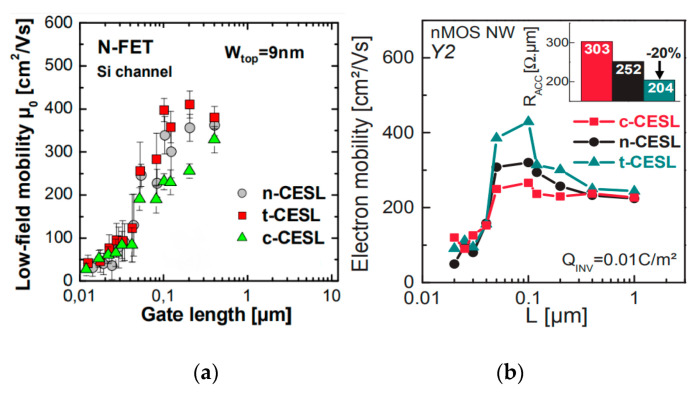
NMOSFET electron mobility vs. contact etch stop layer (CESL) technology, (**a**) in 9 nm n-FET NW. A better mobility is observed under tensile CESL [187] and (**b**) Electron mobility vs. L for different CESL in nanowires. Inset: extracted access resistance [188].

The effect of CESL in IV MOSFETs with Si and Ge and GeSi channel material have been reported in many publications. Recently, more and more articles pay attention on nanoscale Ge or GeSi MOSFET. Both the experimental and simulation work presented the stress-induced mobility gain of CESL as strongly dependent on the layout arrangement of devices, particularly in situations with narrow widths [186]. P. Nguyen et al. has demonstrated induced CESL in Ω-gate CMOS nanowires with p-FETs and n-FETs down to W = 15~20nm gate length [187,188]. As shown in Figure 31a,b, tensile CESL is the best option to improve n-FET performance. c-CESL, n-CESL, and t-CESL represent compressive-CESL, neutral-CESL, and tensile-CESL, respectively.

## 10. Advanced Etching for Nano-Transistor Structures

**Figure 32:**
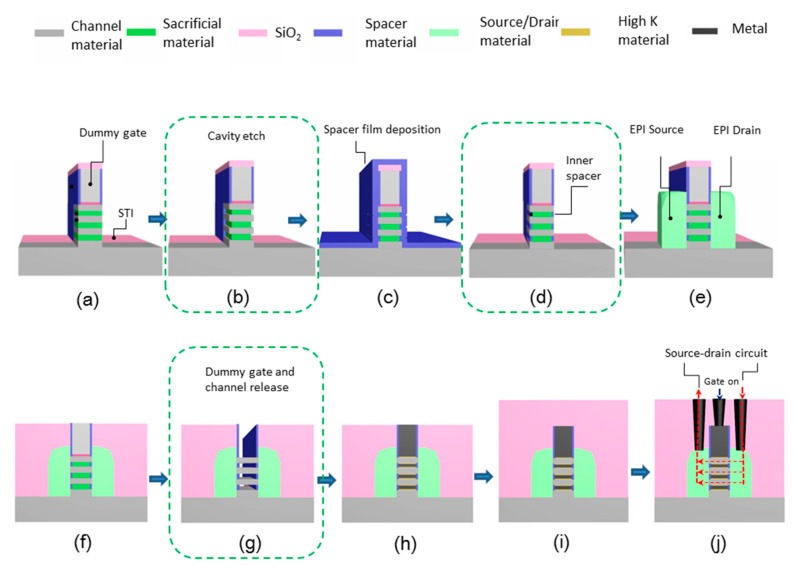
Process flow and critical etching steps for manufacturing lateral nanowire GAA device: (**a**) Source/drain Fin recess for opening active area, (**b**) cavity etching for defining the growth position and size of the inner spacer, (**c**) inner spacer film deposition, (**d**) controlled etching of spacer film and formation of inner spacer, (**e**) source/drain epitaxial growth, (**f**) dielectric deposition and planarization, (**g**) dummy gate removal and nanowires formation, (**h**) filling and planarization of high-K metal gates, (i) interlayer dielectric deposition, and (**j**) metal contact plug and current direction when device is on on-state [195].

**Figure 33:**
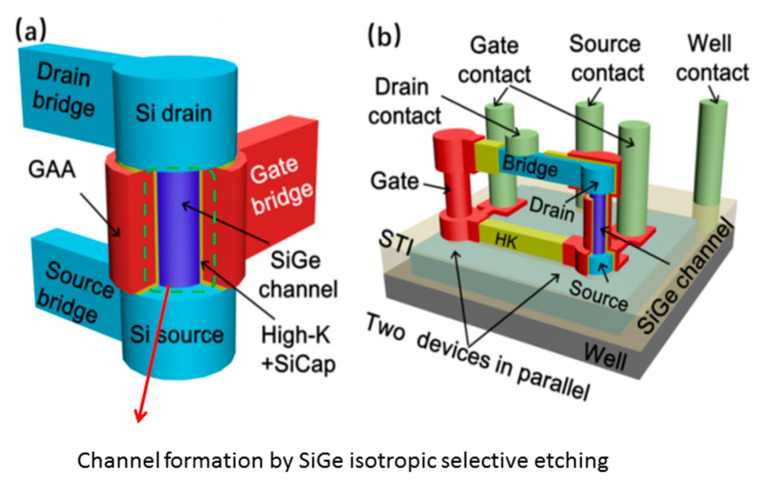
Schematic of vertical nanowire GAA transistor: (**a**) Structural design for a single device, and (**b**) test structure with two devices connected in parallel via a local interconnect bridge [46].

As CMOS technology enters the 3 nm node, GAA nanosheet/nanowire becomes the most powerful competitor to replace FinFET technology because of its excellent control of SCEs [189,190]. As it was mentioned above, GAA devices mainly have two forms, horizontal [191,192] and vertical [46,193,194], and selective etching plays a very important role in these manufacturing processes. For the preparation of horizontal nanowires shown in Figure 32, there are three main steps that require precise selective etching to prepare inner spacers and release dummy gates and nanowire channels. For vertical nanowire devices (see Figure 33), selective etching is used to precisely control the diameters of the channels.

### 10.1. High Selective Etching for Channel Full Release

Removal of the sacrificial layer to obtain nanowires or nanosheets is a very critical process step (as shown in Figure 32g). This step requires a high etch selectivity when the nanowire channel is released. This is achieved through the selective etching of sacrificial layer to the channel material. The channel material is generally Si, with SiGe sacrificial layer. Conversely, the channel material may be Ge or SiGe, and the sacrificial material is SiGe or Si. Therefore, SiGe selective etching is required to obtain silicon channels, or silicon selective etching is required to obtain SiGe channels.

**Figure 34:**
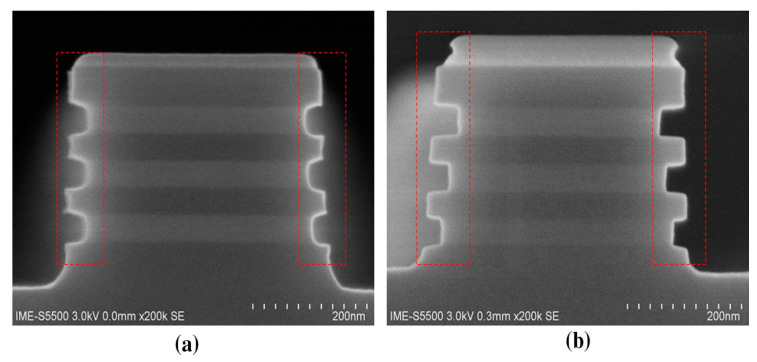
The SEM images of cross section comparing the profile between wet etching and inductively coupled plasma (ICP) dry etching: (**a**) Wet etching SiGe with 6% HF/30% H_2_O_2/_99.8% CH_3_COOH = 1:2:4 etching 8 min, and (**b**) ICP dry etching SiGe with CF_4_ /O_2_ /He = 4:1:5 [207].

**Figure 35:**
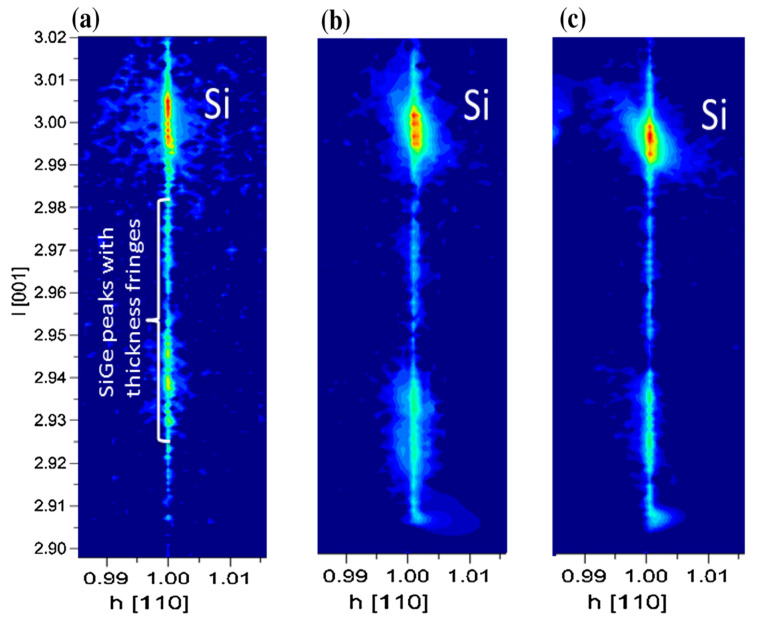
High-reosultion reciprocal lattice maps (HRRLMs) in the vicinity of the asymmetric (113) Bragg reflection acquired on SiGe/SiMLS: (**a**) Unprocessed structure; (**b**) after vertical anisotropic etch and 100:1 DHF wet clean, and (**c**) lateral isotropic SiGe selectivite by using ICP dry etching [207].

For SiGe selectivity etching, alkali and acid containing oxidant are commonly used chemical reagents, such as H_2_O_2_/NH_4_OH [196] and H_2_O_2_/HF/CH_3_COOH [197,198]. The outcome of this etching method easily leads to structural collapse due to the solution capillary effect, especially for structures with high aspect ratio and small size. One way to overcome the above limitation of the liquid reaction is to change the process to selective etching of SiGe by vapor phase HCl [199]. The gaseous HCl method may offer a high selectivity to Si in a suitable high temperature range, and there is no collapse problem after etching a small size structure with a high aspect ratio. One drawback with this method is that high reaction temperature will cause potential risks of dopant diffusion and stress relaxation [200,201]. Remote plasma selective etching of SiGe is a technique with higher precision than the above pure chemical etching, and it is a process under room temperature without additional thermal effects on the material. The etching gases are mainly CF_4_ [202], CF_4_/O_2_/N_2/_CH_2_F_2_ [203,204], and NF_3_/Ar/NO [205]. Conventional plasma etching is usually used for anisotropic etching [206]. However, using CF_4_/O_2/_He in traditional inductively coupled plasma (ICP) for SiGe selective etching has achieved a good etching effect. The etching morphology has advantages over wet etching as shown in Figure 34 and damaged material can be removed and the SiGe layer is preserved as shown in high-reosultion reciprocal lattice maps (HRRLMs) in Figure 35 [207]. The position of SiGe peak in all maps is aligned to the Si peak imdicating neglible strain relaxation after initial vertical etch and in consequence of lateral etch steps.

Selective etching of Si leaves SiGe nanowires to obtain high mobility channels, therefore research in this area has remarkably boomed. Wet alkaline solutions such as NH_4_OH [208] and TMAH [209,210] have a high selectivity in silicon etching to SiGe, but all have obvious crystal orientation, which is due to the etching rate Si (100) > Si (110) > Si (111). Liu et al. reported that ACT^R^ 210 and ACT ^R^ 301 reduced the crystal orientation behavior of etching and improved the selectivity ratio of Si (111)/SiGe etch to 13: 1 [211]. In order to overcome the wet crystal orientation anisotropy and liquid capillary effect, dry etching is preferred. The dry etch mainly includes SF_6_/CF_4_/H_2_ (selectivity Si to Si_0.5_Ge_0.5_ is about 10: 1) [212], CF_4_/H_2_/Ar (selectivity Si to Si_0.5_Ge_0.5_ is about 30: 1) [25,213] and CF_4_/N_2_/O_2_ (selectivity Si to Si_0.5_Ge_0.5_ is about 10: 1) [214]. Both dry and wet etching methods overcome the crystal anisotropy issue, but the etching selection ratio still needs to be improved.

### 10.2. Precise Selectivity Etching for Channel Partial Release

**Figure 36:**
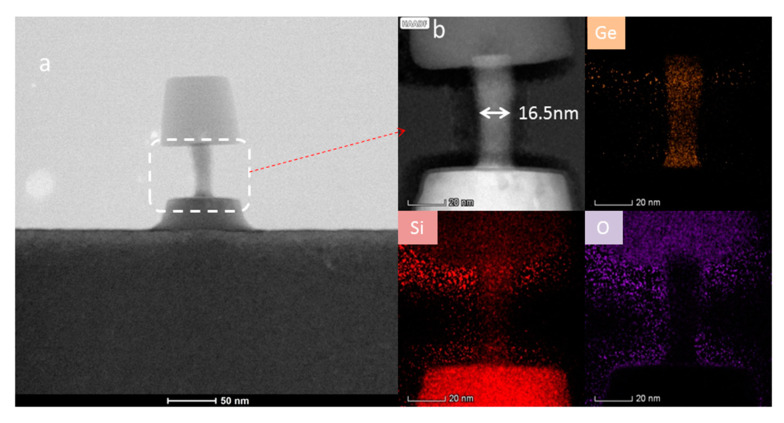
TEM and EDX mapping: (**a**) TEM picture of all structures; (**b**) HRTEM and EDS mapping of near isotropic etching region [113].

An accurate release of the channel layer is a critical step for nano-transistors. For horizontal nanowires, the precision of selective etching will affect the effective gate length, and for vertical nanowires it will affect the channel diameter [194]. The above-mentioned etching techniques offer good etching selectivity, but the etching accuracy is not high enough to meet the demand of cavity etching in the inner spacer and the precision control of the diameter of the vertical nanowire preparation. Therefore, it is necessary to develop new etching methods e.g., digital atomic layer etching (ALE) or quasi-atomic layer etching (QALE) techniques [215,216,217]. Pargon et al. proposed digital etching with alternating O_2_/He gas oxidation and NF_3_/NH_3/_O_2_ etching to obtain a high SiGe etching selectivity to silicon (60: 1), but the accuracy is not high enough (about 19 nm/cycle) [218]. Yin et al. reported a digital etching method using H_2_O_2_ oxidation and buffered oxide etchants (BOE) to etch oxide alternately. Because both steps have self-limiting features, the etching accuracy reaches 0.5 nm/cycle [59]. Li et al. proposed a digital etching method of alternating O_2_ plasma self-limiting oxidation and CF_4_/C_4_F_8_ plasma limited selective etching of SiGe oxide layer, with an accuracy of 0.3 nm/cycle. The etching results are shown in Figure 36 [113].

### 10.3. Precise Selective Etching of Dielectrics and Other Materials

**Figure 37:**
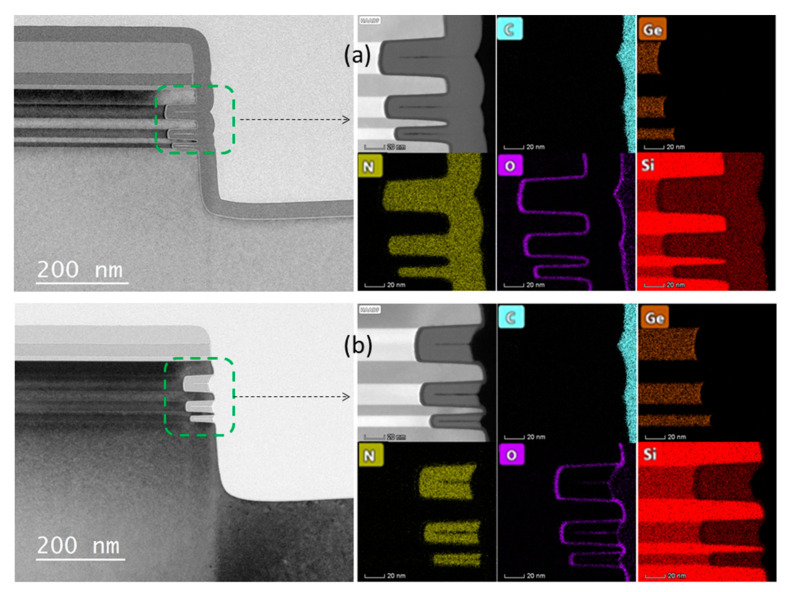
TEM and EDS micrographs: (**a**) LPCVDSilicon nitride inner spacer deposition; (**b**) inner spacer after etching under optimal conditions [195].

The spacer between the source-drain and the gate can both prevent leakage and reduce parasitic capacitance. For GAA nanowire devices, the inner spacer is a necessary structure, but its thickness must be precise. The thin thicknesses will increase the leakage and parasitic capacitance. The thicker thicknesses will increase the resistance between S/D when the device is turned on [191,192]. Inner spacers have greater challenges than conventional spacers where a higher etching selection ratio and etching accuracy are required [195]. In addition to the good dielectric properties, the corrosion resistance of the inner wall material is also very important, because the sidewall material needs to undergo nanowire release in the nanowire preparation process, and it needs to be resistant to erosion in the arounding process. Therefore, silicon nitride is still a more ideal material [219]. The selection ratio of traditional etching methods cannot meet the requirements of inner spacer [220]. The selectivity ratio of remote plasma method is high enough, but it is not suitable for etching of inner spacer because of its completely isotropic etching characteristics and low etching accuracy [221]. The emerging digital etching or quasi-atomic layer etching has the potential to be applied to the inner spacer because of its high etching selectivity and etching accuracy [222,223]. However, because of its relatively complicated hardware system and low production capacity, it hampers its rapid application in industry. Li et al. studied the conformal deposition of inner spacer using LPCVD, and the precise dry etching using an innovative gas mixture of CH_2_F_2_/CH_4_/O_2_/Ar. The structures are shown in Figure 37 [195].

In addition to the above-mentioned SiN materials, high selectivity for precise etching of SiO_2_ [224], and new channel materials GaN [225], InGaAs [226], and MoS_2_ [227] are all worthy of attention.

## 11. WET Cleaning

In the nano-scale transistors, the replacement metal gate (RMG) was introduced by using dummy gate (poly silicon) and dummy gate oxide (silicon dioxide) to go through all the high-temperature annealing processes [228]. This gate oxide is eventually removed in a wet-etch process by using quaternary ammonium hydroxide (QAH) and hydrogen fluoride acid-based solutions [229,230,231], respectively. There are many merits for RMG. First, it avoids crystallizations of the high-k dielectric during the rapid thermal annealing (RTA) process for dopants activation, which may increase leakage current of the gates [232]. Second, it avoids the chemical reactions between the metal gate and the high-k in RTA processes [232]. Also, it prevents the boron from diffusing into high-k because the diffusion into hafnium oxide (HfO_2_) occurs much slower than SiO_2_ [232,233]. The RMG process still will work well for the 7 nm and 5 nm technology node with SiGe nanowires through selectively removing the Si sacrificial layer from the SiGe channel material [234]. Meanwhile, for technology nodes 3 nm and beyond, the sacrificial material is SiGe and will be selectively removed from Ge channel [235,236,237].

In spite of the evolution of wet etching reagent, the drying process after the wet etching or cleaning remains to be a great issue for the high aspect ratio (HAR) 3D CMOS structures. In general, the wet processes use aqueous solutions, which use water as the solvent, and the final rinses processes use deionized water (DIW) or ultrapure water (UPW) to clean away the chemicals from wafer surfaces. However, it is well known that some defects can happen due to the surface tension of water. Normally, during the drying process, the high capillary force of water could pull nearby structures to form permanent imperfections, which are called pattern collapse [238], or stiction [239] in terms of micro electro-mechanical systems (MEMS) field. Historically, in order to minimize the surface tension, liquid isopropanol (IPA) has been used [240,241,242], but it eventually becomes ineffective as pattern spacing decreases and aspect ratio increases.

**Figure 38:**
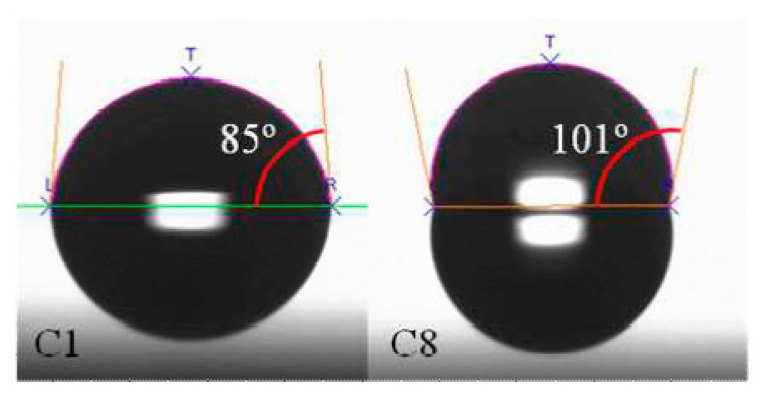
Contact angle test of C1 and C8 [243].

In case of the stiction emergence, the adhesive force (caused by the capillary force), which pulls the neighboring HAR structures, overwhelms the elastic force, and the structures’ integrity becomes safe. In order to minimize the capillary forces, besides using lower surface tension liquids to replace the water with isopropyl alcohol (IPA), reducing the surface energy is also a possible solution. In the field of microfabrication, the hydroxyl groups are considered highly reactive and are often seen from the silicon oxide surfaces after SC1 or ozonated water treatment. Therefore, replacing the hydroxyl groups with inactive molecules like silylation agents is a good solution to minimize the stiction [243]. In ref.243, two kinds of straight-chain alkyl group were compared by means of drying HAR structure evaluation. The carbon number of the straight-chain alkyl group in the agents is 1 and 8, so they are named C1 and C8, respectively [243]. The contact angle of C1(85°) is lower than that of C8 (101°) as shown in Figure 38, which means longer straight-chain alkyl groups enable a more highly hydrophobic surface [243].

**Figure 39:**
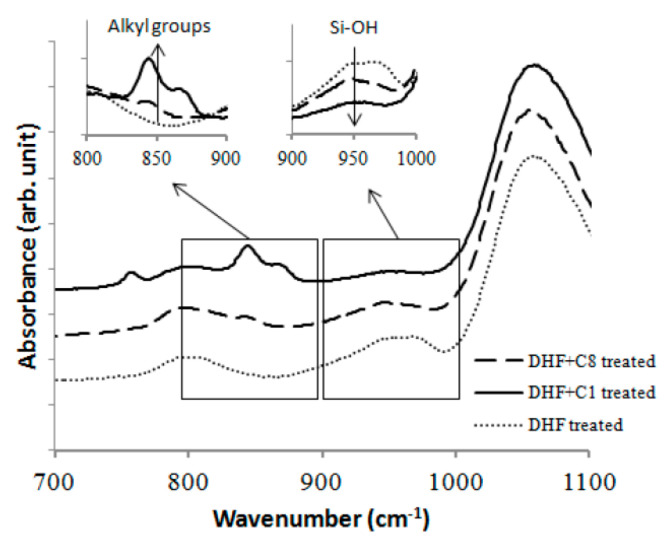
ATR-IR spectrum measured from silicon oxide powder treated with C1 or C8 shows that C1 has better result because of higher hydrocarbon groups around 750 to 900 cm^−1^, while lower hydroxyl group at 960 cm^−1^ [243].

**Figure 40:**
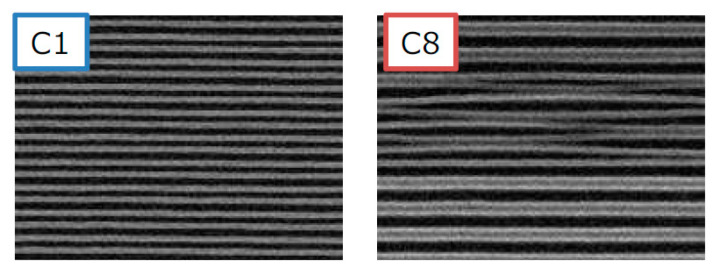
Top-view SEM image of samples treated with C1 and C8 after drying [243].

However, silicon oxide powder samples treated with the C1 and C8 showed different results. Attenuated total reflection–infrared spectroscopy (ATR-FTIR) measurements results showed that C1 treatment sufficiently eliminates the hydroxyl groups, as shown in Figure 39. On the other hand, it is expected that the C8 reaction was suppressed by its steric hindrance, therefore the existing hydroxyl groups could not be sufficiently substituted with alkyl groups [243]. The drying observation of the HAR structures with aspect ratio of 13.3 using the C1 and C8 confirmed that it is not important to obtain high water repellency, but it is very important to obtain lower surface free energy, as shown in Figure 40 [243]. The alkyl group surface can be easily removed by oxidative or reductive plasma strip by means of N_2_O or N_2_/H_2_ plasmas [244]. Farid et al. demonstrated the STI structures with 9 nm CD, 25 nm pitch, and 160 nm fin height [244].

## 12. Metal Materials Interconnect

As semiconductor technology keeps evolving along the Moore’s Law, the critical dimension (CD) of the BEOL circuits must continue shrinking as well [1,245]. Therefore, interconnects have become one of the factors that greatly affect the performance and reliability of semiconductor devices. Over the past 20 years, dual damascene copper replaced the subtractive etch of aluminum for BEOL interconnect fabrication [2,246]. However, scaling can significantly reduce the electromigration (EM) lifetime for Cu interconnects, raising serious reliability concern for advanced technology nodes. Some improvements have been developed to improve EM and to extend the technology node by using CoWP metal cap [247,248] and Mn alloying [249,250,251].

However, in the 7 nm technology node and beyond, copper interconnection in device scaling is facing difficulty in resolving the trade-off between line resistance (Line R) and manufacturability/reliability [252]. Because of this limitation, Cu is facing challenges, and alternative conductors and integration technologies have been investigated to overcome the issue.

Scaling traditional PVD TaN barriers often lead to inconsistent film coverage on ultra-low K dielectrics. ALD TaN could provide a solution due to the nearly 100% conformality even in HAR structures, hence reducing “pinch off” and Cu void formation. However, ALD TaN films contain higher impurity content than PVD TaN films resulting in higher via R and poor interface quality. Moreover, ALD barriers have not delivered on their promise as PVD Ta flash and/or PVD Cu seed layers were still needed to provide a good barrier-Cu interface [253]. Some integration schemes of modified ALD TaN Barrier with Co/Ru liner are reported to reduce line/via resistance and improve interface quality and reliability as required in advanced technology nodes. Zhiyuan Wu et al. reported a novel approach to enable thin (≤15Å) ALD-based TaN barriers [254], Co liner is used for copper electroplating. The use of a post-ALD treatment in a PVD chamber resulted in ALD films with resistivity, density, and Ta/N ratio similar to industry-standard PVD TaN. This approach enables conformal Cu barrier without reliability degradation compared to PVD TaN and overcomes the shadowing effect of the traditional PVD approach improving the metal-fill process window and promotes lower via resistance through barrier thickness reduction. Bhosale et al. [255] reported scaling of the PVD component in the ALD + PVD process followed by post-treatment methods such as PPT (physical-vapor-deposition post treatment) and IPT (in-situ plasma treatment). The PPT and IPT processes produce an interface more suitable for liner (CVD Co/Ru) and PVD Cu seed. These results show the improvement by Cu gap-fill as well as the decreased via resistance (R) by sputtering TaN at bottom of the via. IPT processes densify the ALD films and improve EM performance. A promising integration scheme of 20 Å ALD + IPT TaN/20Å Ru/DCR was developed with 20X improvement in electromigration. Lanzillo et al. [256] reported the line resistance, via resistance, electromigration, and time-dependent dielectric breakdown (TDDB) for Cu interconnects with TaN/Co barrier/liner metallization. This work varies the Co liner thickness at 36nm BEOL pitch. The outcome of this study indicates that If the PVD treatment is followed by deposition of a thin ALD TaN, the Co layer thickness can be scaled down to 10Å without any penalty in either EM or TDDB. The combined PVD/ALD process with 10Å Co enables a 14% reduction in RC delay relative to control split (20Å PVD TaN + 30Å CVD Co). Motoyama et al. [257] reported sidewall voids observed in case of Co liner, whereas void-free Cu fill was achieved with Ru liner in 30 nm pitch interconnects. The results indicate that Ru liner is superior to Co liner in terms of Cu fill at these nanoscale dimensions. The combination of 1 nm PVD Ta/1 nm ALD TaN/ 1.5 nm CVD Ru could achieve void-free Cu fill in 30 nm pitch interconnects and comparable performance to PVD TaN for both EM and TDDB. Ru CMP issues (Cu recess and trench height variability) were mitigated significantly by scaling down the Ru liner thickness with PVD Ta/ALD TaN barrier stacks.

For 7 nm technology with via and trench sizes below 15 nm, it is highly desirable to prefill the via with Co, or other low resistance metals, to reduce via resistance and improve the Cu fill margin [258]. The via prefill is beneficial for the Cu damascene process for several reasons. First, a barrierless via prefill material moves the Cu trench barrier location from the bottom to the top of the via, thereby reducing the resistance by increasing its cross-sectional area in tapered via. Second, in moving this trench barrier, the damascene metallization dimensions are more relaxed, which promotes a void-free Cu trench fill. Third, the required step coverage for the Cu barrier and liner in the trench at the via top location will be reached more easily with thinner films compared to the via bottom location. For these reasons, the via prefill concept could be one of the most promising innovations for the damascene process in the coming nodes. Zheng et al. [258] and Marleen et al. [259] respectively introduced Co via prefill concept to achieve void-free and bottom-up fill of metal in advanced interconnects. Zheng et al. reported a highly selective CVD Co deposition on Cu to fill a 45nm diameter 3:1 aspect ratio via in a Cu dual damascene structure [258], and they achieved void-free Co fill of the via.

Several reports have also demonstrated the feasibility of the via prefill concept using the electroless deposition (ELD) technique Co as material to pre-fill vias [259,260,261]. A main benefit of having Co via is the reduction of the via resistance. As the via CD shrinks, the electroless Co via shows a larger relative resistance reduction from the conventional PVD-ECP via. For example, at about 40 nm via CD, the via resistance reduction is ~ 30% [260]. By using hybrid Cu metallization with Co via-prefill, the resistance of a 12 nm half-pitch via can be lowered by up to 42% in 87° tapered vias and up to 52% in chamfered vias [262]. Therefore, selective Co process for contact and via prefill has the potential to enable future scaling of advanced technology node.

As a promising candidate, Ru has received great attention because of its low bulk resistivity, superior electromigration reliability, and the prospect of barrier-less process [263,264,265]. Currently, the damascene implementation of ruthenium lines is hampered by the availability of optimized CMP. A semi-damascene integration approach is a proposed solution for multilevel Ru interconnect [266,267]. Danny Wan et al. reported that Ru films were patterned using EUV single exposure and a subtractive etching step to generate lines with CD down to 10.5 nm. The key advantages are that the process can be barrierless, grain size can be tuned, there is no requirement for metal CMP, and eliminates the need for plasma processing of low-k trenches. This approach has excellent process control, stability, and results in very high line yield. 

Resistance contribution within Ru interconnects are evaluated using first-principles density functional theory, which is based on transport calculations using the non-equilibrium Green’s function. Results show that the defects and impurities can change the Ru resistivity by an order of magnitude. Seong Jun Yoon et al. [268] reported that maximizing the grain sizes in Ru wires can effectively lower the total wire resistivity. With the successful suppression of the grain-boundary scattering effect, a reduction in total wire resistivity of more than 30% was achieved. The result suggests by that enlarging the grain sizes of Ru and combining it with a subtractive patterning process [266,267], the physical limitation of nano-grain structures in damascene Ru can be solved.

ALD Ru was studied as an option for barrierless metallization for the future interconnects [264]. Ru shows regular nucleation on SiO_2_ without any growth inhibition, and the adhesion was significantly increased to 7.0 ± 2.3 Jm^2^ by applying an ALD TiN adhesion promoting layer with a thickness as low as 0.25 nm. The Ru lines with widths of about 10 nm show excellent EM behavior on single damascene test vehicle. Time-dependent dielectric breakdown measurements revealed negligible Ru ion drift into dense low-k dielectrics (with k ~3.0 up to 200 °C), demonstrating that Ru has the potential to be used as a barrierless metallization as future interconnect solution.

PECVD of Co was evaluated to fill DD structures as an interconnect wiring metal alternative to Cu [269]. Void-free gap fill of damascene structures down to 10 nm CD was demonstrated using just 1 nm ALD TiN liner. A CMP process without Co residues or corrosion has been also demonstrated. Also, 22 nm half-pitch Co lines with 1 nm ALD TiN liner in porous ULK meet the 10-year lifetime TDDB reliability requirements [269]. The experimental data indicate that the Co EM performance with 1 nm ALD TiN liner can be better than that of Cu. CVD cobalt has been considered, but it appears too costly and time consuming for high volume manufacturing. Electro chemical deposition (ECD) of Co is deemed the most promising candidate. Intel has shown a new metallization solution to meet the reliability challenges of technology scaling [270]. For this process, at trench contact, electroplating of Co occurs on a CVD TiN barrier/adhesion and a CVD Co seed layer. EM failure time is observed to be at least four orders of magnitude higher for Co fill interconnects compared to Cu alloy. Moreover, superior intrinsic TDDB and stress induced voiding reliability was also demonstrated for Co low-k interconnects.

**Figure 41:**
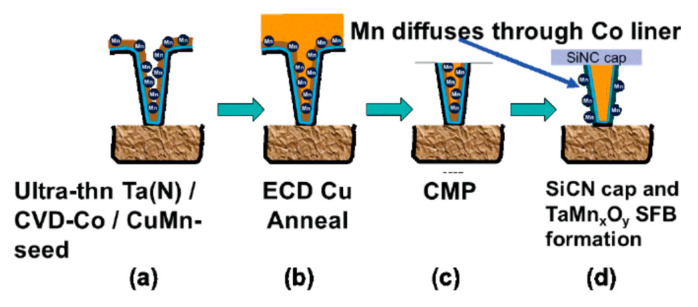
Through-Co self-forming barrier (tCoSFB) structure and process flow: (**a**) Ultra-thin Ta(N)/CVD-Co/ high-Mn% PVD-Cu(Mn) seed stack layer deposition; (**b**) Cu electroplating and anneal; (**c**) Cu CMP; (**d**) PECVD SiCN(H) dielectric cap and TaMnxOy SFB formation [277].

A self-forming barrier uses a Cu-X alloy-based seed layer to form the barrier layer by post metallization annealing. Among the tested alloying elements are V, Al, and Mn [249,250,251,271,272]. Mn-based self-formed barrier (SFB) is an attractive alternative technique from RC performance point of view. Previous studies based on PVD Cu-Mn alloy seed and CVD depositions of Mn from various precursors have been demonstrated alternative route for SFB formation [273,274,275,276]. Nogami et al. from IBM [252] showed Line R cross-over point to alternative metals depends on barrier/wetting layer thickness. When the barrier/wetting layer is not scaled, the performance of the line Ru interconnect is comparable to that of Cu in the 5 nm node. Barrier/wetting layer scaling without losing EM and via chain yield is possible by employment of PVD/ALD-TaN and/or through-cobalt self-forming barrier (tCoSFB) [252,277,278]. Figure 41 depicts the scheme for tCoSFB [33,277]. When the thickness of TaN becomes thinner, this results in defects and TaN barrier performance becomes worse, which can be resolved by adding Mn to Cu. Mn will diffuse out of CuMn through the CVD-Co or Ru wetting layer to reach the ultra-thin TaN layer, forming Mn-O compounds to obtain good barrier properties. Compared to pure PVD-TaN layer, Mn-assisted TaN barrier has better EM performance [252,278]. For beyond 7 nm node BEOL, line R is assessed among four metallization schemes: Ru, Co, Cu with TaN/Ru barrier, and Cu with tCoSFB [278]. Line-R vs. linewidth of Cu fine wires with TaN/Ru barrier cross over with barrierless Ru and Co wires for beyond-7 nm node dimensions, whereas Cu with tCoSFB remains competitive, with the lowest line R for 7 nm and beyond. The tCoSFB scheme meets requirements in line R and EM reliability.

Sub-nm barrier is urgently demanded for ultra-scaled interconnects in the near future. To address this issue, 2D layered materials have been proposed and tested as diffusion barrier alternatives because of their atomically thin body thickness. Recently, graphene has been suggested as a new candidate for an ultrathin Cu diffusion barrier material due to its unique characteristics including excellent electrical and mechanical properties, thermal and chemical stability, and the ability to effectively block the diffusion of Cu atoms into ILD material [279,280]. For example, the graphene-capped Cu wires exhibit 2.4–3.5 × longer EM lifetime than thermally annealed Cu. The Cu resistivity is reduced by about 12–30% and the breakdown current density is increased by 5–7%. The graphene cap improves the Cu EM lifetime by mitigating Cu void formation at the interface [281]. Moreover, studies have shown that 2D layered single-layer molybdenum disulfide directly grown on SiO_2_ at 400 °C is achieved by metal-organic chemical vapor deposition (MOCVD). Results indicate that sub-nm MoS_2_ diffusion barrier can effectively stop Cu diffusion, significantly enhance dielectric lifetime, and is able to reduce Cu resistivity [282,283].

Carbon Nanotubes (CNTs) have been introduced to the design of transistors and have been experimentally shown to produce a higher scaling factor to the collector current when the scaling of the chip was larger than that of the current standard. Due to strong SP2 bonding between carbon atoms, CNTs are much less susceptible to EM problems than copper interconnects and can carry high current densities [284]. Comprehensive reviews about carbon nanotube interconnects are presented respectively by Uhlig [285] and Santos [286], and reviews include the following information: (1) Process and growth of carbon nanotube interconnects compatible with back-end-of-line integration, (2) modeling and simulation from atomistic to circuit-level benchmarking and performance prediction, (3) characterization and electrical measurements, and (4) previous research work on single-wall, multi-wall carbon nanotubes (MWCNT) and composite of copper and SWCNT (Cu-SWCNT). Carbon nanotubes, more specifically MWCNTs, have been identified as potential alternatives to diminish copper interconnect scaling issues [286]. However, using CNTs as interconnect material in semiconductor manufacturing is a huge paradigm shift, even higher than the change from aluminum (Al) to copper (Cu) in backend of line metallization [285]. Therefore, a lot of research efforts are still needed to realize the interconnection of carbon nanotubes.

## 13. Advanced Devices Reliablity

With CMOS technology development, the reliability issues of advanced CMOS technology are becoming more challenging and complicated due to the novel material, novel process and novel device structure, e.g., Ge/GeSi channels, atom layer etching (ALE) technology, nanowires and nanosheets, etc., specially for 5 nm node and beyond [287,288,289,290]. Next, the transistor reliability challenge of advanced CMOS will be discussed.

### 13.1. Process Dependent Reliability

Recently, novel devices such as FinFETs, nanowires, and nanosheets, as the best candidates for advanced CMOS technology, have been widely studied [287,288]. In order to meet the better performance of novel devices, advanced processes e.g., filling and etching in 3D device structure, are proposed [291,292]. Therefore, due to the diversity and complexity of advanced process, the reliability issues of advance CMOS show obvious process dependent characteristics.

**Figure 42:**
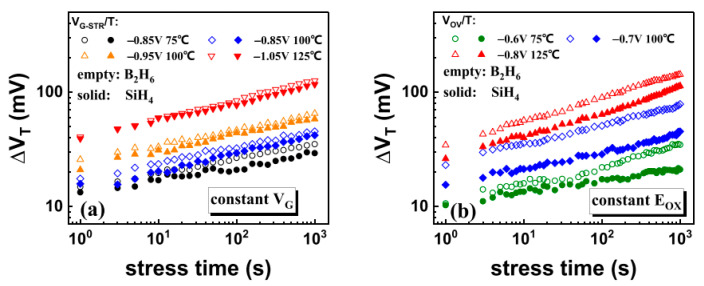
Threshold voltage shift (ΔV_T_) kinetics at different gate V_G-STR_ and T under constant V_G_ stress for ALD W using B_2_H_6_ and SiH_4_. (**a**) Under constant V_G_ stress; (**b**) under constant E_OX_ stress. The SiH_4_-based devices shows reduced negative bias temperature instability (NBTI) degradation than B_2_H_6_-based devices under both constant V_G_ and constant E_OX_ stress [295].

Take high-k/metal gate process as an example. As the filling metal, the atomic layer deposition Tungsten (ALD W) is widely applied due to better step coverage and conformity of deposited thin films. Usually, there are two kinds of ALD W process, one is using Diborane (B_2_H_6_) precursor, and the other is using silane (SiH_4_) [293,294]. The FinFET devices with different ALD W precursors show obvious sensitivity of electrical and reliability characteristics. In summary, the SiH_4_-based devices show better and faster negative bias temperature instability (NBTI) characteristics than B_2_H_6_-based devices, due to the fluorine incorporation and the compressive strain in the channel, as shown in Figure 42 [295].

Furthermore, the film thickness is another important elementary to reliability issues. The atomic layer deposition Titanium Nitride (ALD TiN) layer plays a very important role in CMOS integration [296,297,298,299]. The devices with different thickness of ALD TiN show the similar electrical parameters, while the reliability issues of devices are obviously different. Usually, the devices with thin ALD TiN layer show worse hot carrier injection (HCI) and better bias temperature instability (BTI) than that with thick ALD TiN due to the chlorine diffusion [10,296] and nitrogen diffusion [297,298], respectively. Therefore, it is key to restrain reliability degradation of advanced CMOS technology by controlling the process.

### 13.2. Small-Scale Related Reliability

In advanced CMOS technology, there are other key reliability issues concerned, such as self-heating (SH) and random telegraph noise (RTN), which are induced by small scale of 3D device [300,301,302,303,304,305,306,307,308].

**Figure 43:**
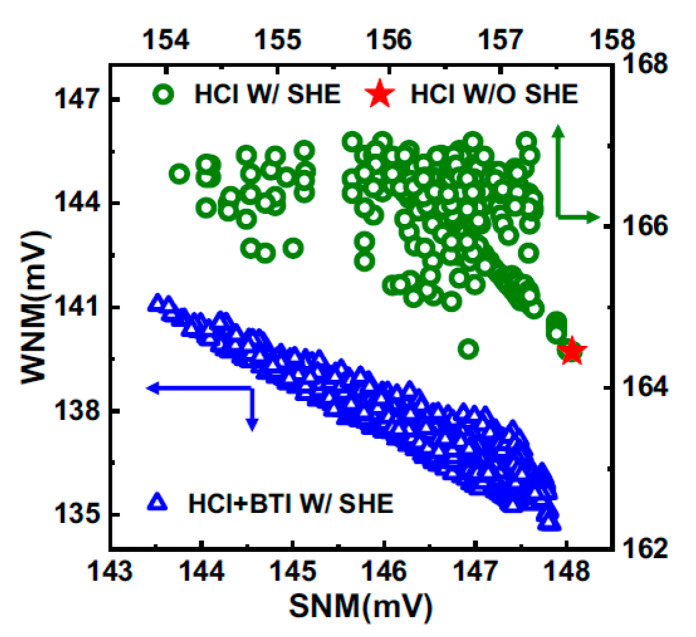
Simulated read static noise margin (SNM) vs. write noise margin (WNM) of the Static Random Acess Memory (SRAM) cell considering HCI degradation and HCI together with BTI degradation after 10^8^s operating time [304].

**Figure 44:**
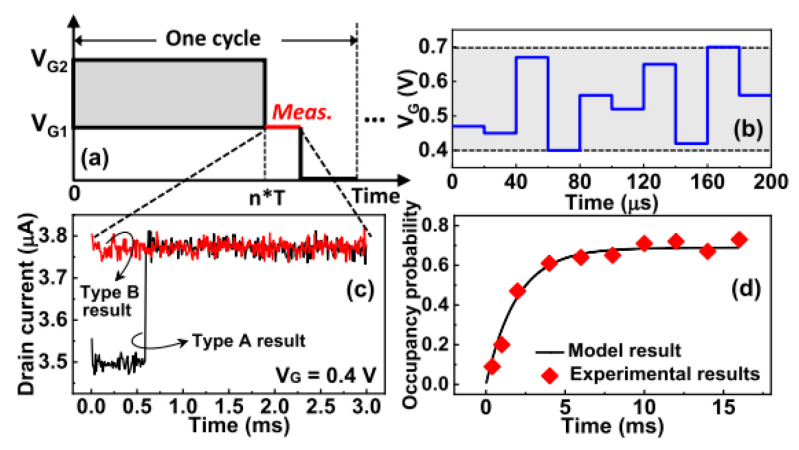
(**a**) Measurement schematic illustration for occupancy probability estimation, (**b**) randomly generated complex waveform for one period, (**c**) measured window to judge trap state after complex waveforms for each cycle, and (**d**) comparison between the extracted occupancy probability in experiments and model results [312].

Usually, self-heating (SH) is very sensitive to HCI and BTI [300,301,302], and self-heating of nanosheets could induce variability of devices, which makes the reliability of nanosheets more complicated, as shown in Figure 43 [303]. Furthermore, nanosheets show better resilience to a SH than FinFETs, and the SH of nanosheets is very sensitive to the structure of nanosheets, such as the width of nanosheets [304]. Actually, RTN is physically described using the “normal” two-state trap model, which is induced by single trap in a device [305,306,307,308,309,310,311]. It means that the two or more traps are independent and have a superposition effect. Recently, Wang et.al proposed a compact model for predicting the occupancy probability of switching traps for RTN, which is helpful for RTN reliability in variability-aware circuit design in advanced CMOS technology (see Figure 44) [312].

**Part Three: Materials in Beyond Moore Era**

## 14. III-V Materials

### 14.1. III-V Materials Growth

III-V compound semiconductors are regarded as superior candidates for Si-based NMOS channel materials due to their high electron mobility. For optoelectronic integrated circuits, III-V materials have a broad application in active and passive devices, such as lasers, detectors, and modulators. Therefore, III-V compound semiconductors have occupied an increasingly important role these years and a surge of interest among scholars has been aroused.

#### 14.1.1. Challenges in III-V Crystal Quality

Due to the different polarity of materials, which may lead to unexpected electrical impacts, the big difference in thermal coefficient, and the huge lattice mismatch between III-V materials and Si, for examples, about 4% for GaAs/Si interface, 8% for InP/Si, and 12% for InAs/Si, the heteroepitaxy of III-V materials based on Si have faced with the following challenges.

**Figure 45:**
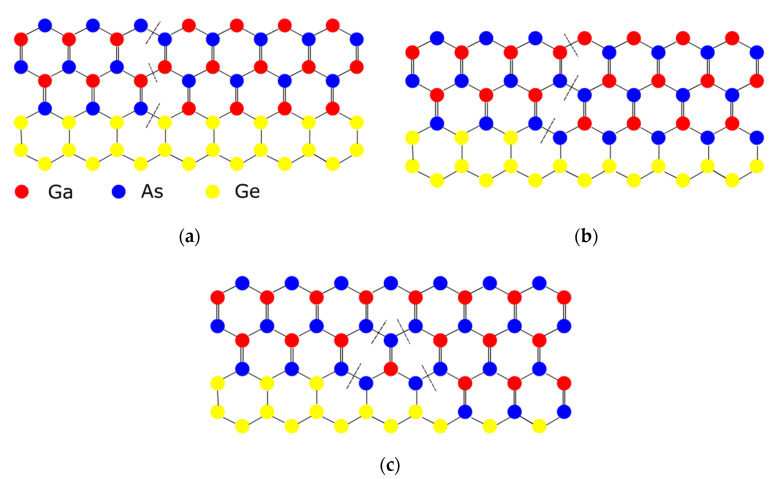
Antiphase boundaries (APDs) occurs when (**a**) GaAs is grown on an incomplete pre-layer coverage Ge substrate or with (**b**) odd atoms layer step, and (**c**) self-annihilating of APDs when GaAs is grown on cut-off substrate.

Compared with single element materials, the first challenge that III-V compound semiconductors need to overcome in heteroepitaxy is antiphase boundaries (APDs) [313,314]. Taking Figure 45 as an illustration, GaAs is grown on Ge with incomplete pre-layer coverage and several odd-atom steps are introduced in the surface during the process of material growth. Here, Ga atoms are illuminated as red dots while the yellow and blue dots stand for Ge and As atoms, respectively. Ga-Ga and As-As bonds, and sometimes a combination of these two situations, can be observed in GaAs epi-layer, as shown by the dashed line inserted in the picture. APDs will eventually result in unexpected nonradiative recombination surfaces and deterioration of the devices performance.

In state-of-the-art epitaxy, normal AsH_3_ or trimethyl Ga (TMGa) would first be utilized in the reactor for Si surface passivation and to prevent the incomplete pre-layer coverage. Unfortunately, however, odd atoms layer steps are still observed in (001) on-axis Si substrates. Therefore, cut-off substrates and other epitaxial technologies are employed, which will be discussed in next part.

In addition to APDs, some common defects such as threading dislocations (TDs) and stacking faults (SFs) also exist in epi-layers due to the lattice mismatch and the difference in thermal coefficient.

#### 14.1.2. Global Epitaxy

APDs, TDs, and SFs have a significant impact on the crystal quality of III-V materials grown on Si-based substrates. To suppress the dislocation density, some mature epitaxial technologies e.g., dislocation filter layers, buffer layer, low temperature/high temperature (LT/HT) technology, thermal cycling annealing, CMP after epitaxy, cut-off substrates, and necessary pre-treatment, are applied. It is worthy of note that the surface of cut-off substrate, of which oriented to (001) direction is 4°, 6°, etc., can effectively annihilate the APDs, as shown in Figure 45c. Another way to improve the epi-layer quality is to grow stacks of Ga-Ga or As-As bonds from two odd-atom steps converge and annihilate before it penetrates the whole GaAs epi-layer [315,316,317].

**Figure 46:**
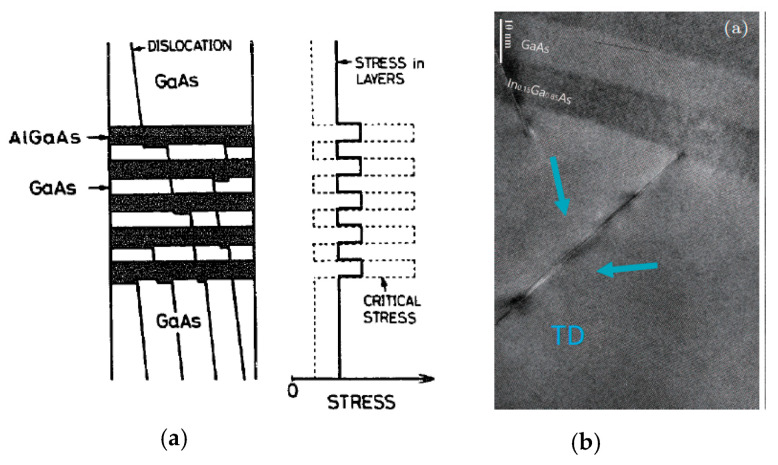
(**a**) Reduction of threading dislocation density (TDD) at layer interfaces in a superlattice (SL). Dislocations are bent at the interfaces in SL [321], and (**b**) a TEM image of a part of a SL structure with GaAs buffer layer grown by LT/HT multi-steps epitaxy [324]. The TEM image shows the TDs are restricted at InGaAs/GaAs interface and do not propagate further in the same direction.

Dislocation filter layers (DFLs) is an excellent method to reduce the threading dislocation density TDD) where superlattices (SLs) e.g., InGaAs/GaAs, AlGaAs/GaAs, and InAlAs/GaAs are grown [318,319,320]. Hayafuji et al. [321] reported that the mechanism behind SLs effect is the hardening of crystal and bending of dislocation along the superlattice interface. In addition, growth of buffer layer of Ge with high epitaxial quality, [322,323], LT/HT epitaxy (see Figure 46a), and CMP are popular methods to control the propagation of misfit dislocations, as shown in Figure 46b [324].

#### 14.1.3. Aspect Ratio Trapping Method

After Langdo et al. firstly reported the defects necking technology [325] and the aspect ratio trapping (ART) method, plenty of work was proposed to improve the quality of epi-layers. Based on ART and CMP, a high-quality Ge crystal can be obtained on Si substrate. Thanks to the similar lattice coefficients (αGe = 5.658Å, αGaAs = 5.653Å), a low dislocation density GaAs layer can then be prepared on the Ge/Si substrate [326].

**Figure 47:**
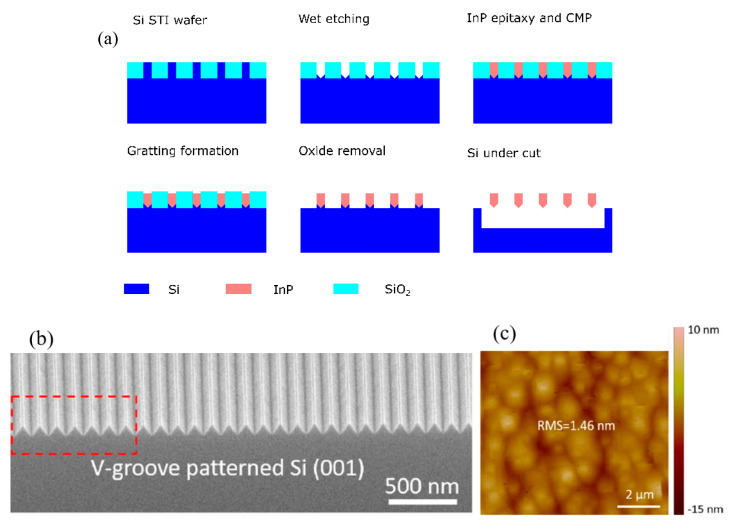
(**a**) V-groove aspect ratio trapping (ART) process flow, (**b**) the SEM diagram of the tilted V-groove patterned Si and, (**c**) the atomic force microscope (AFM) of GaAs on patterned Si, respectively [333].

In recent years, V-grooved trench Si substrates has been discussed by Wang, et al. [327] as an alternative to ART method. Figure 47a shows the process flow and the outcome from V-groove trench method. Compared with conventional InP epitaxy, V-grooved trenches technology adopts potassium hydroxide (KOH) solution to form (111) orientation as initial growth surface to suppress the APDs. The idea behind this is to capture TDs in the V-groove and, thus, a dislocation-free epi-layer can be later obtained with a thin buffer layer.

Integrated with other epitaxial technologies, GaAs, InP, and even quantum wells on Si, Ge, SOI, GOI substrates are demonstrated in [328,329,330,331,332]. As an example, Shi [333] made use of InGaAs/GaAs SLs and thermal cycling annealing to achieve high-quality InAs/InGaAs/GaAs quantum dots (QDs) structure on V-grooved patterned Si in Figure 47b,c. The surface roughness reported in root mean square was 1.46 nm while threading dislocation density reached 9.1 × 10^6^ cm^−2^.

### 14.2. III-V High Mobility Transistors

**Figure 48:**
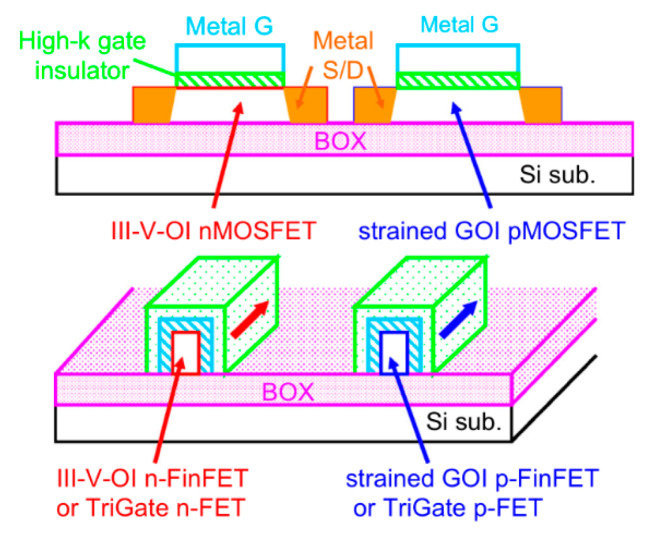
Structures of complementary metal oxide semiconductor (CMOS), which are composed of III-V nMOSFET and Ge p type Metal-Oxide-Semiconductor Field-Effect Transistor (pMOSFET) [342].

As the development of silicon-based electronics has approached the limit of scaling for increasing the performance and chip density, interest has been greatly increased in introducing non-traditional materials as high mobility channels for devices beyond the 14 nm technology node. III-V compound semiconductors have started to attract significant attention owing to their higher intrinsic mobility than Si, which can potentially be used to replace Si as the material of transistor channel [334]. III-V transisitors, such as heterojunction bipolar transistors (HBT), metal-semiconductor field effect transistors (MESFETs), and heterojunction field-effect transistors (HFETs), which includes high electron mobility transistors (HEMTs), has extended the advantages of silicon counterparts to significantly higher frequencies and become natural choices of device for wireless communication [335]. Recent innovations on III-V transistors include high-performance InGaAs buried channel [336,337], surface channel MOSFETs [338], sub−80 nm E–mode InGaAs/InAs HEMTs [339,340], and InSb p-channel HEMTs [341] with outstanding logic performance at short channel lengths and low supply voltages. In order to optimize the performance of CMOS, it has also been put forward that we can combine Ge with III-V materials. Typical cases are shown in Figure 48, which depicts the III-V nMOSFETs and Ge pMOSFETs [342].

**Figure 49:**
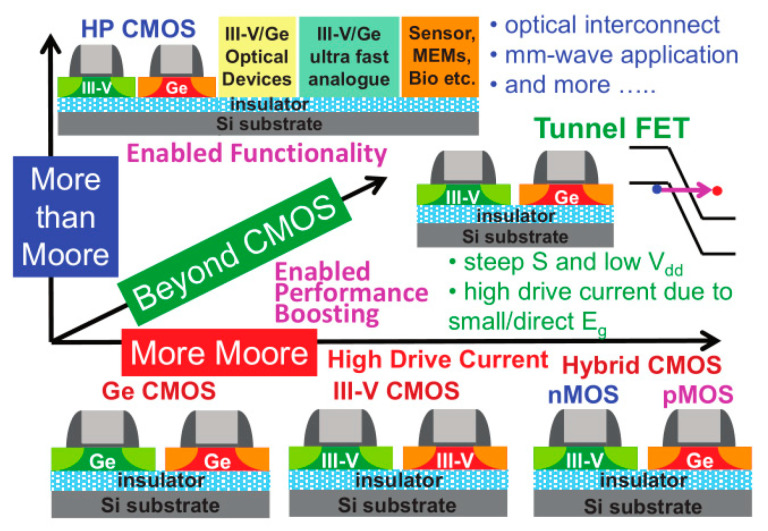
Possible evolution scenario for III–V/Ge devices on Si platform through heterogeneous integration [343].

The integration of Ge/III-V with the Si CMOS platform is a promising project for realizing ultra-low-power integrated systems in the 10 nm technology node and beyond. Figure 49 shows a variety of possible applications of III–V/Ge materials on the Si CMOS platform [343].

**Figure 50:**
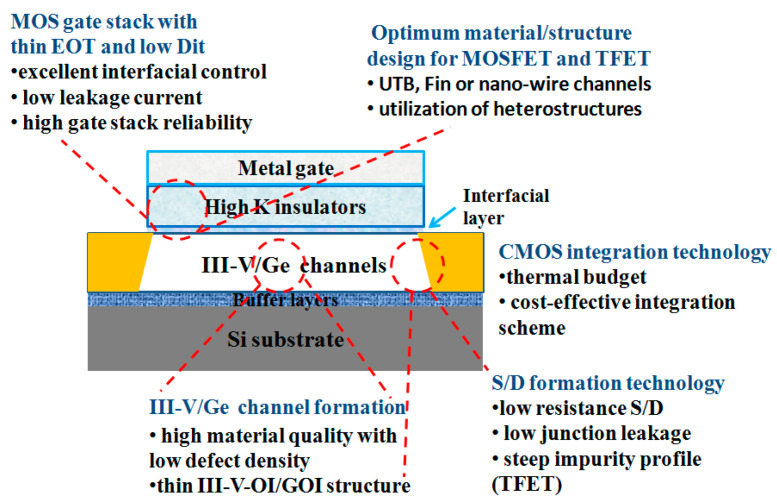
Critical issues of Ge/III-V integration with Si CMOS platform [344].

The integration of a variety of functional devices including IV/III-V-based optical interconnect components with advanced CMOS is also regarded as the possible path for realizing a system with low power consumption under the given system performance. However, there are many critical issues for preparing IV/III–V-based CMOS and TFETs on the Si platform [344], as shown in Figure 50. These challenges can be concluded as: (1) Obtaining high-crystal-quality IV /III-V layers on Si substrates, (2) exploring process on gate stack to realize superior MOS interface quality, (3) MOSFETs and TFETs with low resistivity, low leakage current, and steep impurity profiles for S/D, (4) integration of process to realize ultra-short channel devices, and (5) total CMOS integration including Si CMOS.

**Figure 51:**
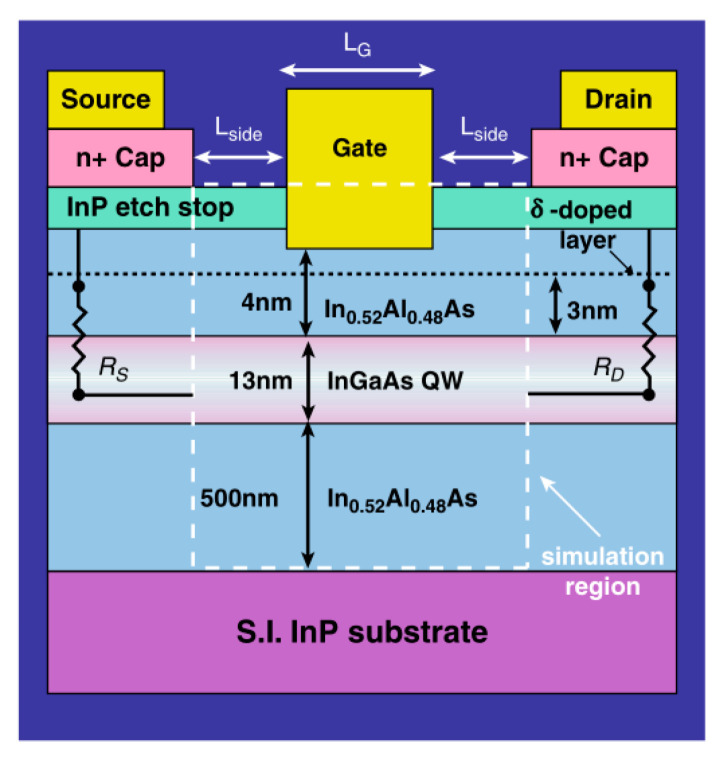
The structure of InGaAs high electron mobility transistors (HEMTs) designed for logic applications [345].

Kim et al. [345] investigated the impact of lateral and vertical scaling of In_0.7_Ga_0.3_As HEMTs on their logic performance, and the HEMT structure for logic applications, as shown in Figure 51. The outcome reveals that reducing the In_0.52_Al_0.48_As insulator thickness results in a better electrostatic integrity and the short-channel behavior is improved down to a gate length of around 60 nm. Characteristics of this enhancement-mode of 60-nm HEMTs are: V_T_ = −0.02 V, DIBL = 93 mV/V, S = 88 mV/V, and I_ON_ /I_OFF_ = 1.6 × 10^4^, at V_DD_ = 0.5 V.

For devices such as the field effect transistor (FET), the current conduction is dominated by the drift of the majority carrier, and thus the majority carrier becomes the critical parameter that limits the transconductance: g_m_ = dI_DS(sat)_/dV_Gs_∞μ/L; where L refers to the channel length of the FET structure. This measurement is a characterization of variation in the channel current per gate bias change. With less charge transport to the gate electrode to achieve a fixed change in the channel current, high transconductance allows fast switching and large signal to noise ratio, making it an important figure of merit in high frequency FET applications. Electron mobility of 1500 cm^2^/Vs is a typical value at low doping level in Si, but III-V compounds (GaAs in particular) are used for their higher intrinsic mobility [346]. Hur et al. [347]. presented a theoretical model that describes the degradation of carrier mobility by charged dislocations in quantum-confined III-V semiconductor metal oxide field effect transistors (MOSFETs). They chose In_0.53_Ga_0.47_As as a channel material because In_0.53_Ga_0.47_As is the most popular III-V channel material used in field effect transistors owing to its extremly high electron mobility and relatively small lattice mismatch with silicon substrate.

**Figure 52:**
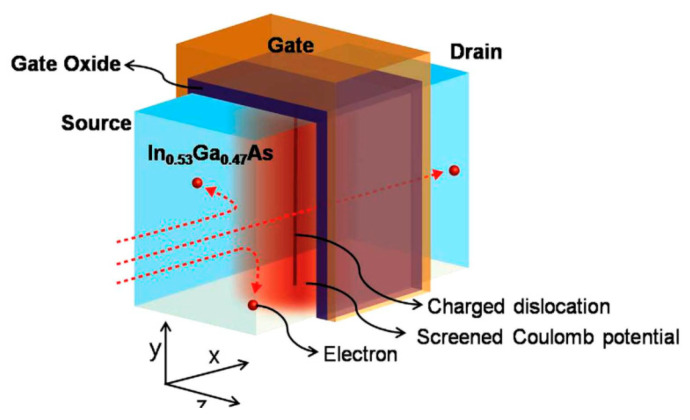
Schematic illustration of the III-V semiconductor channel fin-shaped field effect transistor with a charged dislocation located at center of channel and vertical to the top gate surface [347].

Figure 52 illustrates the carrier scattering events in In_0.53_Ga_0.47_As FinFET with a charged dislocation located near the center of the channel, which is fully charged, screened by the environmental free carriers, and vertical to the top gate. From the calculated dislocation mobility, they concluded that a nano-scale III-V compound semiconductor device loses its merits over silicon if it bears inevitable dislocations (when fabricated on silicon wafer). Scattering due to dislocation could be the dominant mechanism in quantum-confined, short-channel MOSFETs. This kind of severe degradation of the effective channel mobility would lead to malfunctioning of the transistor cell, eventually resulting in the failure of the entire logic chip.

By virtue of their high electron and hole mobilities, InGaAs and Ge have emerged as the most promising candidates for n-and p-MOS, respectively. Gang Wang et al. used the ART technique to fabricate InGaAs-based devices on 200 mm Si wafers and to create virtual III–V/Ge substrates.

**Figure 53:**
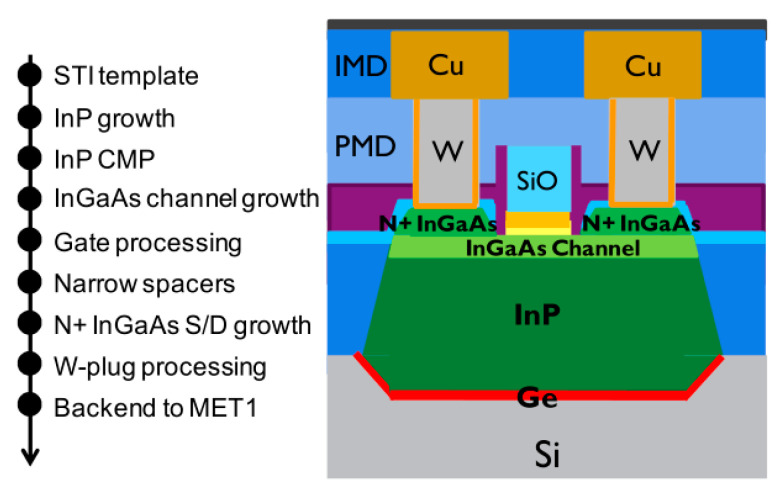
Process flow of InGaAs devices fabricated on 200 mm Si wafers and schematic of transistor structure [348].

Figure 53 depicts the InGaAs devices process flow and the schematic of transistor structure [348]. Electrical characterization of the devices shows that there is a clear gate modulation effect on top of the high level of source to drain leakage. This leakage was determined to be the result of the unintentional n-type doping of the InP buffer layer.

### 14.3. III-V as Optoelectronic Materials

For decades, integrated circuits based on Si and Ge have triggered a modern industrial revolution, which has profoundly contributed to the change of our social life. As the first generation of semiconductor materials, Si-based devices can be well integrated with digital or analog circuits, which is convenient for the design and implementation of system-on-chip (SoC). Due to the characteristics of material and bottleneck of process, however, the application of Si devices in high-frequency scenes is limited, and they are mainly used in S-band and below. In contrast, III-V compound semiconductor devices with excellent electron mobility, high saturated velocity, and luminous efficiency are considered to be the mainstream solutions in the areas of microwave and millimeter wave communication, ultra-high speed digital circuits, optoelectronics, etc.

#### 14.3.1. Characteristics and Applications of InP

Devices suitable for civilian and military applications, such as solid-state luminescence, microwave and optical fiber communication, and navigation can be fabricated by relying on III-V compound materials. Taking the commonly used InP and GaAs as examples, efficient electro-optical conversion benefits from the direct bandgap structure, which causes them to become appropriate choices for devices like light emitting diodes (LEDs) and laser diodes. Low-power high-frequency microwave integrated circuits are promoted because of the characteristic of high electron mobility. Owing to the outstanding thermal stability and strong radiation tolerance, they are also adopted in the fields of aerospace and solar cell as well [349]. Comparatively speaking, GaAs has attracted people’s attention earlier and the cost is cheaper while InP has a later start but rapid development due to the advantages that other materials do not. In the range of 40~50 GHz, GaAs-based devices occupy the dominate role because of the lower cost and mature technology, while InP-based devices are preferred for frequency over 75 GHz. InP is widely used as substrates for electronic and optical devices such as HEMTs, HBTs, LEDs, etc. Some featured applications involving InP HEMTs are summarized in Table 2.

In addition, thermal conductivity of InP is higher than that of GaAs, which means that the temperature of InP-based device is lower, and the output power is higher when the device operates under the same power. When it comes to the optical communication system, the operating window of quartz fiber is 1.3~1.55 μm which is because the dispersion of optical fiber is close to zero near 1.3 μm and the transmission loss is exceedingly low near 1.55 μm. Thanks to the matching of lattice constant, InP is often used to prepare the necessary photoelectric devices in these systems such as InGaAs detectors, InGaAsP lasers, AlGaInAs modulators, etc. At present, lasers, modulators, detectors, and integrated modules with fabulous monochromaticity based on InP are used widespreadly in optical communication network [350].

#### 14.3.2. Structures and Principles of InP HEMTs

**Figure 54:**
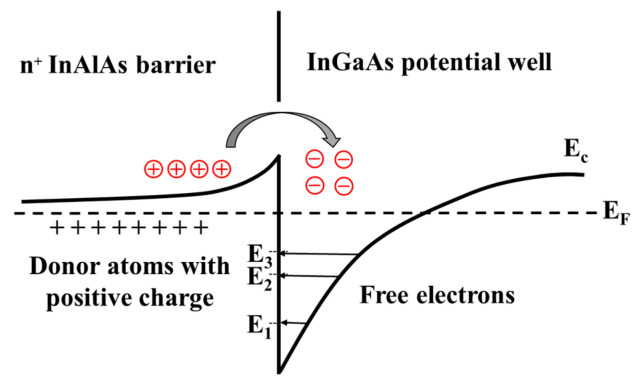
Band structure of n^+^ InAlAs/InGaAs heterojunction.

The particular thing about HEMT devices lies in the introduction of heterojunction to form a two-dimensional electron gas (2DEG) channel layer apart from the metal-semiconductor contact, which forms the Schottky contact and source-drain ohmic contact [358]. When a heterojunction is formed by two kinds of materials with different bandgap, electrons will be transferred between two layers as a result of the difference of Fermi levels, which lead to the energy band bending on the interface. As illustrated in Figure 54, the electrons in InAlAs will be transferred to the undoped InGaAs with lower energy and accumulated at the interface. Ionized donor atoms with positive charge thereby are left in InAlAs. An extremely thin potential well is generated, consequently, and the energy band bends at the same time. The thickness of this thin potential well is usually a few nanometers, which is almost the same as the wavelength of the electron, and thus the electron energy in the potential well is quantized in the direction perpendicular to the interface. The electrons can move freely in the two-dimensional direction parallel to the interface but the movement in the direction perpendicular to the interface is restricted, which gives rise to the 2DEG. In fact, the electrons in InGaAs well are provided by the doped atoms in InAlAs, which realized the separation of electrons and donor atoms in space, resulting in the reduction in the scattering of ionized atoms and improvement of the electron mobility. Therefore, such a structure is also known as a modulation doped structure. The electron mobility can be further enhanced if an undoped spacer layer is deposited between the barrier layer and the 2DEG layer. It will decrease the Coulomb scattering caused by the interaction of doped atoms with channel electrons.

**Figure 55:**
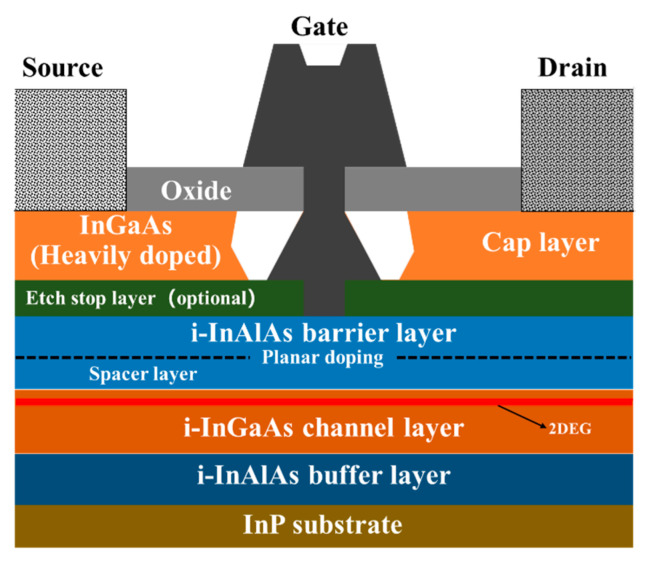
A typical structure of InP HEMT [358].

Figure 55 is a schematic of a typical InP-based HEMT [358]. The device structure consists of, from bottom to top, an InAlAs buffer layer, an InGaAs channel layer, an InAlAs spacer layer, a carrier supply layer or δ-doping plane, and an InAlAs barrier layer. These layers are grown on InP substrate. Three electrodes are generally included in InP HEMT, which are source, drain, and gate. The spacer layer, heavily doped layer, and barrier layer are grown with the same materials, which has a wide bandgap, and the channel layer is made of narrow bandgap material. Also, upon the barrier layer, InGaAs cap layer with heavy doping is often used to ensure low contact resistance for the S/D ohmic electrodes. Schottky contact is made by the etching of cap layer at the portion of the gate electrode. The height of barrier has a great influence on the leakage current of the gate, and it determines the breakdown characteristics of this device. The shorter the gate, the faster electrons are transported through the channel, thus the operating frequency of devices is increased. However, it may result in a larger resistance of gate electrode. Under this circumstance, the conventional solution is to design the gate as T-shape in order to ensure the decreased transmission time and small gate resistance. Transistors that are able to present current gain cut off frequency ($f_{T}$) and maximum oscillation frequency ($f_{\max}$) beyond 640 GHz simultaneously are pseudomorphic InAlAs/InGaAs HEMTs grown on InP substrates [359].

In addition to the lattice matched InAlAs/InGaAs heterojunction, sometimes the other structure design is applied in monolithic microwave integrated circuit (MMIC), which takes InP as the substitution material for half of the InGaAs channel. This is due to the strong collisional ionization in the InGaAs channel. The generation of holes causes a decrease in breakdown voltage, an increase in output conductance, and gate current, which has an intense impact on the noise coefficient of the low-noise amplifier (LNA). InP has a high ionization threshold energy (~1.69 eV) than that of InGaAs (~0.92 eV) and the characteristics of the electron transport in InP are different from those in InGaAs when high- field is operated. Chevalier et al. observed that the composite channel improved significantly in conductance, transcondutance, and cut-off frequency [360]. The importance on RF performance of electron confinement, impact ionization, and parasitic capacitance influenced by layer sequence and process are also investigated. Furthermore, Chin et al. adopted the InAs/InAlAs superlattice to overcome the lattice mismatch and obtained the InAs channel on the InP substrate successfully. Profiting from the increased In composition in the In_0.53+x_Ga_0.47−x_As channel, the lower electron effective mass, higher electron mobility, peak velocity, and better carrier confinement are achieved in the quantum well [361].

#### 14.3.3. Developments of InP HEMTs

**Figure 56:**
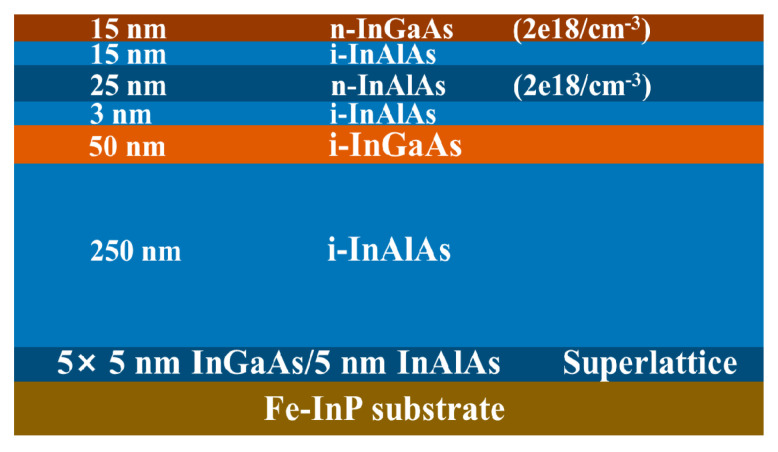
Layer structure of the first HEMT device based on InP.

The types for heterojunction channels in HEMT are mainly available in three: Flavors-pseudomorphic HEMT (pHEMT), metamorphic HEMT (mHEMT), and induced HEMT (iHEMT) [362]. The representative material systems have been discussed in the past few decades such as AlGaAs/GaAs [363], AlGaN/GaN [364], and InAlAs/InGaAs [365]. In 1980, the first HEMT transistor was demonstrated in Fujitsu Labs with selectively doped GaAs/Al_x_Ga_1−x_As heterojunctions [366] based on the concept proposed by Ray Dingle and collaborators in 1978 [363]. Results described a prominent improvement in HEMT mobility than that of the GaAs MESFET in 77K and 300K and they had a rough estimation that the high-speed performance of HEMT is capable of being 3-times superior to the MESFET. The first InP-based HEMT device was prepared in 1986, as shown in Figure 56, which has a tremendous superiority in power gain and current gain ($f_{T}$ = 30 GHz, $f_{\max}$ = 62 GHz) than that of the GaAs pHEMTs ($f_{T}$ = 23 GHz, $f_{\max}$ = 40 GHz) with comparable geometry [367].

Compared with the AlGaAs/InGaAs interface of the GaAs-based HEMT, the InAlAs/InGaAs interface material of the InP-based device has higher channel electron mobility, superior gain performance, and low noise figure. On the other hand, the discontinuity of conduction band of the InAlAs/InGaAs interface is more obvious than the AlGaAs/InGaAs interface and the concentration of electron gas in the channel is higher, which leads to the enhancement of gate control effect as well. Meanwhile, it is argued that $f_{\max}$ in excess of 1 THz [368] and a noise figure as low as 0.71 dB at 95 GHz [369] can be achieved in InAlAs/InGaAs pHEMTs based on InP. In 1992, InP-based HEMT device with low-conductance drain (LCD) design for lattice-matched InAlAs/InGaAs/InP structure was successfully developed by increasing the effective gate length of the HEMT in exchange for reduced peak channel electric field [370]. Both InGaAs with high electron mobility and InP with the high drift velocity are exploited at low electric field and high electric field respectively in a distinctive HEMT channel structure proposed in the same year [371]. It is also worthy of note that there is no kink in the I–V characteristics of double channel devices and this phenomenon indicates the InGaAs/InP double channel HEMTs can be well fitted in high-speed and high-power applications. The best output power of 130 mW measured from a single fixtured InP-based MMIC at 94 GHz in 1997 is a W-band MMIC amplifier with passivated 0.15 um gate length, which takes advantage of the InGaAs/InAlAs/InP HEMTs [372]. The first amplifier whereby a striking amount of gain was observed at sub-millimeter wave frequencies is a three-stage LNA, which makes use of the advanced InP HEMTs, and 16 dB gain at 340 GHz and >20 dB gain at 280 GHz were realized [373]. An essential attempt for 850 GHz amplifier was reported by the utilization of InP HEMTs, and this nine-stage amplifier reaches a peak gain of 6 dB at 850 GHz, which becomes promising for THz imaging and communications [374]. Lai et al. reported the $f_{\max}$ of over 1 THz for the first time using 50 nm InP HEMTs technology [368] and then Mei et al. improved $f_{\max}$ to 1.5 THz by employing 25 nm InP HEMTs process [375]. The physical properties of several representative InP HEMTs are summed up in Table 3.

## 15. Two-Dimensional Materials

**Figure 57:**
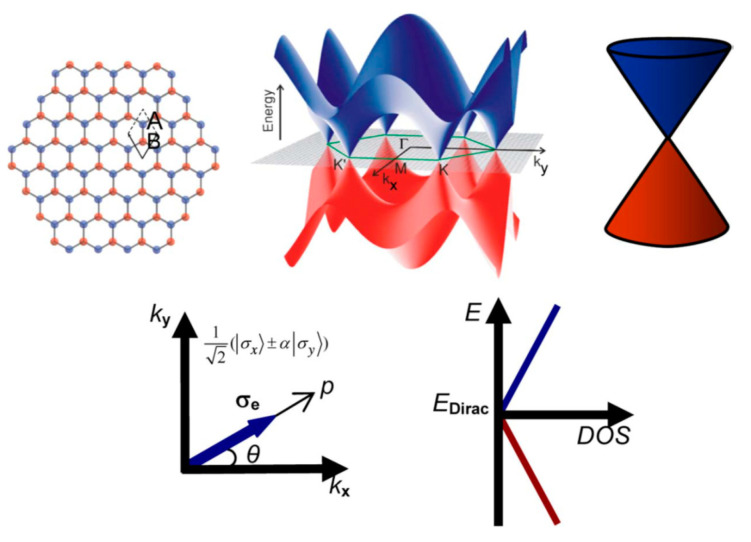
Left to right: Graphene lattice, electronic bandstructure, linear dispersion at low energies, pseudospin components, and density of states (DOS) dependence on energy [384].

Two-dimensional (2D) materials refer to materials that reach the nanoscale in a certain direction and exceed the nanoscale in the other two dimensions. Due to its special morphology and the high anisotropy of crystal growth, electrons can generally move freely in two dimensions, thus exhibiting special electrical properties. Graphene is a 2D carbon nanomaterial with hexagonal honeycomb lattice structure composed of carbon atoms with sp^2^ hybrid orbitals. In 2004, Novoselov et al. successfully prepared graphene with a single carbon atom layer from graphite by mechanical exfoliation, proving that graphene can exist alone [383]. Because of the lack of a bandgap, graphene is usually described as a zero-gap semiconductor, or better yet, as a semimetal (Figure 57) [384]. This semi-metallic graphene has ultra-high carrier mobility and exhibits excellent mechanical, electronic, and electromagnetic properties. After the successful separation of graphene, the concept of 2D materials was formally proposed and found to have promising applications in many devices.

In addition to graphene, other 2D materials can be included e.g., single-elemental silicene, germanene, stanene, black phosphorus, etc.; transition metal dichalcogenides (TMDs) such as MoS_2_, WSe_2_, ReS_2_, PtSe_2_, etc.; semimetal chalcogenide (SMCs) such as GaS, InSe, SnS, SnS_2_, etc.; and other 2D materials such as h-BN, CrI_3_, NiPS_3_, Bi_2_O_2_Se, etc. These 2D materials have completely different energy band structures and electrical properties, covering material types from metals, semi-metals, and semiconductors to insulators. By stacking 2D materials of different types, a more functional material system can be constructed. With its advantages of high specific surface area, strong electrical and thermal conductivity, high light transmittance, and high carrier mobility, 2D materials have broad application prospects in the fields of high-performance electronic devices, optoelectronic devices, energy storage, and sensors.

**Figure 58:**
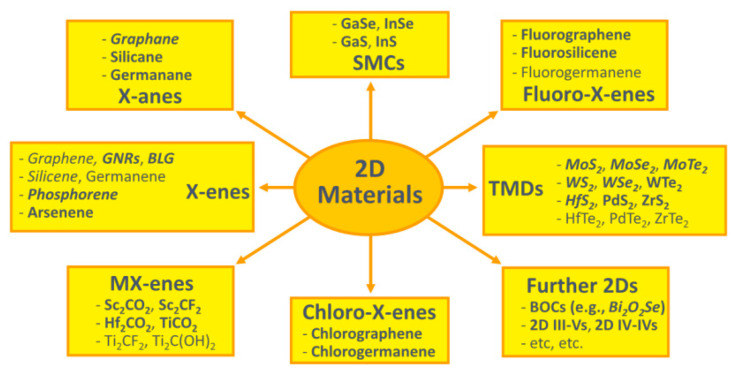
Drawing of different 2D materials [385].

Classes and representative examples of 2D materials together with representative materials for each group are shown in Figure 58 [385].

### 15.1. Synthesis Methods for 2D Materials

In recent years, people have developed a series of methods to prepare high-quality thin layers or single-layer structures of 2D materials. The preparation methods are mainly divided into two categories: “top-down” exfoliation methods and “bottom-up” synthesis methods [386]. Due to the particularity of its structural dimensions, 2D materials have many excellent properties, which provides more possibilities for the design and application of new functional materials [387]. A series of 2D materials have been successfully prepared by micromechanical exfoliation, liquid exfoliation, chemical vapor deposition, and hydrothermal methods.

#### 15.1.1. Micromechanical Exfoliation

Graphite, molybdenum disulfide (MoS_2_), hexagonal boron nitride (hBN), and other materials exist in the form of stacked 3D layers. These materials are combined by the weak van der Waals force, and 2D layered materials can be obtained through micromechanical exfoliation. To understand the basic properties of the materials, the single layer should be separated from the bulk materials. However, it was believed that the structure of the 2D materials would undergo structural deformation without the support of the substrate due to thermal fluctuations, and even lead to structural damage [388], which did not attract enough attention. Micromechanical exfoliation is a traditional and mature method to prepare 2D material. It could break the weak van der Waals force between the bulk crystal sheets without breaking the covalent bonds in the plane of each layer to prepare 2D materials. At present, this method has been used to obtain single-layer 2D materials of WS_2_, h-BH, Bi_2_Sr_2_CaCu_2_O_x_ [389]. However, on the other hand, it results in low productivity, and is not suitable for large-scale production.

#### 15.1.2. Liquid Exfoliation

The basic principle of the liquid exfoliation method is to dissolve the layered material in a solution with its matching surface energy and exfoliate 2D materials by means of ultrasonic cleavage and shear force. Compared with traditional mechanical exfoliation, liquid exfoliation is a reliable method for mass production of single- or few-layer 2D materials. Liquid exfoliation can be divided into three main different categories: (i) Mechanical forces, (ii) ion intercalation or ion exchange (iii) oxidation.

Mechanical forces refer to the methods of applying mechanical forces such as ultrasonic waves and shear forces to the bulk materials in the liquid to destroy the van der Waals force between the layers to obtain single-layer 2D nanosheets. Generally, the specific solvent such as NMP, DMF, DMSO, IPA, etc., are all commonly used solvents for ultrasonic exfoliation. The use of these solvents with good tension matching to the surface of the crystal layer can minimize the energy consumption of exfoliation and prevent the re-stacking and reunion of nanosheets [390]. In addition to graphene, various materials such as TMDs, h-BN, and TMOs can also be prepared by this method [391]. This method is simple but the size of the obtained nanosheet is small, and it is easy to introduce defects and impurities. The shear method utilizes the shearing force generated by the rotor-stator agitator to exfoliate. Coleman et al. demonstrated the use of a rotor-stator mixer to exfoliate graphene from graphite [392], and then used a mixer to exfoliate h-BN, MoS_2_, and WS_2_ [393]. The shear force method has high exfoliating efficiency, which has been applied to the commercial production of graphene.

**Figure 59:**
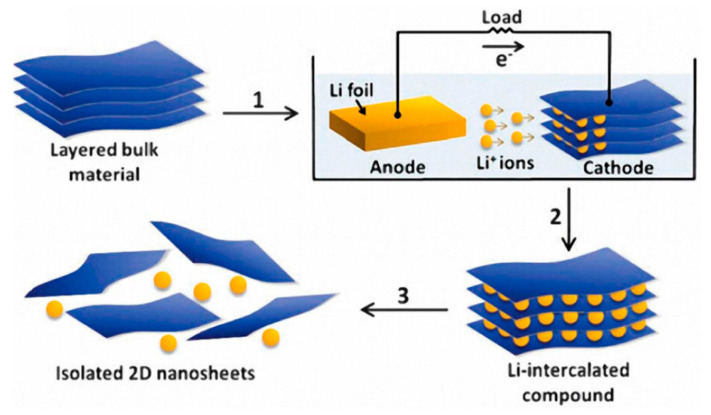
Schematic illustration of the electrochemical Li intercalation-assisted liquid exfoliation method for preparation of single- or few-layer transition metal dichalcogenide (TMD) nanosheets [394].

Ion intercalation or exchange refers to the insertion or replacement of ions existing on the base surface between the layers of bulk materials, which can weaken the interlayer adhesion and increase the interlayer spacing to facilitate subsequent mechanical force exfoliation. The methods of ion intercalation can be divided into chemical intercalation and electrochemical intercalation. It has high yield, but the disadvantage is that the process is complicated, and impurities are easily introduced. Graphene, h-BN, and many TMDs can be prepared using this method [394] (see Figure 59). The ion exchange method can be divided into cation- and anion-assisted exchange. This method can efficiently produce specific 2D materials in large quantities. Due to the presence of chemical reactions, the preparation process may change the electrical properties of the material and limit its application in electronic devices. 2D materials such as metal oxides, metal phosphorus tri-chalcogenides, and LDHs nanosheets can be prepared using ion exchange method.

Oxidative liquid exfoliation refers to a method used to synthesize graphene oxide. Graphite flakes are treated with strong oxidants such as sulfuric acid or nitric acid to introduce OH or epoxide functional groups on the surface, which increase the interlayer distance and facilitate subsequent exfoliation [395]. This method can effectively produce graphene oxide, but it is difficult to apply it to the preparation of other 2D materials since the introduction of strong oxidants increases the test risk.

#### 15.1.3. Chemical Vapor Deposition (CVD)

**Figure 60:**
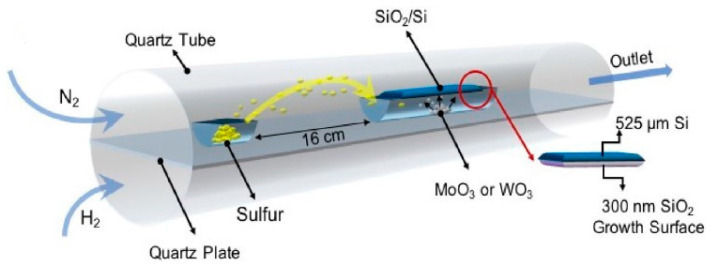
Schematic of the growth of chemical vapor deposition (CVD) system for MoS_2_ and WS_2_ [396].

The micromechanical exfoliation and liquid exfoliation mentioned above are both “top-down” methods. CVD, a “bottom-up” technique, can introduce one or several gas-phase precursors into a high-temperature reaction furnace, thus it can grow a 2D material with a large area and a uniform thickness on the surface of a metal or insulating substrate. This method can be used for large-scale device manufacturing of electronic and optoelectronic devices. The structure of the metal substrate is simple, which is conducive to the study of the growth mechanism. However, due to contact interference between metal and devices, the 2D materials on the metal substrates cannot be directly used for property measurement and device design. It needs to be transferred to an insulating substrate for further applications. The 2D materials grown on semiconductors or insulating substrates can be directly used in subsequent applications. Figure 60 is a schematic diagram of a CVD method for growing MoS_2_ and WS_2_ on the surface of SiO_2_/Si substrates [396]. Lee et al. synthesized a 0.72 nm thick single layer of MoS_2_ using MoO_3_ and element S at 650 °C in a one-step bottom-up method [397]. The method can achieve high crystallinity, high purity, and high yield preparation of various 2D materials including BN and transition metal dichalcogenides (TMDs) with controllable material size, thickness, and composition, etc. It is expected to be applied in the industrial production of ultra-thin materials, but it also has higher production costs.

#### 15.1.4. Hydrothermal Synthesis

**Figure 61:**
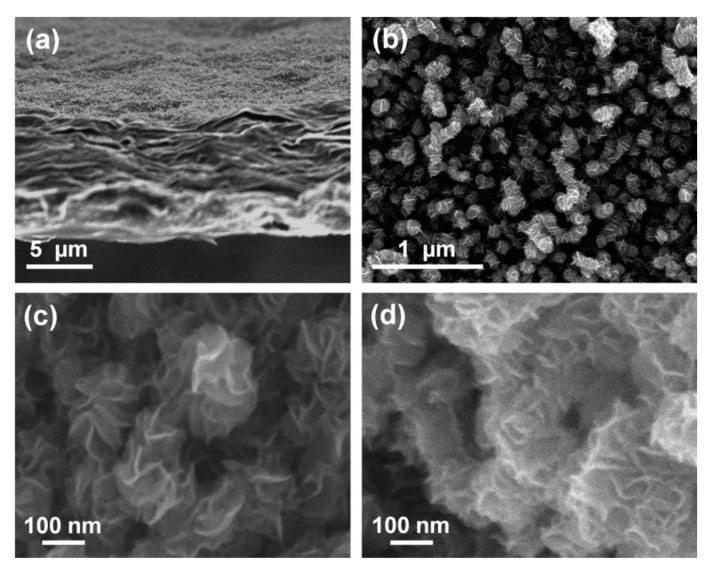
SEM images of (**a**) the cross-section and (**b**) the top view of MoS_2_NF/rGO. Highly magnified SEM images of the top view of (**c**) MoS_2_NF/rGO and (**d**) MoS_2_AG/rGO [399].

Hydrothermal synthesis refers to a “bottom-up” method for growing nanomaterials in aqueous or organic solution at high temperature and high vapor pressure in a closed reaction vessel. This method is particularly suitable for precursors with best solubility and growth phase stability under high temperature and pressure. Wang et al. prepared h-BN of ~4 nm thickness on the gram scale from boric anhydride, Zn powder, and N_2_H_4_*2HCl at 500 °C [398]. Ma et al. developed a facile method to synthesize MoS_2_ nanoflowers (MoS_2_NFs, as shown in Figure 61 coated on reduced graphene oxide (rGO) paper. The MoS_2_NF/rGO electrode exhibits good stability with negligible current loss, even after 300 cycles in the durability test [399]. The application of hydrothermal methods has been extended to obtain other 2D layered materials. These synthetic 2D materials can be deposited to prepare nanocomposites, nanodevices, and future nanotechnology applications. The hydrothermal method can prepare 2D materials with high crystallinity and low cost but have the disadvantages of invisibility of the reaction process, uncontrollable film thickness, and longer reaction time.

### 15.2. Nano-Scale Device Applications of 2D Materials

#### 15.2.1. Electronic Devices

**Figure 62:**
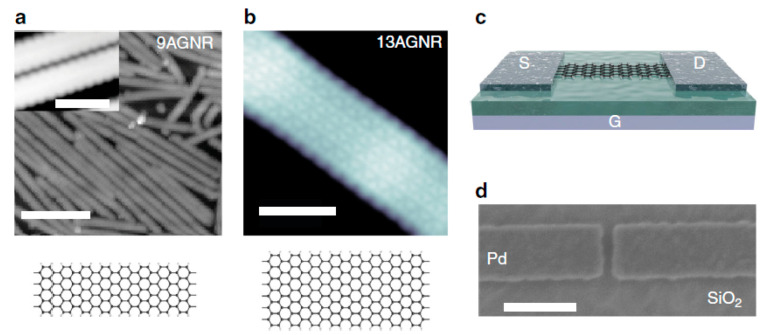
High-resolution scanning tunneling microscope (STM) graphene nanoribbons (GNR) characterization and FET structure: (**a**) STM image of synthesized 9AGNR on Au with a scale bar of 10 nm (V_s_ = 1 V, I_t_ = 0.3 nA). Inset: High-resolution STM image of 9AGNR on Au (V_s_ = 1 V, I_t_ = 0.5 nA) with a scale bar of 1 nm, (**b**) high-resolution STM image of 13AGNR on Au with a scale bar of 2 nm (V_s_ = −0.7 V, I_t_ = 7 nA), (**c**) schematic of the short channel GNRFET with a 9AGNR channel and Pd source-drain electrodes, and (**d**) scanning electron micrograph of the fabricated Pd source-drain electrodes with a scale bar of 100 nm [400].

Some 2D materials have outstanding electrical and mechanical properties, which are suitable for electronic devices. Novoselov et al. [383] reported the first graphene-based field effect transistor (FET), exhibiting outstanding bipolar electric field effects. Shortly afterwards, Llinas et al. [400] demonstrated FETs made of graphene nanoribbons (GNR), which is only 9 atoms wide as shown in Figure 62. The devices with a thin HfO_2_ gate dielectric (effective oxide thickness of around 1.5 nm) exhibited high on-current (I_on_ > 1μA at V_d_ = −1 V) and high I_on_/I_off_ ~ 10^5^ at room temperature. However, the inherent zero band gap effect of graphene severely limits the efficiency of FETs and their applications in logic circuits. Many studies were devoted to opening the band gap of graphene, but at the cost of sacrificing conductivity or consuming a large amount of energy. Seeking a graphene-like material with a suitable band gap has become a new solution. For example, FETs using TMDs (such as MoS_2_, WS_2_, MoS_2_, WSe_2_, and ZrS_2_) as channel materials have high switching ratios, low subthreshold swings, and high carrier mobility [401]. Radisavljevic et al. [402] used a 30 nm-thick HfO_2_ gate dielectric to demonstrate a room-temperature single-layer MoS_2_ mobility of at least 200 cm^2^V^−1^s^−1^ and demonstrated transistors with current on/off ratio of 10^8^. In addition, 2D TMDs and black phosphorus have high flexibility, which is suitable for flexible electronic devices.

#### 15.2.2. Optoelectronic Devices

**Figure 63:**
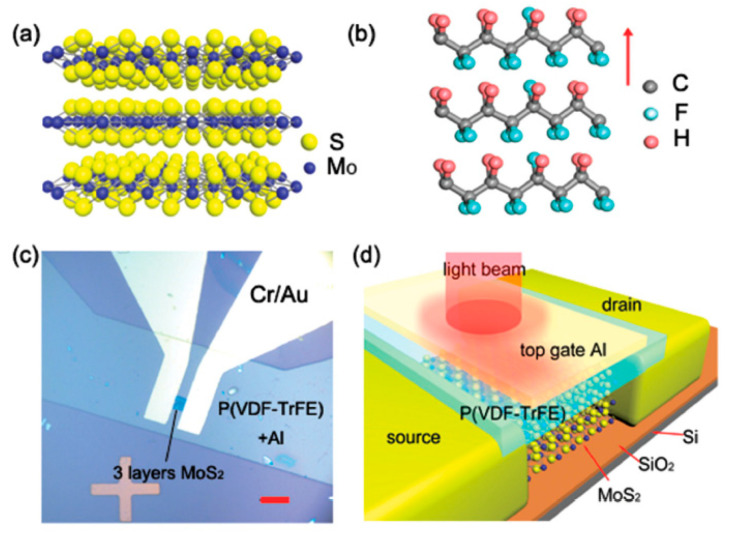
Fabrication and structure of few-layer MoS_2_ photodetector. (**a**) Schematic structure of triple-layer MoS_2_; (**b**) Schematic structure of P(VDF-TrFE) ferroelectric polymer; (**c**) Optical image of the whole device; (**d**) 3D schematic view of the triple-layer MoS_2_ photodetector with monochromatic light beam [406].

In recent years, 2D materials have been highly valued for their unique optical properties, and have been applied to optoelectronic devices, including photodetectors, optical modulators, and lasers, etc. Xia et al. [403] prepared the first graphene photodetector using graphene transistors, which has wider light detection band and ultra-fast optical response compared with traditional photodetectors based on silicon and III-V materials. Later, Casalino et al. [404] successfully enhanced the optical absorption capability of the graphene/silicon interface by adding an enhanced optical resonator to the device and observed a wavelength-dependent photo response with external (internal) responsivity ~20 mA/W (0.25 A/W). Black phosphorus has great potential in the application of optical devices because of its direct energy gap and high light absorption efficiency. For example, a black phosphorus transistor with graphene as the transparent top gate has an optical response of up to 657 mA/W and a high-sensitivity frequency bandwidth exceeding 3 GHz [405]. Wang et al. [406] developed a MoS_2_ photodetector with a ferroelectric gate (as shown in Figure 63) in which three layers of MoS_2_ are used as photosensitive semiconductor channels, and the device exhibits a responsivity of 2570 A/W. Xie et al. [407] controlled the deviation from the perfection of the atomic lattice to narrow the band gap of the multilayer MoS_2_, realizing an ultra-wide detection range from 445 nm (blue light) to 2717 nm (mid-infrared).

#### 15.2.3. Energy Storage Devices

**Figure 64:**
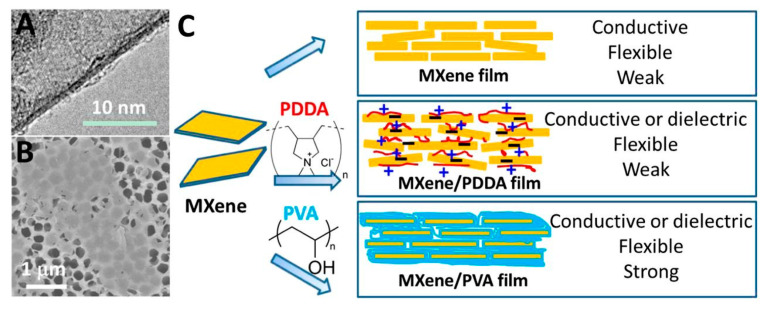
(**a**) TEM and (**b**) SEM images of MXene flakes after delamination and before film manufacturing. (**c**) A schematic illustration of MXene-based functional films with adjustable properties [409].

2D materials can be applied to batteries or supercapacitors for energy storage since it has high specific surface area, short diffusion path, and good conductivity. Graphite is currently used as a negative electrode material of lithium ion batteries, which has a good cycle life but low theoretical specific capacity (372 mAh·g^−1^). The study found that the cycle stability of electrode materials TMDs can be significantly improved by combining TMDs with graphene to construct highly conductive flexible carbon nanocomposites [26,408]. Supercapacitors are very promising energy storage and conversion devices, which have higher specific power than that of lithium-ion batteries. The ideal supercapacitors should have high energy and power density. The surface area and conductivity of electrode materials are the main factors affecting the performance of supercapacitors. 2D materials have great application advantages in this field, such as graphene, TMDs (such as MoS_2_), LDHs (such as CoAlLDHs), MXenes (such as Ti_2_C), etc. [409] (see Figure 64a–c). It is even expected to be used in flexible supercapacitors for wearable devices.

#### 15.2.4. Sensors

**Figure 65:**
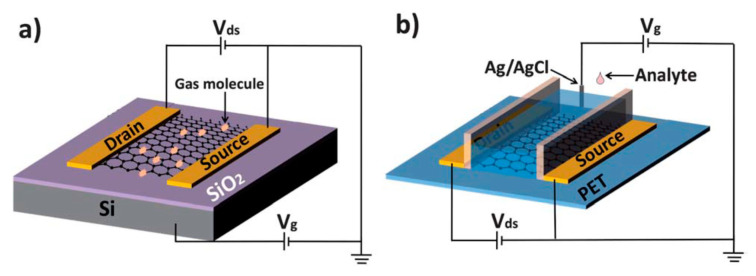
(**a**) Typical back-gate graphene-based field-effect transistor (GFET) on Si/SiO_2_ substrate used as gas sensor, and (**b**) typical solution-gate GFET on flexible polyethylene terephthalate (PET) substrate used as chemical and biological sensor in aqueous solution [412].

In addition to their excellent electrical conductivity and high specific surface area, some 2D materials also have sensitivity to external stimuli, making them promising prospects in the field of sensing. Li et al. [410] fabricated single and multilayer MoS_2_ film-based FETs by mechanical exfoliation for NO gas detection at room temperature. The resistance of the FET channel increases as the amount of NO adsorbed increases, reflecting the amount of NO gas. Li et al. [411] prepared NH_3_ gas sensors using WS_2_ nanosheets and improved the selectivity of interfering substances such as methanol, ethanol, and formaldehyde. The electronic sensors realize the detection by the change of the conductivity of the channel material caused by the interaction between the channel material and the object to be detected. The fluorescence sensors use the interaction between the fluorescence probe and the object to cause the amplification or quenching of fluorescence. Graphene and its derivatives, TMDs, BP, MOFs, etc., can be used for electronic sensors and fluorescent sensors (see Figure 65a,b) [412].

**Part Four: Metrology Technologies**

## 16. Advanced Characterizations for Ultra-Miniaturized CMOS

With the continuous reduction of the structure of semiconductor devices and the continuous optimization of the process materials, the process materials tend to be smooth and thin with fine structure. In order to produce more efficient and high-performance devices, we need to find more suitable measurement methods to characterize different performance parameters of materials and structures. With the optimization of the device, the capability of measurment equipments is constantly being improved and they are developed in a more convenient, accurate, and comprehensive direction without damage to the devices. Here, we introduce several advanced characterizations for ultra-miniaturized CMOS.

### 16.1. Future Scanning Electron Microscope (SEM) for Nano Analysis

**Figure 66:**
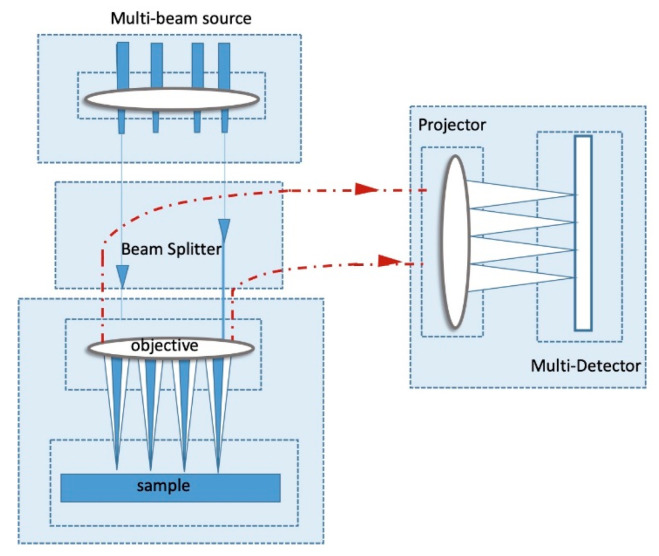
Schematic drawing of the multi-beam SEM.

Traditional SEM is used in process research and development because of fast results and direct way of imaging. The resolution of SEM is improved by increasing the voltage intensity, reducing the size of electron beam spot, and aberration correction. This is a convenient section imaging method, which can achieve the image calibration in 3D standard in a short time. High-throughput scanning electron microscopy increases the scanning speed while maintaining the original best resolution and contrast. The total detection bandwidth of the multi-beam scanning electron microscope is the single beam bandwidth multiplied by the beam number as shown in Figure 66. The increase of the bandwidth can make the primary electron produce the secondary electronic signal more effectively and reduce the damage to the sample caused by the electron beam. A multi-beam scanning electron microscope with 331 electron beams was successful applied. This multi-beam scanning electron microscope method will be able to meet the throughput requirements of future semiconductor applications [413,414].

Critical dimension testing is an important part of the IC front-end process. CD-SEM has the advantages of non-contact condition. It can be used to monitor the critical size and performance in the process of chip manufacturing. In the process of less than 10 nm, the new advanced CD-SEM technology is effectively applied to logic vias, complex overlay, HAR contacts, 3D morphology, etc. [415,416,417]

### 16.2. Atomic Force Microscope (AFM) for 3D Analysis

**Figure 67:**
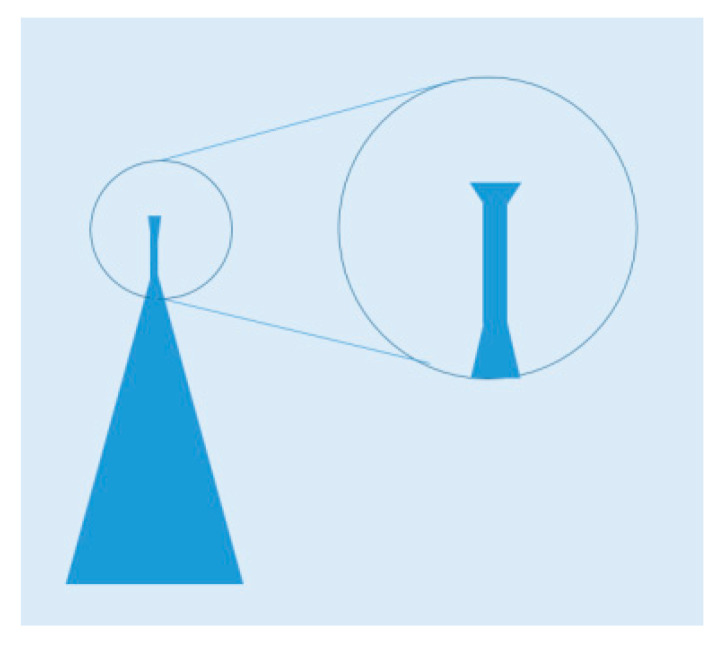
The shape of 3D AFM.

Although the traditional AFM can scan the three-dimensional morphology, it is not a true three-dimensional morphology because the probe cannot touch the surface of sidewalls. It is also impossible to measure the spatial size, side wall analysis, and 3D doping analysis. The 3D AFM probe shape is shown in Figure 67. 3D-AFM is used for high-resolution sidewall imaging, overhang profiles, and critical angle measurements. The tilted z-scan XY and z-scan systems overcome the challenges of conventional and flaps in accurate wellbore analysis [418]. Three-dimensional atomic force microscopy (3D-AFM) or critical size atomic force microscopy (CD-AFM) techniques can be used for in-line monitoring and engineering analysis. It overcomes the defects of scanning electron microscopy (SEM) in measuring CD at the bottom of the structure and can test data more accurately than OCD (optical critical dimension). It saves the derivation of optical measurement and enables automatic analysis and efficient production [419].

### 16.3. 3D Atom Probe Tomography (APT)

In ultra-miniaturized devices, the element composition and atomic scale doping affect the device performance. Common detection equipments are high-resolution transmission electron microscopy (HRTEM) and scanning transmission electron microscopy (STEM), equipped with electron energy loss spectroscopy (EELS) and energy dispersive X-ray spectroscopy (EDX). These devices are two-dimensional imaging and are unable to obtain complex three-dimensional structures at the nanoscale. Secondary ion mass spectrometry (SIMS) is used to characterize the element depth distribution, with poor lateral resolution.

**Figure 68:**
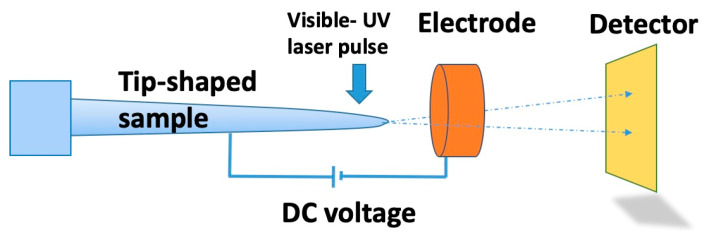
Conventional atom probe tomography.

Atomic probe tomography (APT) has become an important tool for the characterization of 3D semiconductor devices. It can not only achieve a high-resolution map of the elements in the space, but also can identify more light elements. The interface of different materials is clearly visible, which can effectively help us understand the size and spatial structure of these devices. Figure 68 shows the schematic diagram of traditional APT [420,421,422].

The complexity of ultra-miniaturized CMOS structure is a challenge for accurate measurement of dopants. The distortion and uncertainty of geometric reconstruction are caused by local amplification and trajectory overlap of mixed materials. Due to the irregular shape of the nanoscale, the signal distortion can be reduced by quantitative sample preparation. In addition, laser-assisted atomic probe tomography can eliminate the heat transients caused by the near-ultraviolet laser. APT also needs to combine more devices to improve test results and analysis algorithms to restore 3D structures more realistically. The technology is still being developed and improved in the semiconductor field.

### 16.4. Optical Critical Dimension (OCD)

Optical scattering measurement also known as optical critical size measurement is used to maintain the surface topography and can achieve the measurement with high-performance thin films and high-precision nanometer boundaries. It can also be lossless, which is a rapid way for the multilayer structure and internal measurement. The principle is to illuminate the target sample with polarized light and collect the reflection spectrum. Methods of measurement include spectral ellipsometer (SE), miller matrix (MM) SE, and normal incident polarized light reflection, or multi-angle, multi-wavelength OCD. The method of multi-azimuth scattering measurement increases the information of data by collecting and analyzing the spectra of gratings in different directions. It is necessary to obtain 12 or more parameters at the same time to describe the 3D structure of the device.

With the development of ultra-miniaturized CMOS technology, the existing light scattering method is only suitable for the measurement of repetitive dense structures, but not for the measurement of isolated or complex periodic structures. This presents OCD with new challenges, for example, for multi-membrane parameter measurements, 3D measurements, or obtaining random structural parameters in FinFET. The reduction of key dimensions makes the device less sensitive. In addition, the eigenvalue will be disturbed, thereby affecting the refractive index (RI) and extinction coefficient (n). This is improved by adding optical channels for incident angles, azimuths, Mueller matrices, etc., but this increases the difficulty of modeling, which increases the time and cost of early development. It is reported that laser-pumped plasma sources will be used due to its high signal-to-noise ratio and advantages in extending available spectra to the infrared band. Its application can further reduce the uncertainty of some parameters and improve the sensitivity to others, but it cannot completely solve many of the above challenges [423,424,425,426,427].

### 16.5. Hybrid Metrology

With the development of ultra-miniaturized CMOS technology, the introduction of new materials and the three-dimensional structure of the process results in the diversification of the characterization of the structural characteristics. For example, TEM, SEM, AFM, OCD, APT, CDSAXS, and other test equipment are described from different characteristics. However, these data are relatively homogeneous and may have measurement errors. There is no tool that meets all the metrology requirements of ultra-miniaturized CMOS. Test equipment with high efficiency and high precision are needed for the next generation of transistor manufacturing [428,429,430,431,432].

Hybrid metrology combines different testing tools to eliminate measurement errors and improve the accuracy of test data. We use hybrid metrology to understand the material properties and interactions of complex 3D device structures to avoid structural errors and test limitations. We usually make local measurements on the basis of obtaining a large number of local data model databases. For example, XRD, SIMS, XPS, and TEM are combined to analyze the repeatedly arranged data. For example, atom probe chromatography, TEM, and OCD are combined to construct a nano-scale hybrid metrology solution for three-dimensional structures. The combination of metrology and artificial intelligence and the automation of testing tools can make measurement and data analysis more efficient.

### 16.6. X-ray Metrology Technologies

With the decrease of technology node and the change of device structure, the influence of film thickness on device performance is very important. Traditional film metrology cannot provide higher resolution. X-rays have more applications. For example, more parameters such as grain size, grain direction, component, strain, etc., of IC transistor channel materials, interconnect materials, workfunction materials, and other new materials are analyzed quantitatively and non-destructively. The technologies are developed through XRR (x-ray reflectivity), XRDI (X-ray diffraction image), XRD (X-ray diffraction), HRXRD (high-resolution X-ray diffraction), LEXES (low-energy electron-induced X-ray emission spectrometry), CD-SAXS, and GISAXS.

XRR ((X-ray reflectivity) is used to measure the thickness, density, surface, and interface roughness of multi-layer opaque films. Due to its non-destructive measurement method, it is currently applied to in-line monitoring. In order to better apply to the in-line detection of advanced characterization for ultra-miniaturized CMOS, the incident angle and focused beam are increased to reduce the measurement area and improve the detection efficiency.

XRD (X-ay diffraction) is a non-destructive analysis method that reveals the analysis of the polycrystalline thin film, and the size of the crystallinity, crystal grain size, residual stress, the preferred orientation of chemical composition, and physical characteristics. Bruker’s latest in-plane stage module can measure the in-plane grain size and crystal direction of 1.5–10 nm HKMG structured ultra-thin films, and these parameters determine the dielectric constant, resistivity, and other film characteristics. The strain of channel material of FinFET and GAA structure directly restricts the material mobility.

HRXRD (high-resolution X-ray diffraction) is used to measure the strain, strain relaxation degree, doping concentration, composition, and thickness parameters of single-crystal thin films. Repeated patterns of tiny structures can also be measured. The latest large spot stage of Bruker company can automatically locate and measure material parameters of tiny structures, such as solid pad, 2D pad, 3D pad, FinFET, GAA, etc. Characterization of III-V fins at sub10 nm has also been confirmed. There is no other testing technique that can do this. Therefore, HRXRD is an important method for the characterization of 10 nm and above nodes [433,434,435,436,437,438,439,440,441,442].

**Figure 69:**
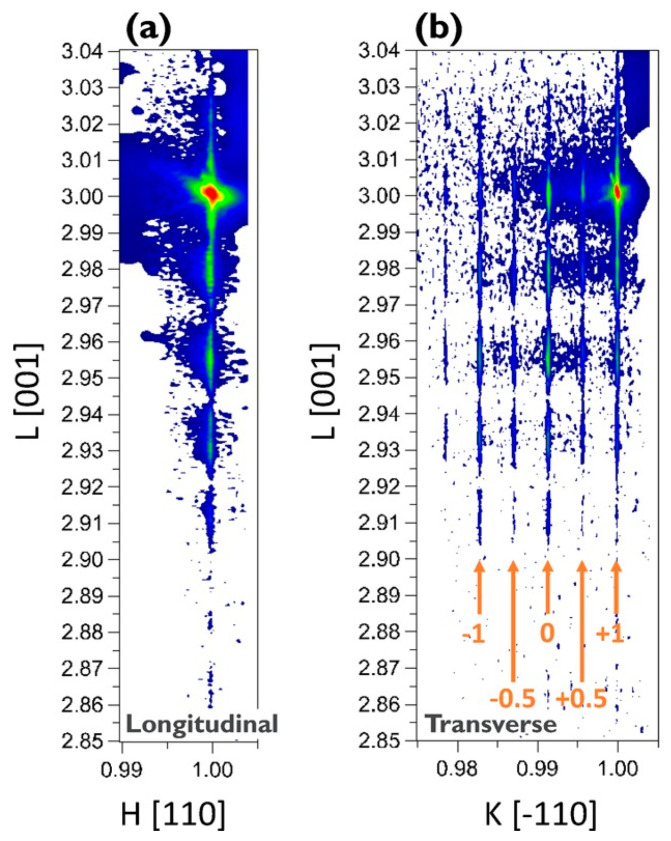
HRRLMs of (113) reflection obtained from fins with width of 20 nm in (**a**) longitudinal and (**b**) transverse direction [441].

Figure 69 shows HRRLMs around (113) reflection obtained SiGe fins. The maps provide information about the lattice parameters in parallel and perpendicular growth direction as well as fin pitch can be estimated from the spacing between first-order grating rods in the figure. This type of measurement has been also used to analysize III-V fins in sub-10 nm [441].

Critical dimension small-angle X-ray scattering (CD-SAXS) is used to measure the average shape, edge roughness, and line spacing of periodic nanostructures. Grazing incidence small-angle X-ray scattering (GISAXS) is another way for measuring critical dimensions by using grazing incidence small-angle X-ray scattering [443].

**Figure 70:**
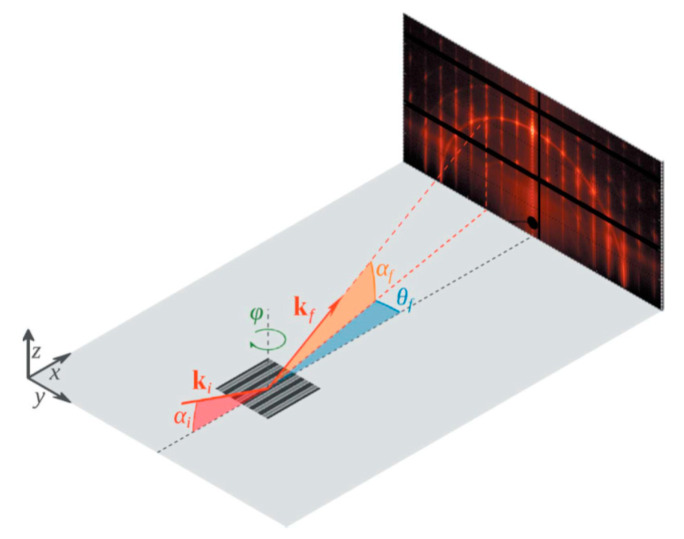
Geometry of grazing incidence small-angle X-ray scattering (GISAXS) experiments [443].

The geometry of GISAXS measurements is displayed in Figure 70. GISAXS is suitable for small targets that are sensitive to the grating line profile. GISAXS technique can be used to extract structural parameters of the gratings by considering the photon energy during scanning and the scattering intensity.

Unlike traditional measurements, the CD-SAXS is not affected by changes in the size of the optical properties. It is based on the electron density and, therefore, it has a higher resolution. According to the requirements of three-dimensional structure features, CD-SAXS is better than OCD when the critical size is less than 10 nm. At the same time, CD-SAXS does not need to develop a structural model. CD-SAXS is nondestructive and is a promising technique for characterization of nanodevices [444,445,446].

The XRF/XPS (X-ray fluorescence/X-ray photoelectron spectroscopy) interegrated tool is used to fast determine the composition and chemical composition of material surfaces. It can be used for the identification of semiconductor surface contamination. XRDI (X-ray diffraction image) is used in the detection of wafer-level defects, e.g., slips.

### 16.7. Artificial Intelligence in Metrology

**Figure 71:**
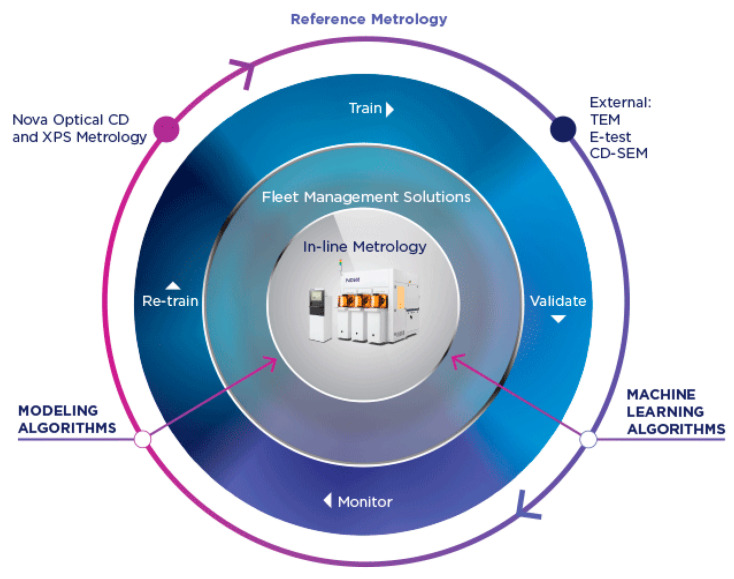
An image of NOVAFit™ applications for advanced nano-scale measurements [447].

The newly developed artificial intelligence has been increasingly applied in semiconductor metrology in recent years. A promising technical solution for solving the most difficult metrology challenges is provided by machine learning at 10 nm node and below, which will cooperate with metrology tools instead of completely replacing them. NOVAFit^TM^ is a presentative software developed by NOVA Company. It can be employed as a supplementary method for machine learning technique, which can help to predict the CD values of Fins according to the in-line performance as shown in Figure 71 [447]. Combined with this technique, the electrical resistance in interconnects can be obtained by taking advantage of data from both OCD and electrical tests. Metrology capabilities are enhanced, and the consumption of time is greatly reduced in analyzing complex 3D devices, which has a high aspect ratio by using this software.

## 17. Conclusions

This article contains four parts with the following outcomes:

In part one, gate-all-around (GAA) FET has been presented as a strong candidate for 3 nm technology due to its high performance and superiority in the control of short channel effects. The structure of both horizontal GAAFET (hGAAFET) and vertical GAAFET (vGAAFET) have been discussed and their performance was compared. The main advantage of vGAAFETs over hGAAFETs is that they have more integration freedom in the vertical direction than hGAAFETs do. The limitations of gate length, spacer width, and source/drain contact area for hGAAFETs can be relaxed for vGAAFETs and then integration density increased. Both bottom-up and top-down fabrication methods for GAAFETs have been presented. For the top-down approach, a multilayer of p-Si/i-SiGe/p-Si can be initially grown where this stack is patterned and vertically etched to form pillar. Later, the SiGe layer is laterally etched to form the channel layer. For mobility, transistor InAs vGAAFETs have been proposed as channel material for nMOS, where for pMOS, Ge or SiGe are supposed to be integrated. Finally, TFET design has been tackled and discussed.

Later, the importance of device simulation for the advanced nano transistor designs has been highlighted. In this chapter, TCAD device models of different complexity, precision, and accuracy are presented. Depending on specific device scales, a quantum-mechanical model or a semi-classical model could be adopted.

In part two, the discussions begin with state-of-the-art lithography of nano-scale patterns using 193 nm immersion with self-aligned double pattern (SADP) and self-aligned quadruple pattern (SAQP) technology for 7 nm technology nodes and behind. EUV lithography is the competing technology that simplifies the patterning process for nano-scale transistors. Meanwhile, EUV still has issues with resists and mask infrastructure as well as power source, which have to be solved before high-volume manufacturing in near future.

The epitaxy of SiGe as stressor material in source/drain regions have been presented. The pattern dependency of the growth over the 8-inch wafer was also discussed. In order to have uniform SiGe epitaxy, a uniform chip layout is needed to minimize the pattern dependency of the growth.

Epitaxy of GAAFETs contains stack of Si/SiGe/Si or Ge/SiGe/Ge where Si, Ge, or SiGe can be the channel of the transistor. Si or SiGe material could be selectively etched by using TMAH solution mixed by ACT^®^ SG-201.

In the nanowire transistors, traditional ion implantation cannot directly be applied, and new doping strategies e.g., monolayer and plasma doping, can be used to dope the GAAFETs.

Apart from doping process, Ge, Si, or SiGe are employed to inhibit the channeling or transient enhanced diffusion (TED) effect in UJS module. Moreover, Ge or As is also proved to be favorable to decrease the contact resistance. Since the structure of CMOS has evolved into three dimensions, Ti silicide has re-acted as standard contact schemes due to its better thermal stability and lower contact resistivity.

Pre-amorphous implantation (PAI) is reported to effectively improve the performance of Ti silicide. It has demonstrated that when a low-energy pre-amorphous Ge implantation is applied on the contact area, the conductivity is improved to an extremely low value of about 1.5 × 10^−9^Ωcm.

HfO_2_ has been implemented as high-k material in nanoscale transistors for more than 20 years due to its high dielectric constant and a relatively large bandgap. However, HfO_2_ suffers from the thermal instability of HfO_2_/Si interface in nano transistors. A SiO_x_ interlayer between HfO_2_ and Si substrate is a remedy to the interfacial imperfection. Meanwhile, the thickness of SiO_x_ interlayer and high-*k* dielectric have been constantly reduced in each technology node.

New metal-gates like TiAlN and TiN have been integrated in a gate-last process to avoid crystallization of the high-*k* material during the thermal treatments.

ALD W in α-phase is mainly integrated as metal electrode filling for nano transistors. Using B_2_H_6_ and WF_6_ show lower growth rate, lower resistivity, and better gap-filling capability in gate trenches with high aspect ratio and better control of short-channel effects.

Interconnect material in microelectronic circuit also becomes an issue since the CD becomes narrower and filling the BEOL trench-over-via using classical Cu metal has become a challenge. Recently, studies show Co is a better choice for via prefill with no void-free and it performs EM behavior better compared to Cu since it has higher melting point.

Reliability testing of the processed wafers becomes a crucial issue for IC manufacturers and this subject becomes more critical by scaling down CMOS. To check the novel materials and processes, the reliability analysis gives information about physical mechanism of any problem or degradation.

Another important indicator of the defects and integration issue is random telegraph noise measurements for nano-scaled transistors.

In part three of this article, the material technology in the beyond-Moore era has been considered. The high mobility channel material is a hot topic and among many suggested materials, GeSi, Ge, III-V, and 2D crystals are in sight. The main problem with these materials is their integration with high quality on Si. For III-V devices e.g., FinFET on III-V-OI substrates and hybrid InGaAs/SiGe CMOS on silicon substrates with advanced selective epitaxy has been used.

Among 2D materials, graphene, transition-metal dichalcogenides (TMDCs) such as MoS_2_, WSe_2_, and WS_2_ have high potential for high-production devices in future. For 2D materials with almost no bandgap, there are different methods that can be applied such as narrow strips, bilayers, or more to create a bandgap.

Many promising devices from 2D materials have been manufactured. As an example, a dual-channel FET based on a vertically stacked heterostructure of ultrathin n-type MoS_2_ and p-type WSe_2_ layers for the study of parallel carrier transport (electrons from MoS_2_ and holes from WSe_2_) have been demonstrated.

For example, single-layer MoS_2_ used as the active channel material with a 30 nm-thick HfO_2_ gate dielectric has demonstrated carrier mobility of 200 cm^2^V^−1^s^−1^ and demonstrated transistors with current on/off ratio of 10^8^. The mobility does not degrade at high gate voltages, presenting an important advantage over conventional Si transistors where enhanced surface roughness scattering severely reduces carrier mobility values at high gate-fields.

In part four of this article, metrology technologies, which can be used for on-line monitoring and engineering analysis for nano analysis including SEM techniques, 3D atom force microscope techniques, 3D atom probe tomography, X-ray techniques, optical critical dimension, and hybrid metrology together with artificial intelligence in metrology were discussed. In the production line, there is always a need for a pilot test to measure the critical dimensions of the devices, profile layer of thin film, surface and interface quality, electrical, and optical performance. In order to do these tasks in-line, the characterization should be fast but not destructive, with high precision, and high speed. The famous tools are CD SEM, OCD, 3D AFM, and 3D APT, which can be applied for the 3D device structures.

It is also necessary to use a combination of these characterization techniques, which could give information about more complicated device structures.

There also several X-ray techniques such as XRF, XPS, XRR, XRD, HRXRD, LEXES, CD-SAXS, and GISAXS that are used in-line measurements. These techniques give information about crystalline quality, layer profile (thickness and composition), dopant concentration, and strain relaxation in devices.

Artificial intelligent is a new way to work with metrology. Nowadays, a new software has been introduced, which is capable of solving the metrology challenges for 10 nm node technology and beyond. The new software increases capability for metrology for complex 3D devices with high aspect ratio devices.

## Figures and Tables

**Table 1:** The effective workfunction of different metals grown by atomic layer deposition (ALD) for N type Metal-Oxide-Semiconductor Field-Effect Transistor (NMOSFET).

Metal	Dep. Method	Effective Workfunction	Ref
TiN	thermal ALD	5.3 eV	[146]
Ti_1−x_Al_x_N	PEALD	5.04 eV	[147]
TiC-TiN	PEALD	5.0-4.6 eV	[148]
TiC_x_N_y_	PEALD	4.66 eV	[149]
TaC_y_	PEALD	4.77−4.54 eV	[150]
TiC	PEALD	5.24–4.45 eV	[151]
TaCN	PEALD	4.37 eV	[152]
WC_0.4_	PEALD	4.2 ±0.1 eV	[153]
TiAlC	thermal ALD	4.79–4.49 eV	[154]
TiAlC	thermal ALD	4.4 –4.24 eV	[155]
TaAlC	thermal ALD	4.74–4.49 eV	[156]
TaAlC	thermal ALD	4.65–4.26 eV	[157]
ErC_2_	ALD	3.9 eV	[158]

**Table 2:** Typical applications where InP HEMTs is involved and their features [350].

No.	Application	Performances	Gate Length (nm)	Ref.
1	Receiver and transmitter front-ends	850 GHz band 20 dB average receiver noise figure	25	[351]
2	Multiplier Chain	400 GHz band Peak output power: 6.9 mW	25	[352]
3	Cascode amplifier	17 dB gain Noise 8.3 dB 300 GHz band	30	[353]
4	Optical repeater	Speed 40 Gbits/s	100	[354]
5	Photo receiver	Speed 40 Gbits/s	100	[355]
6	Ultra wideband impulse Radar	24 GHz band	130	[356]
7	UP-Converter	Gain of 1 dB at 64.5 GHz for 1.7 dBm LO power	200	[357]

**Table 3:** Direct Current (DC) and Radio Frequency (RF) performances of some classical InP HEMTs [350].

No.	Gate Length (nm)	$f_{\max}\;(\mathrm{GHz})$	$f_{T}\;(\mathrm{GHz})$	$g_{m}$ (mS/mm)	$I_{\mathrm{DS}\_\max}$ (mA/mm)	Ref.
1	300	229	116	701	≈400	[376]
2	120	340	166	1100	800	[377]
3	100	415	249	1051	724	[378]
4	80	330	310	2630	700	[379]
5	60	478	710	2114	≈650	[380]
6	40	402	491	2000	≈800	[381]
7	30	681	644	1900	≈850	[382]
8	25	1500	610	3100	≈1180	[375]
